# Effects of Percutaneous Coronary Intervention on Death and Myocardial Infarction Stratified by Stable and Unstable Coronary Artery Disease: A Meta-Analysis of Randomized Controlled Trials

**DOI:** 10.1161/CIRCOUTCOMES.119.006363

**Published:** 2020-02-17

**Authors:** Liza Chacko, James P. Howard, Christopher Rajkumar, Alexandra N. Nowbar, Christopher Kane, Dina Mahdi, Michael Foley, Matthew Shun-Shin, Graham Cole, Sayan Sen, Rasha Al-Lamee, Darrel P. Francis, Yousif Ahmad

**Affiliations:** 1 Imperial College London, United Kingdom (L.C., J.H., C.R., A.N.N., C.K., D.M.,M.F., M.S.-S., G.C., S.S., R.A.-L., D.P.F., Y.A.); 2 Columbia University Medical Center, New York (Y.A.).

**Keywords:** acute coronary syndrome, coronary artery disease, fibrinolysis, myocardial infarction percutaneous coronary intervention

## Abstract

In patients presenting with ST-segment–elevation myocardial infarction, percutaneous coronary intervention (PCI) reduces mortality when compared with fibrinolysis. In other forms of coronary artery disease (CAD), however, it has been controversial whether PCI reduces mortality. In this meta-analysis, we examine the benefits of PCI in (1) patients post–myocardial infarction (MI) who did not receive immediate revascularization; (2) patients who have undergone primary PCI for ST-segment–elevation myocardial infarction but have residual coronary lesions; (3) patients who have suffered a non–ST-segment–elevation acute coronary syndrome; and (4) patients with truly stable CAD with no recent infarct. This analysis includes data from the recently presented International Study of Comparative Health Effectiveness with Medical and Invasive Approaches (ISCHEMIA) and Complete versus Culprit-Only Revascularization Strategies to Treat Multivessel Disease after Early PCI for STEMI (COMPLETE) trials.

**Methods and Results:**

We systematically identified all randomized trials of PCI on a background of medical therapy for the treatment of CAD. The ISCHEMIA trial, presented in November 2019, was eligible for inclusion. Data were combined using a random-effects meta-analysis. The primary end point was all-cause mortality. Forty-six trials, including 37 757 patients, were eligible. In the 3 unstable scenarios, PCI had the following effects on mortality: unrevascularized post-MI relative risk (RR) 0.68 (95% CI, 0.45–1.03); *P*=0.07; multivessel disease following ST-segment–elevation myocardial infarction (RR, 0.84 [95% CI, 0.69–1.04]; *P*=0.11); non–ST-segment–elevation acute coronary syndrome (RR, 0.84 [95% CI, 0.72–0.97]; *P*=0.02). Overall, in these unstable scenarios PCI was associated with a significant reduction in mortality (RR, 0.84 [95% CI, 0.75–0.93]; *P*=0.02). In unstable CAD, PCI also reduced cardiac death (RR, 0.69 [95% CI, 0.53–0.90]; *P*=0.007) and MI (RR, 0.74 [95% CI, 0.62–0.90]; *P*=0.002). For stable CAD, PCI did not reduce mortality (RR, 0.98 [95% CI, 0.87–1.11]), cardiac death (RR, 0.89 [95% CI, 0.71–1.12]; *P*=0.33), or MI (RR, 0.96 [95% CI, 0.86–1.08]; *P*=0.54).

**Conclusions:**

PCI prevents death, cardiac death, and MI in patients with unstable CAD. For patients with stable CAD, PCI shows no evidence of an effect on any of these outcomes.

## Abstract 2

Supplemental Digital Content is available in the text.

**What Is Known**

- Percutaneous coronary intervention (PCI) reduces mortality in patients with ST-segment–elevation myocardial infarction.
- The benefit of PCI in other forms of coronary artery disease has been controversial.

**What the Study Adds**

- Three groups of unstable coronary artery disease were identified: patients post–myocardial infarction who did not receive immediate revascularization; patients who have undergone primary PCI for ST-segment–elevation myocardial infarction but have residual coronary lesions; and patients who have suffered a non–ST-segment–elevation acute coronary syndrome.
- PCI prevents death, cardiac death, and myocardial infarction in patients presenting with unstable coronary artery disease.
- In patients with truly stable coronary artery disease, PCI shows no evidence of an effect on any of these outcomes.

**See Editor’s Perspective**

In patients presenting with ST-segment–elevation myocardial infarction (STEMI), percutaneous coronary intervention (PCI) reduces mortality when compared with the alternative strategy of fibrinolysis.^1,2^ In other forms of coronary artery disease (CAD), however, it has been controversial whether PCI reduces mortality.

Outside of the setting of an ongoing STEMI lies a broad spectrum of clinical entities. One category is patients who have undergone successful primary PCI for STEMI but have residual coronary lesions (multivessel disease following STEMI). Another category is patients who have suffered an acute coronary syndrome but without ST-segment elevation (non–ST-segment–elevation acute coronary syndrome [NSTEACS]). A third category is patients who have suffered an acute myocardial infarction (MI) but who have not been immediately revascularized (unrevascularized post-MI), although this is less commonly seen in modern clinical practice. Finally, patients may have truly stable CAD. The first 3 categories (multivessel disease following STEMI, NSTEACS, and unrevascularized post-MI) can together be considered as unstable CAD.

Some previous meta-analytic work in this field^3^ had considered the unrevascularized post-MI state as stable CAD, despite patients having suffered a recent MI. In the modern era, unrevascularized post-MI patients are no longer considered to be a similar group to patients without a history of MI.

The results of 2 large randomized controlled trials (RCTs) in different CAD settings have recently become available: the COMPLETE trial,^4^ examining PCI for multivessel disease following STEMI, and the International Study of Comparative Health Effectiveness with Medical and Invasive Approaches (ISCHEMIA) trial,^5^ examining PCI for patients with stable CAD.

The purpose of this meta-analysis is to provide an updated, comprehensive assessment of the effect of PCI on mortality and MI, using a modern classification which distinguishes stable CAD from unstable CAD (multivessel disease following STEMI, NSTEACS, and unrevascularized post-MI).

## Methods

The data that support the findings of this study are available from the corresponding author on reasonable request.

### Search Strategy

Four individual search strategies were employed to identify, respectively, trials in unrevascularized post-MI; multivessel disease following STEMI; NSTEACS; and stable CAD. We searched PubMed, EMBASE, Medline, OVID Journals, and CENTRAL (Cochrane Central Register of Controlled Trials) until November 2019 for randomized controlled trials (RCTs) relating to the following keywords: acute coronary syndrome, non-ST elevation myocardial infarction (NSTEMI), ST elevation MI (STEMI), coronary artery disease, ischemic heart disease, optimal medical therapy, conservative therapy, percutaneous coronary intervention, revascularization, and percutaneous transluminal coronary angioplasty. The MESH terms and search strategies are detailed in the Online Appendix in the Data Supplement. We also hand-searched the reference lists of existing meta-analyses and review articles to identify further eligible trials. We also included the ISCHEMIA trial, which was recently presented at the American Heart Association Scientific Sessions. Two independent reviewers performed the search and literature screening (L. Chacko and C. Kane), and this was duplicated by a third author (M. Foley). Any disputes were resolved by a senior author (Y. Ahmad).

### Study Categories

We addressed randomized trials of 4 categories of CAD:

1. Multivessel disease following STEMI: patients who underwent successful primary PCI for STEMI and had residual coronary lesions, and who were randomized to PCI versus no PCI for those residual lesions.
2. NSTEACS: patients who had suffered an acute coronary syndrome but without ST-segment elevation, and were randomized to either invasive or conservative therapy.
3. Unrevascularized post-MI: patients who had suffered an acute MI but who had not undergone immediate revascularization. Patients were then randomized to medical therapy or delayed revascularization with PCI. Both STEMI and NSTEMI were considered in this category
4. Stable CAD: patients with truly stable coronary artery disease, who did not meet any of the other above categories and were randomized to invasive or conservative therapy.

### Inclusion and Exclusion Criteria

Studies were eligible if they randomized patients to PCI versus conservative therapy without PCI and they reported outcomes of mortality and MI. NSTEACS trials were only eligible if they compared invasive versus conservative strategies and not if they compared early versus late invasive strategies. For multivessel disease following STEMI, trials were eligible if they reported clinical outcome data following randomization to complete revascularization with PCI or culprit-only revascularization with medical therapy for the residual CAD. For NSTEACS, trials were included if they randomized patients to invasive or conservative therapy (as no trials made a distinction between PCI and CABG in this setting). For stable CAD, trials in which revascularization could be achieved by either PCI or coronary artery bypass graft were included, with results combined to invasive therapy, and compared with conservative therapy.

### End Points

The primary end point is all-cause mortality. The secondary end points are cardiovascular mortality and MI, as prespecified by the individual trials included. We did not differentiate between periprocedural and spontaneous MI. The end points were assessed using at least 1-year follow-up if available, or using the primary publication of each study. Sensitivity analyses using the longest follow-up data available were also performed.

### Data Extraction and Analysis

Three authors (L. Chacko, C. Kane, and C. Rajkumar) independently extracted from each trial publication the event counts for all-cause mortality, cardiovascular mortality, and MI. Any disputes were resolved by a senior author (Y. Ahmad). If studies did not provide the event counts, data were extracted from Kaplan-Meier curves by digitization of the survival curves which were combined with the numbers at risk to derive the number of events, using the R package reconstructKM. We performed a random effects meta-analysis of each clinical scenario (unrevascularized post-MI, multivessel disease following STEMI, NSTEACS, and stable CAD). We also considered all unstable CAD grouped together.

Any interaction between the choice of follow-up time and the effect size was explored by fitting a random-effects model using the trial type and trial as nested random effects and the choice of trial time as a moderator. Publication bias was assessed with a Funnel plot, with tests for publication bias only being performed in the event of at least 10 trials being included in an analysis.^6^ Included studies were assessed using the Cochrane Risk of Bias tool.^7^ The risk of bias assessment was conducted in duplicate separately by 2 authors (A.N. Nowbar and D. Mahdi), with disputes resolved by a senior author (Y. Ahmad).

All statistical analyses were performed using the statistical programming environment R with the metafor package. We used the *I*^2^ statistic to assess heterogeneity.^8^ Values are expressed as mean±SD unless otherwise stated. A *P* value of <0.05 was considered statistically significant. Results were reported in accordance with the Preferred Reporting Items for Systematic Reviews and Meta-Analyses (PRISMA) guidelines^9^ and was prospectively registered at the International Prospective Register of Systematic Reviews (CRD42019148397).

## Results

**Figure 1.**
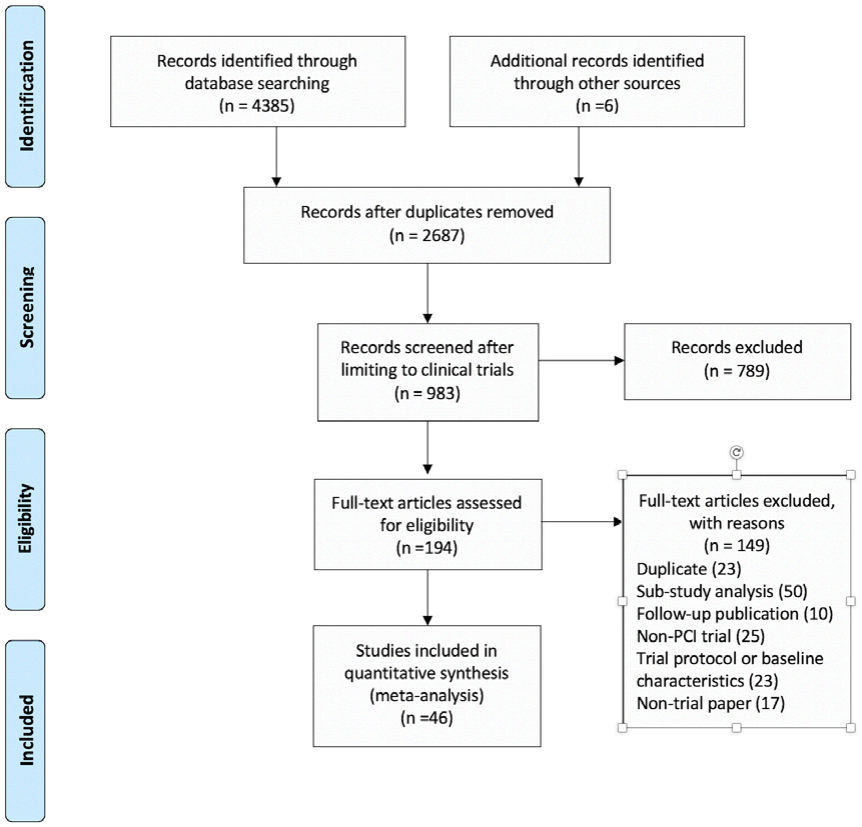
**PRISMA (Preferred Reporting Items for Systematic Reviews and Meta-Analyses) flowchart.** PCI indicates percutaneous coronary intervention.

Forty-six RCTs totalling 37 757 patients (18 793 randomized to invasive therapy and 18 964 randomized to conservative therapy) met the search criteria (see Figure 1): 11 trials^10–20^ (5530 patients; 2759 randomized to invasive therapy and 2771 randomized to conservative therapy) for unrevascularized post-MI; 10 trials^4,21–29^ (7244 patients; 3534 randomized to invasive therapy and 3710 randomized to conservative therapy) for multivessel disease following STEMI; 10 trials^30–39^ (10 314 patients; 5150 randomized to invasive therapy and 5164 randomized to conservative therapy) for NSTEACS; and 15 trials^40–54^ (14 669 patients; 7350 randomized to invasive therapy and 7319 randomized to conservative therapy) for stable CAD.

The baseline characteristics of included trials are shown in the Table. The weighted mean-follow-up was 31.3 months overall. For each category, the weighted mean follow-up was 42.4 months for unrevascularized post-MI, 20.2 months for multivessel disease following STEMI, 13.2 months for NSTEACS, and 41.8 months for stable CAD.

**Table.**
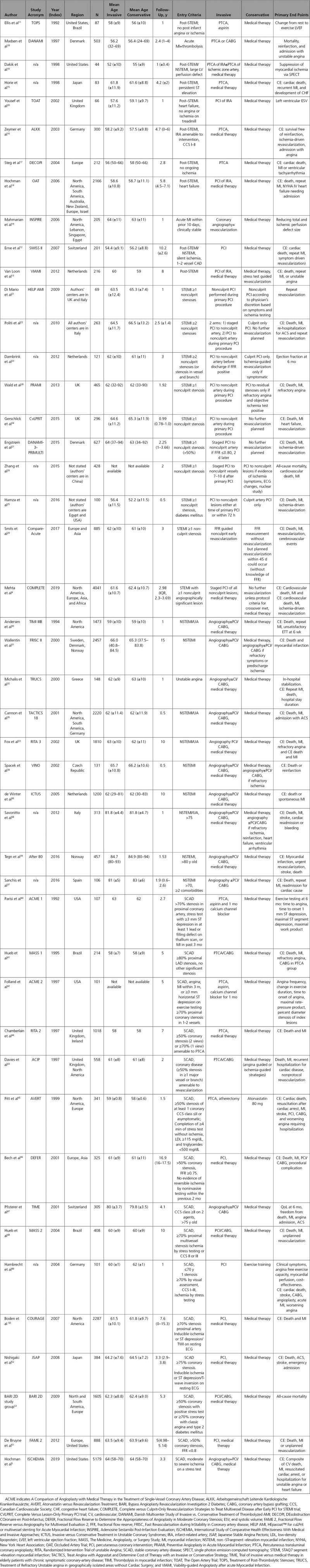
Characteristics of Included Studies

### Quality Assessment

All included trials were randomized clinical trials. The risk of bias of the included RCTs is shown in Online Table I in the Data Supplement. Overall, 15 trials were graded as high risk of bias.

Publication bias was assessed with funnel plots to address the primary outcome of all-cause mortality (see Appendix and Figures I through IV in the Data Supplement), with symmetry of the plot indicating no clear relationship in lack of publication by size of trial and effect estimate. This was performed for each of the 4 separate classifications of CAD, and trim and fill funnel plots are shown in Figures I through IV in the Data Supplement. The *P* values were nonsignificant for the funnel plots for each category of CAD.

### Impact on Mortality

**Figure 2.**
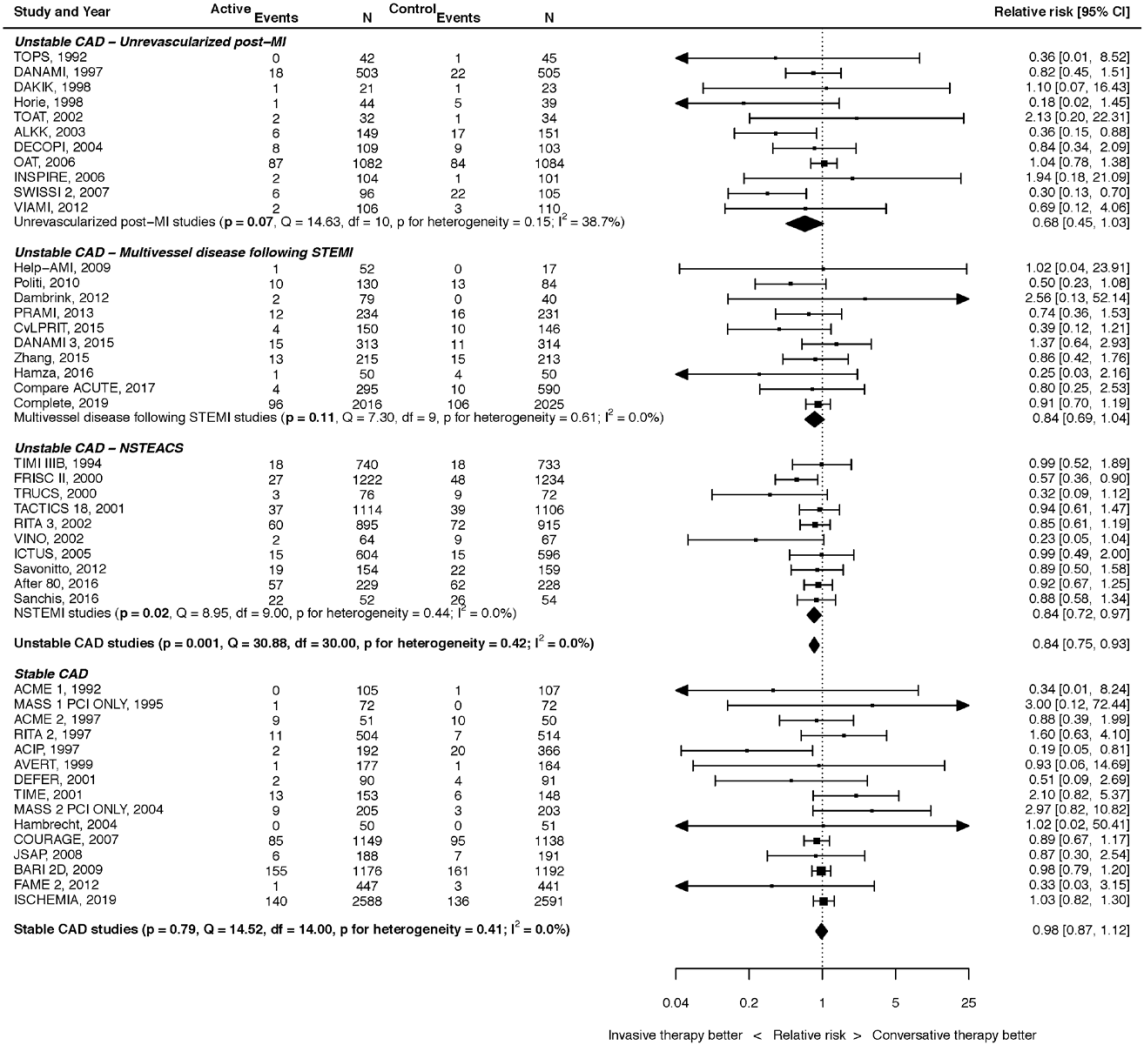
**The effect of percutaneous coronary intervention (PCI) on all-cause mortality.** Results stratified into unstable coronary artery disease (CAD; unrevascularized post–myocardial infarction [MI],^10–20^ multivessel disease following ST-segment–elevation myocardial infarction [STEMI],^4,21–29^ non-ST segment–elevation acute coronary syndrome [NSTEACS]^30–39^) and stable CAD.^40–54^

A summary of the results for the effect of PCI on mortality in CAD is shown in Figure 2.

For unrevascularized post-MI, the effect of PCI on mortality was relative risk (RR) of 0.68 (95% CI, 0.45–1.03; *P*=0.07). There was moderate heterogeneity (*I*^2^=38.7%). For multivessel disease after STEMI, the effect of PCI on mortality was RR, 0.84 (95% CI, 0.69–1.04; *P*=0.11). There was no heterogeneity (*I*^2^=0.0%). For NSTEACS, the effect of PCI on mortality was RR, 0.84 (95% CI, 0.72–0.97; *P*=0.02). There was no heterogeneity (*I*^2^=0.0%). When considered together, PCI for unstable CAD led to a 16% reduction in all-cause mortality (RR, 0.84 [95% CI, 0.75–0.93]; *P*=0.001). There was no heterogeneity (*I*^2^=0.0%).

For stable CAD, there was no effect of PCI on mortality, with RR, 0.98 (95% CI, 0.87–1.1; *P*=0.75). There was no heterogeneity (*I*^2^=0.0%).

### Impact on Cardiovascular Mortality

**Figure 3.**
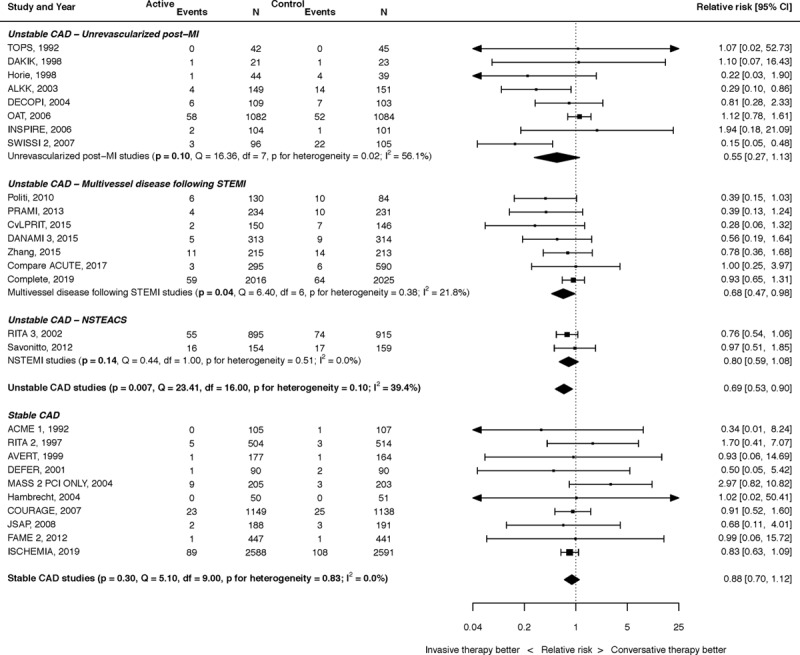
**The effect of percutaneous coronary intervention (PCI) on cardiovascular mortality.** Results stratified into unstable coronary artery disease (CAD; unrevascularized post–myocardial infarction [MI],^10–12,14–17,19^ multivessel disease following ST-segment–elevation myocardial infarction [STEMI],^4,22,24,26–29^ non-ST segment–elevation acute coronary syndrome NSTEACS^33^) and stable CAD.^40,44–46,48–52,54^

A summary of the results for the effect of PCI on cardiovascular mortality in CAD is shown in Figure 3.

For unrevascularized post-MI, the effect of PCI on cardiovascular mortality was RR, 0.55 (95% CI, 0.27– 1.13; *P*=0.010). There was significant heterogeneity (*I*^2^=56.1%). For multivessel disease following STEMI, there was a significant reduction in cardiovascular mortality with RR, 0.68 (95% CI, 0.47–0.98; *P*=0.04). There was mild heterogeneity (*I*^2^=21.8%). For NSTEACS, only 2 trials reported cardiovascular mortality (RR, 0.80 [95% CI, 0.59–1.08]; *P*=0.14), with no heterogeneity (*I*^2^=0.0%). When considered together, PCI for unstable CAD led to a 31% reduction in cardiovascular mortality (RR, 0.69 [95% CI, 0.53–0.90]; *P*=0.007). There was moderate heterogeneity (*I*^2^=39.4%).

For stable CAD, there was no effect of PCI on cardiovascular mortality, with RR, 0.89 (95% CI, 0.71–1.12; *P*=0.33). There was no heterogeneity (*I*^2^=0.0%).

### Impact on MI

**Figure 4.**
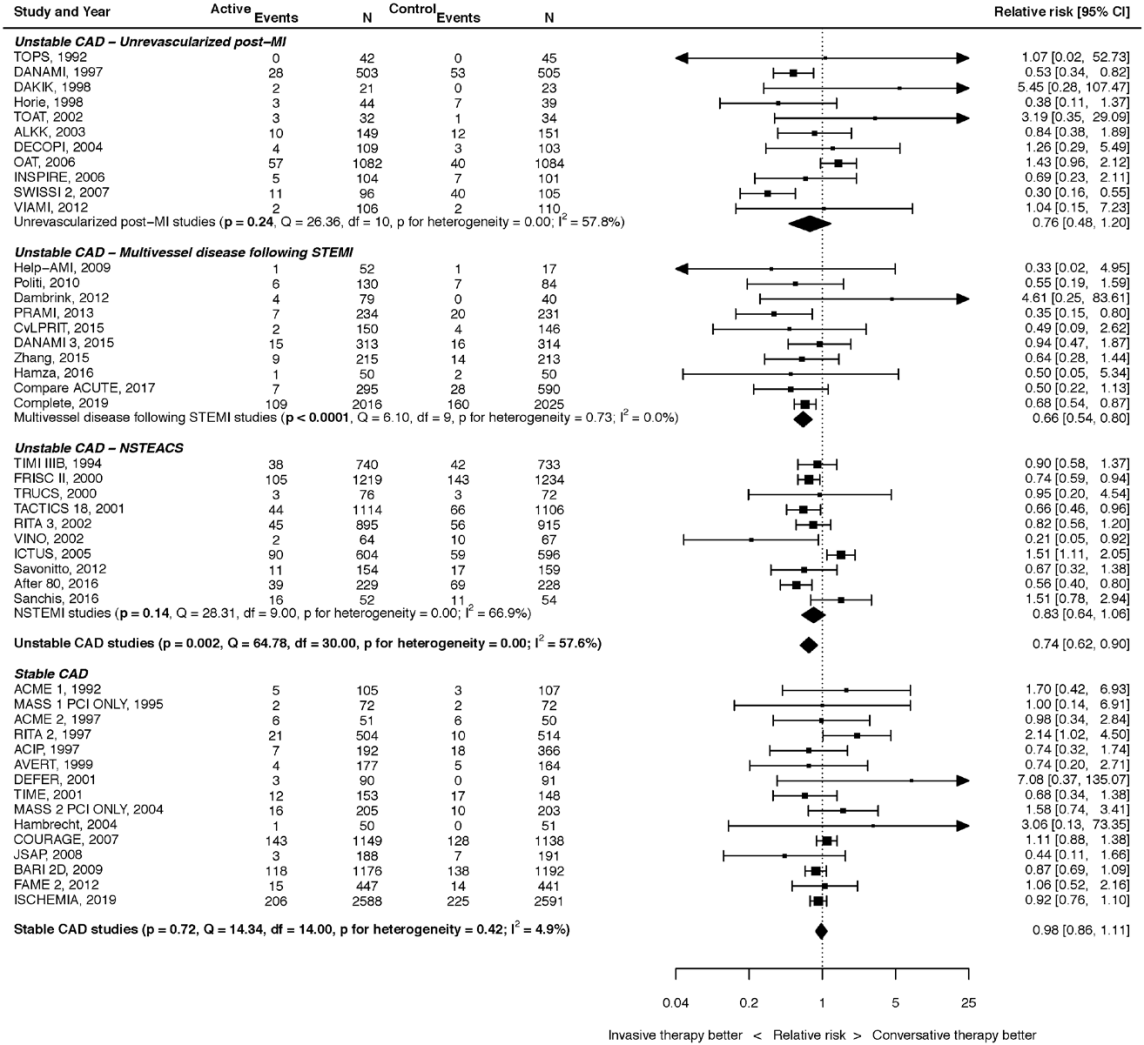
**The effect of percutaneous coronary intervention (PCI) on myocardial infarction (MI).** Results stratified into unstable coronary artery disease (CAD; unrevascularized post-MI,^10–20^ multivessel disease following ST-segment–elevation myocardial infarction [STEMI],^4,21–29^ non-ST segment–elevation acute coronary syndrome [NSTEACS]^30–39^) and stable CAD.^40–54^

A summary of the results for the effect of PCI on MI in CAD is shown in Figure 4.

For unrevascularized post-MI, the effect of PCI on MI was RR, 0.76 (95% CI, 0.48–1.20; *P*=0.24). There was significant heterogeneity (*I*^2^=57.8%). For multivessel disease following STEMI, there was a significant reduction in MI with PCI (RR, 0.66 [95% CI, 0.54–0.80]; *P*<0.001). There was no heterogeneity (*I*^2^=0.0%). For NSTEACS, the effect of PCI on MI was RR (0.83 [95% CI, 0.64–1.06]; *P*=0.136). There was significant heterogeneity (*I*^2^=66.9%). When considered together, PCI for unstable CAD led to a 26% reduction in MI (RR, 0.74 [95% CI, 0.62–0.90]; *P*=0.002). There was significant heterogeneity (*I*^2^=66.9%).

For stable CAD, there was no significant effect of PCI on MI, with RR, 0.96 (95% CI, 0.86–1.08; *P*=0.54). There was minimal heterogeneity (*I*^2^=2.0%).

### Sensitivity Analyses

A sensitivity analysis was performed for trials with longer-term follow-up. The results are shown in Figures V through VII in the Data Supplement. The results were broadly concordant with the primary analysis, although PCI was associated with a reduction in cardiac death at longer-term follow-up (Figure VI in the Data Supplement; RR, 0.81 [95% CI, 0.68–0.97]) and a nonsignificant reduction in MI (Figure VII in the Data Supplement; RR, 0.88 [95% CI, 0.73–1.06]; *P*=0.17) The *P* value for interaction for length of follow-up was nonsignificant (*P*=0.1013 for mortality; *P*=0.8772 for cardiovascular mortality; and *P*=0.9717 for MI)

An additional sensitivity analysis was performed using fixed effects for each of the main outcome measures, with results consistent with the primary analysis (Figures VIII through X in the Data Supplement).

Sensitivity analyses were also performed excluding trials in which CABG could be used as the revascularization strategy, the results of which are shown in Figures XI through XIII in the Data Supplement.

We performed sensitivity analyses excluding trials considered at high risk of bias, the results of which are shown in Figures XIV through XVI in the Data Supplement.

Finally, we also performed a sensitivity analysis in which each one of the trials in the main analysis has been removed in turn for the outcome of all-cause mortality. The result is shown in Figures XVII through LIX in the Data Supplement.

## Discussion

This analysis shows that for unstable CAD subsets, PCI reduces all-cause mortality by 16%, cardiovascular mortality by 31%, and MI by 26%. In contrast, PCI had no impact on these end points in patients with stable CAD. Our analysis incorporates results from 2 large, contemporary RCTs examining the role of PCI in different scenarios of CAD: the COMPLETE trial for multivessel disease in STEMI and the ISCHEMIA trial in stable CAD.

### Effect of PCI in Varying Clinical Syndromes

PCI is established to have a clear benefit in mortality over fibrinolysis, which itself almost halves the mortality of patients with STEMI. There is, therefore, no doubt over the survival benefit of primary PCI at the time of presentation with STEMI. The utility of PCI in other clinical syndromes, however, has been controversial. Outside the context of an ongoing STEMI lies not a simple unitary entity but a broad clinical spectrum.

As the years have passed and technology evolved, there has been an increasingly sophisticated categorization of patients between these groups. For example, in the first decades of angioplasty, a patient who had survived a STEMI to discharge and had subsequently been found to have a positive exercise test would be considered to have stable CAD,^16^ in much the same way as a patient with a several year-history of exertional angina. Modern practice, however, would be to consider the unrevascularized post-MI patient as requiring urgent angiography and revascularization if indicated. Doctors in current practice can certainly gain from trials of yesteryear but can do this best when trial patients are contextualized in the relevant part of the modern view of the clinical spectrum. This meta-analysis lays out this context simply and underlines the importance of placing patients in the correct categorization when deciding on whether they may benefit from PCI.

There is no evidence that PCI reduces mortality, cardiovascular mortality or MI in patients who have true stable CAD. Patients who have suffered an MI, however, do derive benefit from PCI. This grouping includes patients with NSTEACS, patients who have been discharged after an unrevascularized MI and also patients who have had PCI for the culprit artery in a STEMI, but who have residual coronary disease. It should be noted that the unrevascularized post-MI cohort is a group of trials from a time when STEMI was routinely managed with fibrinolysis and without angiography.

Our analysis underlines that these patients, who have had an unstable event, are distinct, and have specific therapeutic needs.

### The ISCHEMIA Trial

This analysis is the first to include the data from the ISCHEMIA trial,^54^ which was recently presented at the American Heart Association Scientific Sessions 2019 in Philadelphia. This trial randomized 5179 patients to invasive or conservative therapy. Revascularization was performed in 80% of patients randomized to invasive therapy, and PCI was the modality used in 74%. There was no difference in all-cause mortality, cardiovascular mortality or MI between the 2 groups. Procedural MI was increased with invasive therapy, while spontaneous MI was reduced with invasive therapy. The result of these 2 findings was that the net effect on MI is dependent on the timepoint at which it is measured. There is an early penalty in terms of MI with invasive therapy, but the curves cross at the 2-year timepoint and then continue to diverge in favor of invasive therapy. Although the overall effect of invasive therapy on MI was neutral, it is possible that if the curves continue to diverge then there would be a significant benefit of MI observed at longer-term follow-up. Clinicians may wish to counsel their patients regarding this when weighing options of invasive and conservative therapy.

### Clinical Implications

There has long been a belief that since heart disease is the leading cause of death worldwide,^55^ PCI might prevent deaths. However, within what we now define as stable CAD, there is no evidence of a net favorable effect on mortality, cardiovascular mortality, or MI. It should be remembered, however, that patients with left main CAD have not been randomized in these trials, and so if there is benefit in them, it would not be found by this meta-analysis.

Clinicians working in a modern environment should be careful to distinguish the generality of stable CAD from the other categories we describe, which we take together here and label unstable CAD, because the mortality impact of PCI differs between these 2 patient groups. This analysis can help provide clinicians with a framework when assessing patients with CAD in their clinical practice. If a patient has an acute coronary syndrome, then PCI can reasonably be offered on the grounds it will improve the clinical outcome of that patient. Similarly, if a patient has residual disease following PCI for a STEMI, that patient is also likely to have their prognosis improved by PCI to residual lesions. If a patient has had an MI but not been revascularized (less common in modern clinical practice), they also might derive prognostic benefit from PCI. For all other patients—that is, those who have truly stable CAD—PCI cannot reasonably be offered on prognostic grounds with the expectation it will reduce MI or prevent death. In this setting, PCI should be reserved for patients who experience angina refractory to medical therapy, in line with clinical guideline recommendations and recent blinded trial data.^56^

### Study Limitations

The ISCHEMIA trial has not yet been published in full. If the full published data differ from the presentation, we will update this analysis accordingly.

The ISCHEMIA trial, along with some others included in our analysis, is not truly a trial of PCI versus medical therapy; rather it is a trial of invasive therapy (angiography with a view to revascularization via PCI or CABG) versus conservative therapy.

We have performed sensitivity analyses excluding ISCHEMIA and other trials which included CABG as a mode of revascularization, or which generally randomized to invasive therapy rather than to PCI, and these plots are shown in the Data Supplement.

Definitions of categories of CAD change over time, in line with changing clinical practice. Previously,^16^ the unrevascularized post-MI state may have been considered stable CAD but this has now changed, and therefore, we cannot confidently predict how coronary disease will be categorized in the years to come. Nevertheless, we should always be sensitive to how studies have been grouped because this can influence the results.

MI is less solid in this respect because it is typically only tested for in patients with symptoms, and symptoms themselves are somewhat dependent on perception of patients’ clinical status (both by patient and by staff). We did not include angina as an end point because this is vulnerable to perception. As an example of this, the unblinded A Comparison of Angioplasty with Medical Therapy in the Treatment of Single-Vessel Coronary Artery Disease 1 trial^40^ found a 90-second increase in exercise time from plain balloon angioplasty, whereas the similar-sized but blinded ORBITA trial^57^ found only a 16-second increment from modern stenting.

Our primary end point was all-cause mortality as this is the most clinically relevant and bias-resistant end point. Our analysis is, therefore, focused on this mortality end point. Our study also uses MI as a secondary end point, as defined in each constituent trial. The definition of this end point varies substantially across trials. We only included prespecified end points, as these are more resistant to bias. We considered all MI together, so as not to introduce bias through selection (ie, we did not consider periprocedural MI separate from spontaneous MI). We also considered cardiovascular mortality as a secondary end point, although this is more vulnerable to bias than all-cause mortality because it requires adjudication. We did not use any other, nonprespecified end points so as not to introduce bias. Our results for cardiovascular mortality and MI show a higher degree of heterogeneity than our results for all-cause mortality, which is in part a reflection of these factors. Other potential sources of heterogeneity include differences in length of follow-up, pharmacotherapy, invasive therapy (balloons, bare metal stents, drug-eluting stents), and study populations. Our analysis includes trials from 1992 and from 2019, and in that period of time there has been significant advancement in both the in the pharmacological and invasive management of CAD. This is a further source of heterogeneity in such an analysis.

Most trials lacked adequate data, such as hazard ratios, which prevented a meta-analysis of survival data, and so this meta-analysis was performed using the relative risks provided by trials. Such effect sizes are typically more easily influenced by the time point chosen for analysis as they merely represent a single snapshot during follow-up. We provided a sensitivity analyses for longer-term follow-up of those trials which reported it, and the *P* value for interaction was nonsignificant when comparing timepoints.

Our study only addresses RCTs. They typically randomize only a minority of patients. However, this approach of focusing on RCTs is the best method of avoiding consistent bias in one direction or another from unmeasured confounders.

### Conclusions

PCI prevents death, cardiac death, and MI in patients with unstable CAD. For patients with stable CAD, PCI shows no evidence of an effect on any of these outcomes.

## Sources of Funding

We are grateful for infrastructural support from the Imperial College London and Imperial College Healthcare National Health Service Trust Biomedical Research Centers. D.P. Francis is supported by the British Heart Foundation (FS 04/079). J. Howard is supported by the Wellcome Trust (212183/Z/18/Z).

## Disclosures

R. Al-Lamee and S. Sen have received speaker’s honoraria from Philips Volcano. The other authors report no conflicts.

## Supplementary Material


