# Supplementary material for: Effects of Percutaneous Coronary Intervention on Death and Myocardial Infarction Stratified by Stable and Unstable Coronary Artery Disease: A Meta-Analysis of Randomized Controlled Trials
Source: Circ Cardiovasc Qual Outcomes. 2020 Feb 17;13(2):e006363. doi: 10.1161/CIRCOUTCOMES.119.006363 (PMC7034389; doi:10.1161/CIRCOUTCOMES.119.006363)

SUPPLEMENTAL MATERIAL

Online Appendix: Search strategy

OVID (Journals@Ovid Full Text, Embase, Ovid MEDLINE) Search Strategy

Delayed STEMI Culprit:

((Conservative OR medical therapy OR optimal medical therapy OR omt OR conservative or non invasive) AND (invasive OR delayed intervention OR angioplasty OR angiography OR ptca OR percutaneous coronary intervention OR percutaneous transluminal coronary angioplasty OR cardiac catheterization OR coronary angioplasty OR early coronary intervention OR delayed coronary intervention OR coronary invasive procedures OR eary pci OR interventional OR deferred coronary angioplasty OR late percutaneous coronary intervention OR late intervention OR delayed angioplasty OR timing OR unrevascularised) AND (ppci OR primary pci OR primary percutaneous coronary intervention OR pci OR stemi OR st segment elevation myocardial infarction OR ste or st elevation mi) AND (randomized controlled trial OR controlled clinical trial OR randomized OR placebo OR clinical trial OR randomly OR trial)).ti,ab.

Acute coronary syndrome:

((non st segment elevation myocardial infarction OR non st elevation myocardial infarction OR nstemi OR non st segment elevation mi OR non st elevation mi OR acute coronary syndrome OR acs OR unstable coronary artery disease OR unstable angina OR non q wave myocardial infarction OR

non q wave mi) AND (Percutaneous transluminal coronary angioplasty OR cardiac catheterisation OR bare metal stents OR stent OR cardiac stent OR drug eluting stent OR pci OR percutaneous coronary intervention OR ptca OR revascularisation OR invasive OR angioplasty OR angiography)
AND (conservative medical therapy OR medical therapy OR optimal medical therapy OR omt OR conservative OR placebo) AND (randomized controlled trial OR controlled clinical trial OR randomized OR placebo OR clinical trial OR randomly OR trial)).ti,ab.

Stable Angina:

((Percutaneous transluminal coronary angioplasty OR cardiac catheterisation OR bare metal stents OR stent OR cardiac stent OR drug eluting stent OR pci OR percutaneous coronary intervention OR ptca OR revascularisation) AND (stable angina OR stable angina pectoris OR angina OR coronary artery disease OR coronary disease OR cad OR stable coronary artery disease OR scad OR coronary stenosis) AND (randomized controlled trial OR controlled clinical trial OR randomized OR placebo OR clinical trial OR randomly OR trial) AND (conservative medical therapy OR medical therapy OR optimal medical therapy OR omt OR conservative OR placebo)).ti,ab.

STEMI Non Culprit

((Conservative OR medical therapy OR optimal medical therapy OR omt OR conservative or non invasive) AND (invasive OR delayed intervention OR
angioplasty OR angiography OR ptca OR percutaneous coronary intervention OR
percutaneous transluminal coronary angioplasty OR cardiac catheterization OR coronary angioplasty OR early coronary intervention OR delayed coronary intervention OR coronary invasive procedures OR eary pci OR interventional ORdeferred coronary angioplasty OR late percutaneous coronary intervention OR late intervention OR delayed angioplasty OR timing) AND (non-culprit OR

bystander OR culprit OR infarct-related OR non infarct related) AND (ppci OR primary pci OR primary percutaneous coronary intervention OR pci OR stemi OR st segment elevation myocardial infarction OR ste or st elevation mi) AND (randomized controlled trial OR controlled clinical trial OR randomized OR placebo OR clinical trial OR randomly OR trial)).ti,ab.

Cochrane Search Strategy

Delayed STEMI Culprit:

"STEMI":ti,ab,kw or "ST segment elevation" or "ST segment elevation myocardial infarction" (Word variations have been searched) AND "angioplasty":ti,ab,kw or "invasive" or "percutaneous coronary intervention" or "percutaneous transluminal angioplasty" or "interventional" or “unrevascularised” (Word variations have been searched) AND "conservative treatment":ti,ab,kw or "optimal medical therapy" or "conservative" (Word variations have been searched)

Acute Coronary Syndrome:

"non ST segment elevation":ti,ab,kw or "acute coronary syndrome" or "unstable angina pectoris" or "NSTEMI" or "ACS" (Word variations have been searched) AND "angioplasty":ti,ab,kw or "invasive" or "percutaneous coronary intervention" or "percutaneous transluminal angioplasty" or "interventional" (Word variations have been searched) AND "conservative treatment":ti,ab,kw or "optimal medical therapy" or "conservative" (Word variations have been searched)

Stable Angina:

"stable angina":ti,ab,kw or "stable angina pectoris" or "SCAD" (Word variations have been searched) AND "angioplasty":ti,ab,kw or "invasive" or "percutaneous coronary intervention" or "percutaneous transluminal angioplasty" or "interventional" (Word variations have been searched) AND "conservative treatment":ti,ab,kw or "optimal medical therapy" or "conservative" (Word variations have been searched)

STEMI Non Culprit

"STEMI":ti,ab,kw or "ST segment elevation" or "ST segment elevation myocardial infarction" (Word variations have been searched) AND "angioplasty":ti,ab,kw or "invasive" or "percutaneous coronary intervention" or "percutaneous transluminal angioplasty" or "interventional" or “non-culprit” or “bystander” or “culprit” or ‘infarct-related” or “non infarct related”)

(Word variations have been searched) AND "conservative treatment":ti,ab,kw or "optimal medical therapy" or "conservative" (Word variations have been searched)

PubMed Search Strategy

Delayed STEMI Culprit:

((((((("conservative"[All Fields] OR "medical therapy"[All Fields]) OR "optimal medical therapy"[All Fields]) OR "conservative"[All Fields]) OR "conservative treatment"[All Fields]) OR "non invasive treatment"[All Fields]) OR "timing"[All Fields]) AND (((((((((((((((("invasive"[All Fields] OR "delayed intervention"[All Fields]) OR "angioplasty"[All Fields]) OR "angiography"[All Fields]) OR "ptca"[All Fields]) OR "percutaneous coronary intervention"[All Fields]) OR "percutaneous transluminal coronary angioplasty"[All Fields]) OR "cardiac catheterization"[All Fields]) OR "coronary

angioplasty"[All Fields]) OR "early coronary intervention"[All Fields]) OR (delayed[All Fields] AND ("heart"[MeSH Terms] OR "heart"[All Fields] OR "coronary"[All Fields] OR “unrevascularised” [All Fields]) AND ("methods"[MeSH Terms] OR "methods"[All Fields] OR "intervention"[All Fields]))) OR "coronary invasive procedures"[All Fields]) OR "early pci"[All Fields]) OR "interventional"[All Fields]) OR (delayed[All Fields] AND ("methods"[MeSH Terms] OR "methods"[All Fields] OR "intervention"[All Fields]))) OR (deferred[All Fields] AND ("angioplasty, balloon, coronary"[MeSH Terms] OR ("angioplasty"[All Fields] AND "balloon"[All Fields] AND "coronary"[All Fields]) OR "coronary balloon angioplasty"[All Fields] OR ("coronary"[All Fields] AND "angioplasty"[All Fields]) OR "coronary angioplasty"[All Fields]))) OR (late[All Fields] AND ("percutaneous coronary intervention"[MeSH Terms] OR ("percutaneous"[All Fields] AND "coronary"[All Fields] AND "intervention"[All Fields]) OR "percutaneous coronary intervention"[All Fields]))) OR (late[All Fields] AND ("methods"[MeSH Terms] OR "methods"[All Fields] OR "intervention"[All Fields]))) OR (delayed[All Fields] AND ("angioplasty"[MeSH Terms] OR "angioplasty"[All Fields]))) OR timing[All Fields])) AND (((((("ppci"[All Fields] OR "primary pci"[All Fields]) OR "primary percutaneous coronary intervention"[All Fields]) OR "pci"[All Fields]) OR "stemi"[All Fields]) OR "st segment elevation mi"[All Fields]) OR "ste"[All Fields]) OR "st elevation mi"[All Fields] OR “unrevascularised”[All Fields])

Acute Coronary Syndrome:

(((((("conservative"[All Fields] OR "medical therapy"[All Fields]) OR "optimal medical therapy"[All Fields]) OR "conservative"[All Fields]) OR "conservative treatment"[All Fields]) OR "non invasive treatment"[All Fields]) OR "timing"[All Fields]) AND ((((((((((((((((("invasive"[All Fields] OR "delayed intervention"[All Fields]) OR "angioplasty"[All Fields]) OR "angiography"[All Fields]) OR "ptca"[All Fields]) OR "percutaneous coronary intervention"[All Fields]) OR "percutaneous transluminal coronary angioplasty"[All Fields]) OR "cardiac catheterization"[All Fields]) OR "coronary angioplasty"[All Fields]) OR "early coronary intervention"[All Fields]) OR (delayed[All Fields] AND

("heart"[MeSH Terms] OR "heart"[All Fields] OR "coronary"[All Fields]) AND ("methods"[MeSH Terms] OR "methods"[All Fields] OR "intervention"[All Fields]))) OR "coronary invasive procedures"[All Fields]) OR "early pci"[All Fields]) OR "interventional"[All Fields]) OR (delayed[All Fields] AND ("methods"[MeSH Terms] OR "methods"[All Fields] OR "intervention"[All Fields]))) OR (deferred[All Fields] AND ("angioplasty, balloon, coronary"[MeSH Terms] OR ("angioplasty"[All Fields] AND "balloon"[All Fields] AND "coronary"[All Fields]) OR "coronary balloon angioplasty"[All Fields] OR ("coronary"[All Fields] AND "angioplasty"[All Fields]) OR "coronary angioplasty"[All Fields]))) OR (late[All Fields] AND ("percutaneous coronary intervention"[MeSH Terms] OR ("percutaneous"[All Fields] AND "coronary"[All Fields] AND "intervention"[All Fields]) OR "percutaneous coronary intervention"[All Fields]))) OR (late[All Fields] AND ("methods"[MeSH Terms] OR "methods"[All Fields] OR "intervention"[All Fields]))) OR (delayed[All Fields] AND ("angioplasty"[MeSH Terms] OR "angioplasty"[All Fields]))) OR timing[All Fields])) AND (((((("ppci"[All Fields] OR "primary pci"[All Fields]) OR "primary percutaneous coronary intervention"[All Fields]) OR "pci"[All Fields]) OR "stemi"[All Fields]) OR "st segment elevation mi"[All Fields]) OR "ste"[All Fields]) OR "st elevation mi"[All Fields])

Stable Angina:

((((((((("percutaneous transluminal coronary angioplasty"[All Fields] OR "cardiac catheterisation"[All Fields]) OR "bare metal stents"[All Fields]) OR "stent"[All Fields]) OR "cardiac stent"[All Fields]) OR "drug eluting stent"[All Fields]) OR "pci"[All Fields]) OR "percutaneous coronary intervention"[All Fields]) OR "ptca"[All Fields]) OR "revascularisation"[All Fields]) AND (((((("stable angina"[All Fields] OR "stable angina pectoris"[All Fields]) OR "angina"[All Fields]) OR "coronary artery disease"[All Fields]) OR "coronary disease"[All Fields]) OR "stable coronary artery disease"[All Fields]) OR "scad"[All Fields]) OR "coronary stenosis"[All Fields])) AND ((((("conservative medical therapy"[All Fields] OR "medical therapy"[All Fields]) OR "optimal medical therapy"[All Fields]) OR

"omt"[All Fields]) OR "conservative"[All Fields]) OR "placebo"[All Fields])) AND (("randomised controlled trial"[All Fields] OR "randomized controlled trial"[All Fields]) OR "rct"[All Fields])

STEMI Non Culprit

((((((("conservative"[All Fields] OR "medical therapy"[All Fields]) OR "optimal medical therapy"[All Fields]) OR "conservative"[All Fields]) OR "conservative treatment"[All Fields]) OR "non invasive treatment"[All Fields]) OR "timing"[All Fields]) AND (((((((((((((((((("invasive"[All Fields] OR "delayed intervention"[All Fields]) OR "angioplasty"[All Fields]) OR "angiography"[All Fields]) OR "ptca"[All Fields]) OR "percutaneous coronary intervention"[All Fields]) OR "percutaneous transluminal coronary angioplasty"[All Fields]) OR "cardiac catheterization"[All Fields]) OR "coronary angioplasty"[All Fields]) OR "non-culprit"[All Fields]) OR (“culprit”[All Fields] OR bystander” OR “infarct related” OR “non infarct related”AND ("heart"[MeSH Terms] OR "heart"[All Fields] OR "coronary"[All Fields]) AND ("methods"[MeSH Terms] OR "methods"[All Fields] OR "intervention"[All Fields]))) OR "coronary invasive procedures"[All Fields]) OR "early pci"[All Fields]) OR "interventional"[All Fields]) OR (delayed[All Fields] AND ("methods"[MeSH Terms] OR "methods"[All Fields] OR "intervention"[All Fields]))) OR (deferred[All Fields] AND ("angioplasty, balloon, coronary"[MeSH Terms] OR ("angioplasty"[All Fields] AND "balloon"[All Fields] AND "coronary"[All Fields]) OR "coronary balloon angioplasty"[All Fields] OR ("coronary"[All Fields] AND "angioplasty"[All Fields]) OR "coronary angioplasty"[All Fields]))) OR (late[All Fields] AND ("percutaneous coronary intervention"[MeSH Terms] OR ("percutaneous"[All Fields] AND "coronary"[All Fields] AND "intervention"[All Fields]) OR "percutaneous coronary intervention"[All Fields]))) OR (late[All Fields] AND ("methods"[MeSH Terms] OR "methods"[All Fields] OR "intervention"[All Fields]))) OR (delayed[All Fields] AND ("angioplasty"[MeSH Terms] OR "angioplasty"[All Fields]))) OR timing[All Fields])) AND ((((((("ppci"[All Fields] OR "primary pci"[All Fields]) OR "primary percutaneous coronary intervention"[All Fields]) OR "pci"[All Fields]) OR

"stemi"[All Fields]) OR "st segment elevation mi"[All Fields]) OR "ste"[All Fields]) OR "st elevation mi"[All Fields])

Online Table 1. Risk of Bias Assessment of Included Trials

Trial	*Random sequence generation*	*Allocation concealment*	*Blinding of participants & personnel*	*Blinding of outcome assessment*	*Incomplete outcome data*	*Overall Quality*
**ALKK**	Low risk	Low risk	High risk	Low risk	Low risk	Low risk
**SWISSI 2**	Low risk	Low risk	High risk	Low risk	Low risk	Low risk
**OAT (2006 & 2011)**	Low risk	Low risk	High risk	Low risk	Low risk	Low risk
**VIAMI (2012 & 2015)**	Unclear	Unclear	High risk	Low risk	Low risk	High risk
**DECOPI**	Unclear	Unclear	High risk	Low risk	Low risk	Low risk
**TOAT**	Low risk	Unclear	High risk	Low risk	Low risk	Low risk
**TOPS**	Low risk	Low risk	High risk	Low risk	Low risk	Low risk
**Horie**	Low risk	Low risk	High risk	Low risk	Low risk	Low risk
**INSPIRE**	Unclear	Unclear	Unclear	Low risk	Low risk	Low risk
**DANAMI**	Unclear	Unclear	High risk	Low risk	Low risk	Low risk
**Dakik**	Unclear	Unclear	High risk	Low risk	Unclear	Unclear
**TIMI IIIB (1994& 199**	Unclear	Low risk	High risk	Unclear	Low risk	Low risk
**FRISC II (2000 & 201**	Low risk	Low risk	High risk	High risk	Low risk	Low risk
**TRUCS**	Unclear	Unclear	High risk	Unclear	High risk	High risk
**TACTICS 18**	Unclear	Low risk	High risk	Unclear	Low risk	Low risk
**RITA 3 (2002 & 2015**	Unclear	Low risk	High risk	High risk	Low risk	Low risk
**VINO**	Low risk	Low risk	High risk	High risk	Low risk	Low risk
**ICTUS (2005 &2017)**	Low risk	Low risk	High risk	Low risk	Low risk	Low risk
**Savonitto**	Low risk	Low risk	High risk	Low risk	Low risk	Low risk
**After 80**	Unclear	Low risk	High risk	Unclear	Low risk	Low risk
**Sanchis**	Low risk	Low risk	High risk	Unclear	Low risk	Low risk
**ACME 1 (1992 & 199**	Unclear	Unclear	High risk	Unclear	Low risk	High risk
**ACME 2 (1997)**	Unclear	Unclear	High risk	Unclear	Low risk	High risk
**AVERT**	Unclear	Unclear	High risk	Low risk	Low risk	High risk
**RITA 2, (1997 &2003**	Unclear	Low risk	High risk	Low risk	Low risk	Low risk
**MASS 1 (1995 &1999**	Unclear	Unclear	High risk	Unclear	Low risk	High risk
**DEFER (2001 &2015)**	Unclear	Unclear	High risk	Unclear	Low risk	Low risk
**MASS 2 (2004 & 201**	Unclear	Unclear	High risk	Unclear	Low risk	Low risk
**COURAGE(2007 & 20**	Unclear	Unclear	High risk	Low risk	Low risk	Low risk
**JSAP**	Low risk	Low risk	High risk	Low risk	Low risk	Low risk
**BARI 2D**	Unclear	Unclear	High risk	Low risk	Low risk	High risk
**FAME 2 (2012 & 201**	Low risk	Unclear	High risk	Low risk	Low risk	Low risk
**TIME (2001& 2004)**	Low risk	Unclear	High risk	Unclear	Low risk	Low risk
**ACIP**	Unclear	Unclear	High risk	Unclear	Low risk	High risk
**Hambrecht**	Unclear	Unclear	High risk	Unclear	Low risk	High risk
**ISCHEMIA**	Low risk	Low risk	High risk	Low risk	Unclear	Low risk
**Complete**	Low risk	Low risk	Unclear	Low risk	Low risk	Low risk
**Compare ACUTE**	Low risk	Low risk	Unclear	Low risk	Low risk	Low risk
**CvLPRIT**	Low risk	Low risk	High risk	Low risk	High risk	Low risk
**DANAMI 3**	Low risk	Unclear	High risk	Low risk	Low risk	Low risk
**PRAMI**	Low risk	Unclear	High risk	Low risk	High risk	High risk
**Hamza**	Unclear	Unclear	Unclear	Unclear	Low risk	High risk
**Dambrink**	Low risk	Unclear	Unclear	Unclear	Low risk	High risk
**Politi**	Low risk	Unclear	Unclear	Unclear	Unclear	High risk
**Help-AMI**	Unclear	Unclear	Unclear	Unclear	Unclear	High risk
**Zhang**	Unclear	Unclear	Unclear	Unclear	Unclear	High risk

Online Figure 1. Funnel plot for publication bias for the outcome of all-cause mortality for unrevascularized post-MI

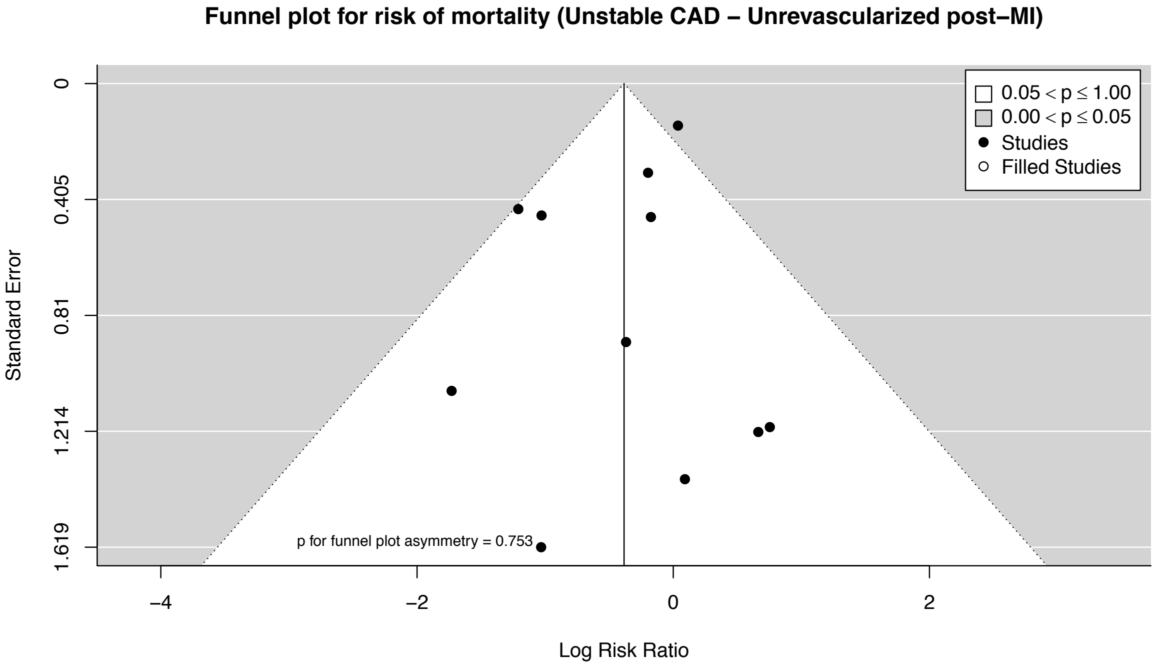

Online Figure 2. Funnel plot for publication bias for the outcome of all-cause mortality for multivessel disease following STEMI

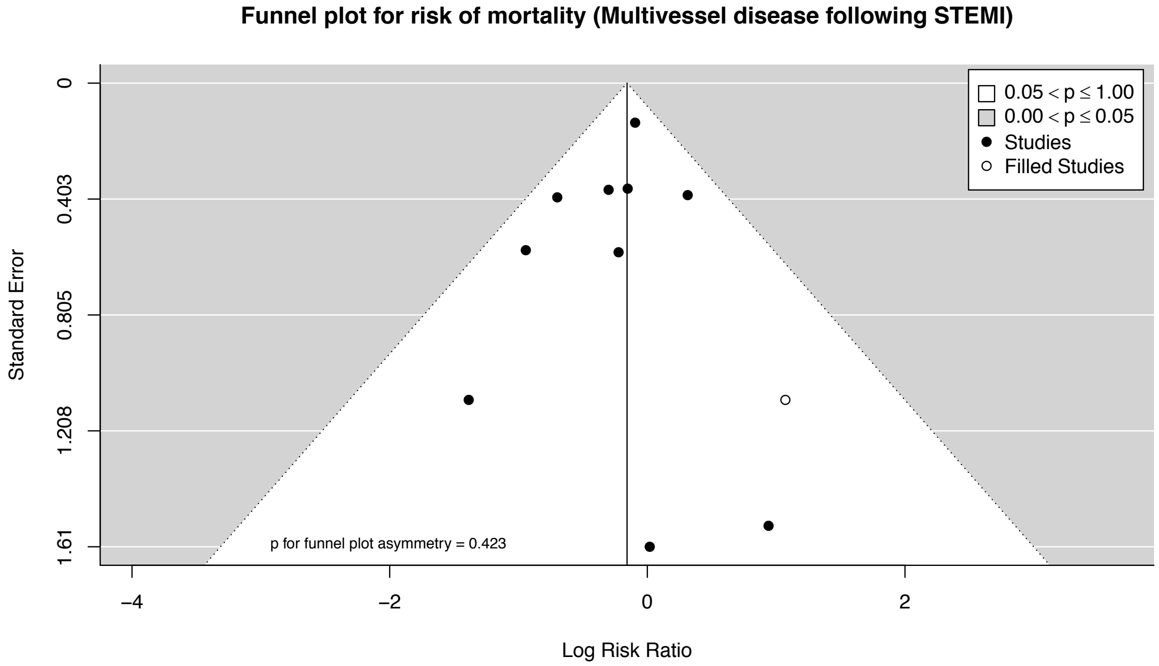

Online Figure 3. Funnel plot for publication bias for the outcome of all-cause mortality for NSTEACS

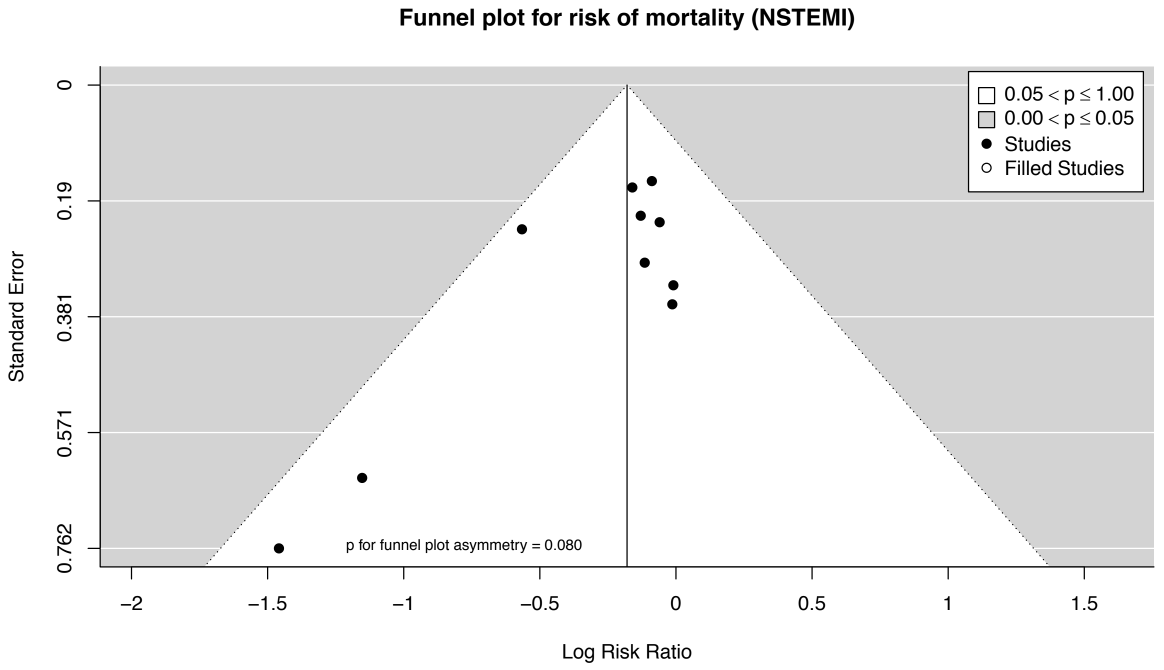

Online Figure 4. Funnel plot for publication bias for the outcome of all-cause mortality for stable CAD

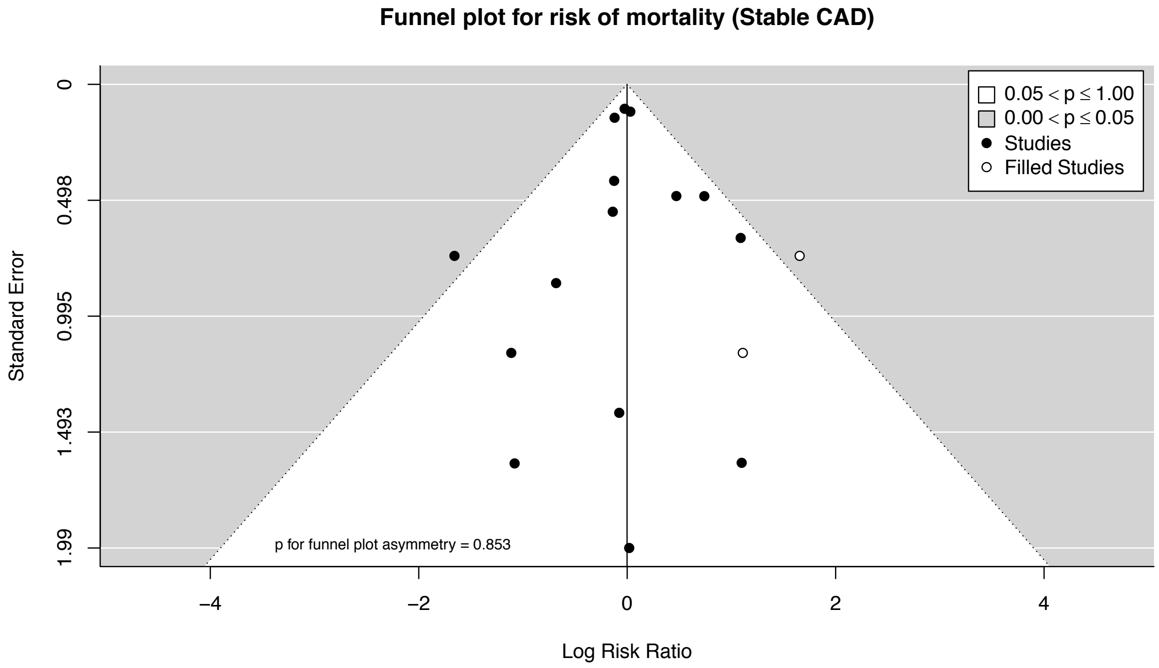

Online Figure 5. Sensitivity analysis for longer-term follow-up for all-cause mortality

Study and Year	Active Events	N	Control Events	N	Relative risk [95% CI]
Unstable CAD – Unrevascularized post–MI					
TOPS, 1992	0	42	1	45	0.36 [0.01, 8.52]
DANAMI, 1997	18	503	22	505	0.82 [0.45, 1.51]
DAKIK, 1998	1	21	1	23	1.10 [0.07, 16.43]
Horie, 1998	1	44	5	39	0.18 [0.02, 1.45]
TOAT, 2002	2	32	1	34	2.13 [0.20, 22.31]
ALKK, 2003	6	149	17	151	0.36 [0.15, 0.88]
DECOPI, 2004	8	109	9	103	0.84 [0.34, 2.09]
INSPIRE, 2006	2	104	1	101	1.94 [0.18, 21.09]
SWISSI 2, 2007	6	96	22	105	0.30 [0.13, 0.70]
OAT, 2011	150	1101	153	1100	0.98 [0.79, 1.21]
VIAMI, 2015	9	106	9	110	1.04 [0.43, 2.51]
Unrevascularized post–MI studies (**p = 0.07**, Q = 14.63, df = 10, p for heterogeneity = 0.15; I^2 = 37.6%)					0.72 [0.50, 1.03]
Unstable CAD – Multivessel disease following STEMI					
Help–AMI, 2009	1	52	0	17	1.02 [0.04, 23.91]
Politi, 2010	10	130	13	84	0.50 [0.23, 1.08]
Dambrink, 2012	2	79	0	40	2.56 [0.13, 52.14]
PRAMI, 2013	12	234	16	231	0.74 [0.36, 1.53]
CvLPRIT, 2015	4	150	10	146	0.39 [0.12, 1.21]
DANAMI 3, 2015	15	313	11	314	1.37 [0.64, 2.93]
Zhang, 2015	13	215	15	213	0.86 [0.42, 1.76]
Hamza, 2016	1	50	4	50	0.25 [0.03, 2.16]
Compare ACUTE, 2017	4	295	10	590	0.80 [0.25, 2.53]
Complete, 2019	96	2016	106	2025	0.91 [0.70, 1.19]
Multivessel disease following STEMI studies (**p = 0.11**, Q = 7.30, df = 9, p for heterogeneity = 0.61; I^2 = 0.0%)					0.84 [0.69, 1.04]
Unstable CAD – NSTEACS					
TIMI IIIB, 1995	30	740	32	733	0.93 [0.57, 1.51]
TRUCS, 2000	3	76	9	72	0.32 [0.09, 1.12]
TACTICS 18, 2001	37	1114	39	1106	0.94 [0.61, 1.47]
VINO, 2002	2	64	9	67	0.23 [0.05, 1.04]
Savonitto, 2012	19	154	22	159	0.89 [0.50, 1.58]
RITA 3, 10 year, 2015	225	895	232	915	0.99 [0.85, 1.16]
FRISC II, 2016	473	1222	483	1235	0.99 [0.90, 1.09]
After 80, 2016	57	229	62	228	0.92 [0.67, 1.25]
Sanchis, 2016	22	52	26	54	0.88 [0.58, 1.34]
ICTUS, 10 year, 2017	156	604	138	596	1.12 [0.91, 1.36]
NSTEMI studies (**p = 0.71**, Q = 8.91, df = 9.00, p for heterogeneity = 0.45; I^2 = 0.0%)					0.99 [0.92, 1.06]
**Unstable CAD studies (p = 0.181, Q = 33.95, df = 30.00, p for heterogeneity = 0.28; I^2 = 0.0%)**					0.96 [0.90, 1.02]
Stable CAD					
ACIP, 1997	2	192	20	366	0.19 [0.05, 0.81]
ACME 1, 1998	5	105	7	107	0.73 [0.24, 2.22]
AVERT, 1999	1	177	1	164	0.93 [0.06, 14.69]
MASS 1, 5 year, 1999	6	72	6	72	1.00 [0.34, 2.95]
RITA 2, 7 year, 2003	43	504	43	514	1.02 [0.68, 1.53]
TIME, 2004	43	153	40	148	1.04 [0.72, 1.50]
Hambrecht, 2004	0	50	0	51	1.02 [0.02, 50.41]
JSAP, 2008	6	188	7	191	0.87 [0.30, 2.54]
BARI 2D, 2009	155	1176	161	1192	0.98 [0.79, 1.20]
MASS 2, 10 year, 2010	100	408	63	203	0.79 [0.60, 1.03]
DEFER, 15 year, 2015	28	90	30	91	0.94 [0.62, 1.44]
COURAGE, 15 year, 2015	284	1149	277	1138	1.02 [0.88, 1.17]
FAME 2, 5 years, 2018	23	447	23	441	0.99 [0.56, 1.73]
ISCHEMIA, 2019	140	2588	136	2591	1.03 [0.82, 1.30]
**Stable CAD studies (p = 0.52, Q = 8.34, df = 13.00, p for heterogeneity = 0.82; I^2 = 0.0%)**					0.97 [0.89, 1.06]

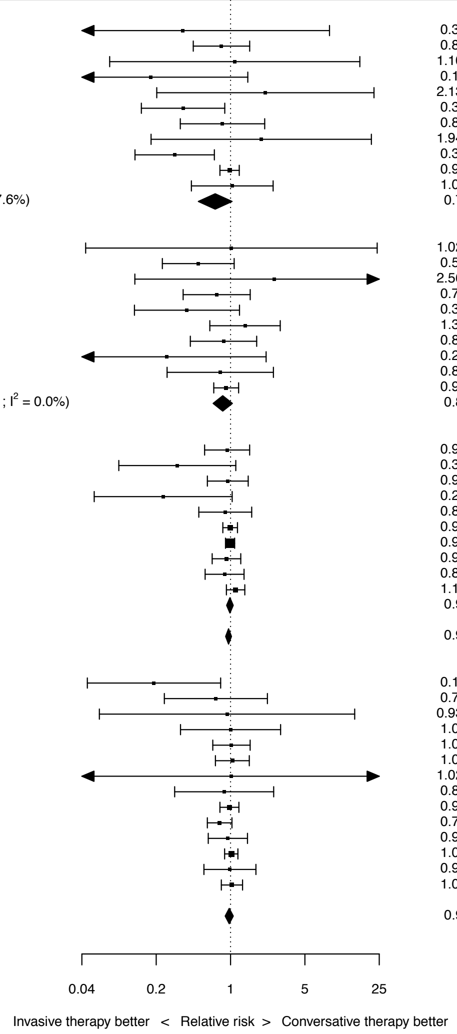

Online Figure 6. Sensitivity analysis for longer-term follow-up for cardiovascular mortality

Study and Year	Active Events	Active N	Control Events	Control N	Relative risk [95% CI]
Unstable CAD – Unrevascularized post–MI					
TOPS, 1992	0	42	0	45	1.07 [0.02, 52.73]
DAKIK, 1998	1	21	1	23	1.10 [0.07, 16.43]
Horie, 1998	1	44	4	39	0.22 [0.03, 1.90]
ALKK, 2003	4	149	14	151	0.29 [0.10, 0.86]
DECOPI, 2004	6	109	7	103	0.81 [0.28, 2.33]
INSPIRE, 2006	2	104	1	101	1.94 [0.18, 21.09]
SWISSI 2, 2007	3	96	22	105	0.15 [0.05, 0.48]
OAT, 2011	77	1101	81	1100	0.95 [0.70, 1.28]
Unrevascularized post–MI studies (**p = 0.07**, Q = 14.43, df = 7, p for heterogeneity = 0.04; I^2 = 52.6%)					0.54 [0.27, 1.05]
Unstable CAD – Multivessel disease following STEMI					
Politi, 2010	6	130	10	84	0.39 [0.15, 1.03]
PRAMI, 2013	4	234	10	231	0.39 [0.13, 1.24]
CvLPRIT, 2015	2	150	7	146	0.28 [0.06, 1.32]
DANAMI 3, 2015	5	313	9	314	0.56 [0.19, 1.64]
Zhang, 2015	11	215	14	213	0.78 [0.36, 1.68]
Compare ACUTE, 2017	3	295	6	590	1.00 [0.25, 3.97]
Complete, 2019	59	2016	64	2025	0.93 [0.65, 1.31]
Multivessel disease following STEMI studies (**p = 0.04**, Q = 6.40, df = 6, p for heterogeneity = 0.38; I^2 = 21.8%)					0.68 [0.47, 0.98]
Unstable CAD – NSTEACS					
Savonitto, 2012	16	154	17	159	0.97 [0.51, 1.85]
RITA 3, 10 year, 2015	135	895	147	915	0.94 [0.76, 1.16]
FRISC II, 2016	175	1222	198	1235	0.89 [0.74, 1.08]
ICTUS, 10 year, 2017	97	604	85	596	1.13 [0.86, 1.47]
NSTEMI studies (**p = 0.47**, Q = 1.96, df = 3.00, p for heterogeneity = 0.58; I^2 = 0.0%)					0.96 [0.85, 1.08]
**Unstable CAD studies (p = 0.040, Q = 26.28, df = 18.00, p for heterogeneity = 0.09; I^2 = 0.0%)**					0.90 [0.81, 0.99]
Stable CAD					
AVERT, 1999	1	177	1	164	0.93 [0.06, 14.69]
MASS 1, 5 year, 1999	3	72	2	72	1.50 [0.26, 8.71]
RITA 2, 7 year, 2003	20	504	24	514	0.85 [0.48, 1.52]
TIME, 2004	32	153	33	148	0.94 [0.61, 1.44]
Hambrecht, 2004	0	50	0	51	1.02 [0.02, 50.41]
JSAP, 2008	2	188	3	191	0.68 [0.11, 4.01]
MASS 2, 10 year, 2010	51	408	42	203	0.60 [0.42, 0.88]
DEFER, 15 year, 2015	4	90	5	91	0.81 [0.22, 2.92]
FAME 2, 5 years, 2018	11	447	7	441	1.55 [0.61, 3.96]
ISCHEMIA, 2019	89	2588	108	2591	0.83 [0.63, 1.09]
**Stable CAD studies (p = 0.02, Q = 5.25, df = 9.00, p for heterogeneity = 0.81; I^2 = 0.0%)**					0.81 [0.68, 0.97]

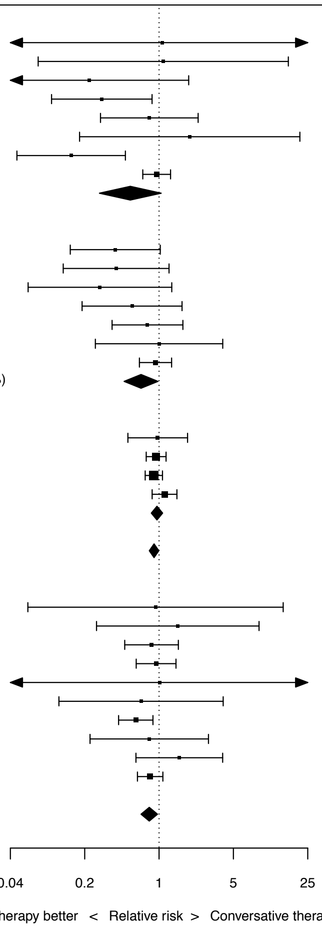

Online Figure 7. Sensitivity analysis for longer-term follow-up for myocardial infarction

Study and Year	Active Events	Active N	Control Events	Control N	Relative risk [95% CI]
Unstable CAD – Unrevascularized post–MI					
TOPS, 1992	0	42	0	45	1.07 [0.02, 52.73]
DANAMI, 1997	28	503	53	505	0.53 [0.34, 0.82]
DAKIK, 1998	2	21	0	23	5.45 [0.28, 107.47]
Horie, 1998	3	44	7	39	0.38 [0.11, 1.37]
TOAT, 2002	3	32	1	34	3.19 [0.35, 29.09]
ALKK, 2003	10	149	12	151	0.84 [0.38, 1.89]
DECOPI, 2004	4	109	3	103	1.26 [0.29, 5.49]
INSPIRE, 2006	5	104	7	101	0.69 [0.23, 2.11]
SWISSI 2, 2007	11	96	40	105	0.30 [0.16, 0.55]
OAT, 2011	75	1101	61	1100	1.23 [0.89, 1.70]
VIAMI, 2015	8	106	12	110	0.69 [0.29, 1.62]
Unrevascularized post–MI studies (**p = 0.11**, Q = 24.78, df = 10, p for heterogeneity = 0.01; I^2 = 54.5%)					0.72 [0.48, 1.07]
Unstable CAD – Multivessel disease following STEMI					
Help–AMI, 2009	1	52	1	17	0.33 [0.02, 4.95]
Politi, 2010	6	130	7	84	0.55 [0.19, 1.59]
Dambrink, 2012	4	79	0	40	4.61 [0.25, 83.61]
PRAMI, 2013	7	234	20	231	0.35 [0.15, 0.80]
CvLPRIT, 2015	2	150	4	146	0.49 [0.09, 2.62]
DANAMI 3, 2015	15	313	16	314	0.94 [0.47, 1.87]
Zhang, 2015	9	215	14	213	0.64 [0.28, 1.44]
Hamza, 2016	1	50	2	50	0.50 [0.05, 5.34]
Compare ACUTE, 2017	7	295	28	590	0.50 [0.22, 1.13]
Complete, 2019	109	2016	160	2025	0.68 [0.54, 0.87]
Multivessel disease following STEMI studies (**p < 0.0001**, Q = 6.10, df = 9, p for heterogeneity = 0.73; I^2 = 0.0%)					0.66 [0.54, 0.80]
Unstable CAD – NSTEACS					
TIMI IIIB, 1995	61	740	68	733	0.89 [0.64, 1.24]
TRUCS, 2000	3	76	3	72	0.95 [0.20, 4.54]
TACTICS 18, 2001	44	1114	66	1106	0.66 [0.46, 0.96]
VINO, 2002	2	64	10	67	0.21 [0.05, 0.92]
Savonitto, 2012	11	154	17	159	0.67 [0.32, 1.38]
FRISC II, 2016	455	1222	557	1235	0.83 [0.75, 0.91]
After 80, 2016	39	229	69	228	0.56 [0.40, 0.80]
Sanchis, 2016	16	52	11	54	1.51 [0.78, 2.94]
ICTUS, 10 year, 2017	106	604	84	596	1.25 [0.96, 1.62]
NSTEMI studies (**p = 0.11**, Q = 22.30, df = 8.00, p for heterogeneity = 0.00; I^2 = 70.6%)					0.82 [0.65, 1.04]
**Unstable CAD studies (p = 0.001, Q = 58.47, df = 29.00, p for heterogeneity = 0.00; I^2 = 61.7%)**					0.73 [0.62, 0.87]
Stable CAD					
ACIP, 1997	7	192	18	366	0.74 [0.32, 1.74]
ACME 1, 1998	10	105	7	107	1.46 [0.58, 3.68]
AVERT, 1999	4	177	5	164	0.74 [0.20, 2.71]
MASS 1, 5 year, 1999	5	72	3	72	1.67 [0.41, 6.72]
RITA 2, 7 year, 2003	32	504	23	514	1.42 [0.84, 2.39]
TIME, 2004	18	153	18	148	0.97 [0.52, 1.79]
Hambrecht, 2004	1	50	0	51	3.06 [0.13, 73.35]
JSAP, 2008	3	188	7	191	0.44 [0.11, 1.66]
BARI 2D, 2009	118	1176	138	1192	0.87 [0.69, 1.09]
MASS 2, 10 year, 2010	48	408	42	203	0.57 [0.39, 0.83]
DEFER, 15 year, 2015	9	90	2	91	4.55 [1.01, 20.48]
FAME 2, 5 years, 2018	36	447	53	441	0.67 [0.45, 1.00]
ISCHEMIA, 2019	206	2588	225	2591	0.92 [0.76, 1.10]
**Stable CAD studies (p = 0.17, Q = 18.77, df = 12.00, p for heterogeneity = 0.09; I^2 = 34.6%)**					0.88 [0.73, 1.06]

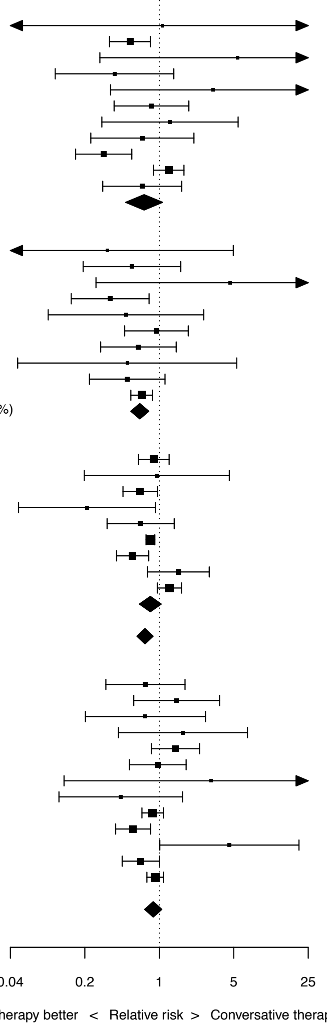

Online Figure 8. Sensitivity analysis with fixed effects for all-cause mortality

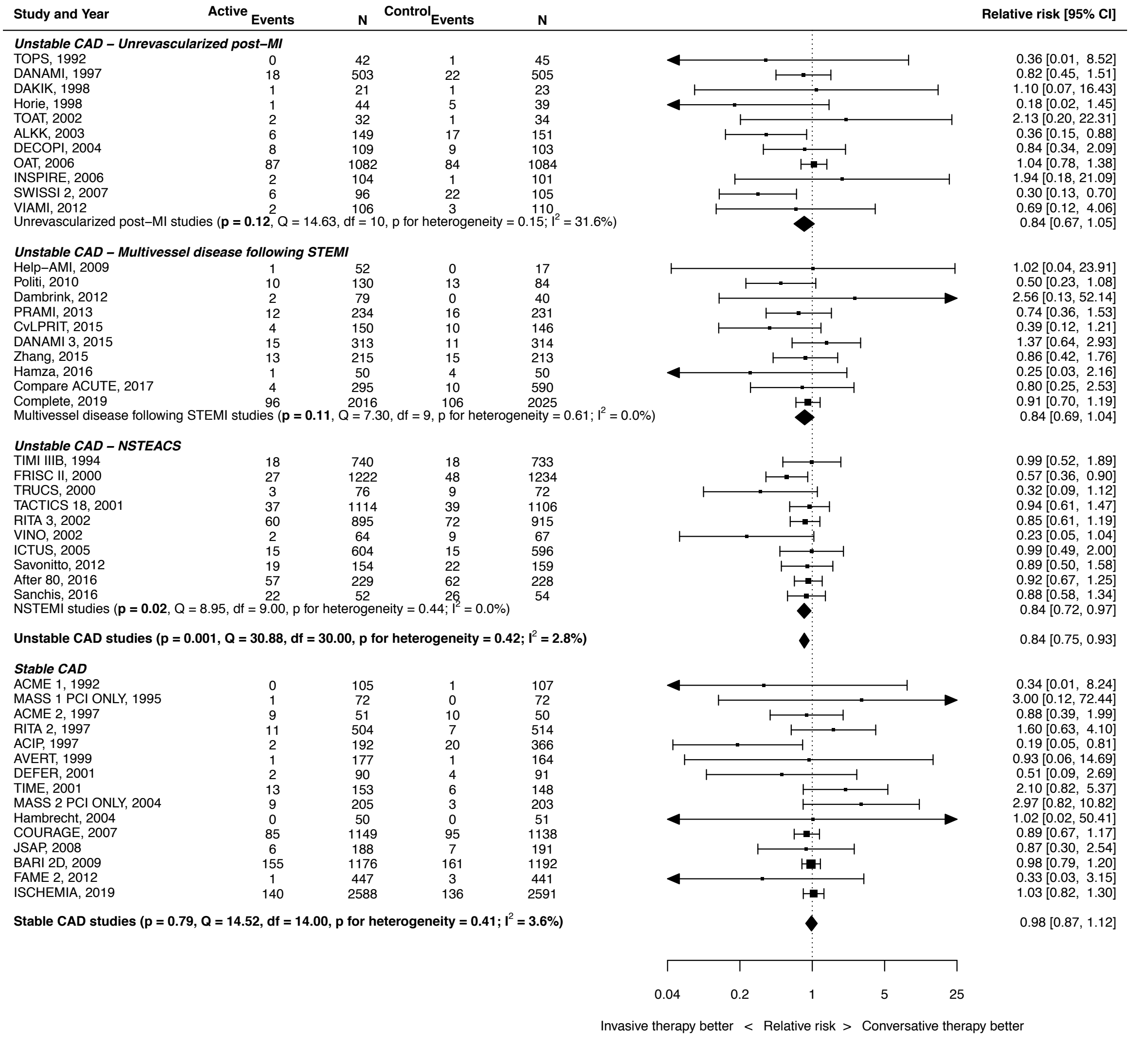

Online Figure 9. Sensitivity analysis with fixed effects for cardiac death

Study and Year	Active Events	Active N	Control Events	Control N	Relative risk [95% CI]
Unstable CAD – Unrevascularized post–MI					
TOPS, 1992	0	42	0	45	1.07 [0.02, 52.73]
DAKIK, 1998	1	21	1	23	1.10 [0.07, 16.43]
Horie, 1998	1	44	4	39	0.22 [0.03, 1.90]
ALKK, 2003	4	149	14	151	0.29 [0.10, 0.86]
DECOPI, 2004	6	109	7	103	0.81 [0.28, 2.33]
OAT, 2006	58	1082	52	1084	1.12 [0.78, 1.61]
INSPIRE, 2006	2	104	1	101	1.94 [0.18, 21.09]
SWISSI 2, 2007	3	96	22	105	0.15 [0.05, 0.48]
Unrevascularized post–MI studies (**p = 0.23**, Q = 16.36, df = 7, p for heterogeneity = 0.02; I^2 = 57.2%)					0.83 [0.61, 1.13]
Unstable CAD – Multivessel disease following STEMI					
Politi, 2010	6	130	10	84	0.39 [0.15, 1.03]
PRAMI, 2013	4	234	10	231	0.39 [0.13, 1.24]
CvLPRIT, 2015	2	150	7	146	0.28 [0.06, 1.32]
DANAMI 3, 2015	5	313	9	314	0.56 [0.19, 1.64]
Zhang, 2015	11	215	14	213	0.78 [0.36, 1.68]
Compare ACUTE, 2017	3	295	6	590	1.00 [0.25, 3.97]
Complete, 2019	59	2016	64	2025	0.93 [0.65, 1.31]
Multivessel disease following STEMI studies (**p = 0.04**, Q = 6.40, df = 6, p for heterogeneity = 0.38; I^2 = 6.3%)					0.76 [0.58, 0.99]
Unstable CAD – NSTEACS					
RITA 3, 2002	55	895	74	915	0.76 [0.54, 1.06]
Savonitto, 2012	16	154	17	159	0.97 [0.51, 1.85]
NSTEMI studies (**p = 0.14**, Q = 0.44, df = 1.00, p for heterogeneity = 0.51; I^2 = 0.0%)					0.80 [0.59, 1.08]
**Unstable CAD studies (p = 0.006, Q = 23.41, df = 16.00, p for heterogeneity = 0.10; I^2 = 31.7%)**					0.79 [0.67, 0.94]
Stable CAD					
ACME 1, 1992	0	105	1	107	0.34 [0.01, 8.24]
RITA 2, 1997	5	504	3	514	1.70 [0.41, 7.07]
AVERT, 1999	1	177	1	164	0.93 [0.06, 14.69]
DEFER, 2001	1	90	2	90	0.50 [0.05, 5.42]
MASS 2 PCI ONLY, 2004	9	205	3	203	2.97 [0.82, 10.82]
Hambrecht, 2004	0	50	0	51	1.02 [0.02, 50.41]
COURAGE, 2007	23	1149	25	1138	0.91 [0.52, 1.60]
JSAP, 2008	2	188	3	191	0.68 [0.11, 4.01]
FAME 2, 2012	1	447	1	441	0.99 [0.06, 15.72]
ISCHEMIA, 2019	89	2588	108	2591	0.83 [0.63, 1.09]
**Stable CAD studies (p = 0.30, Q = 5.10, df = 9.00, p for heterogeneity = 0.83; I^2 = 0.0%)**					0.88 [0.70, 1.12]

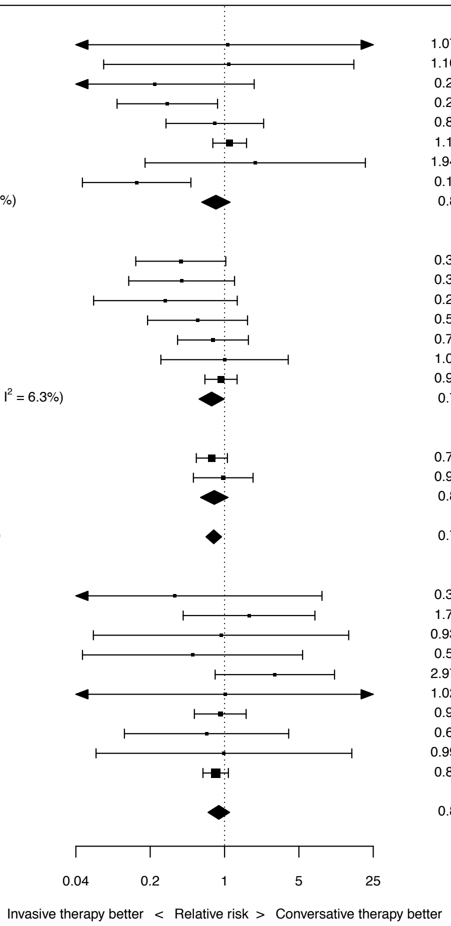

Online Figure 10. Sensitivity analysis with fixed effects for myocardial infarction

Study and Year	Active Events	Active N	Control Events	Control N	Relative risk [95% CI]
Unstable CAD – Unrevascularized post–MI					
TOPS, 1992	0	42	0	45	1.07 [0.02, 52.73]
DANAMI, 1997	28	503	53	505	0.53 [0.34, 0.82]
DAKIK, 1998	2	21	0	23	5.45 [0.28, 107.47]
Horie, 1998	3	44	7	39	0.38 [0.11, 1.37]
TOAT, 2002	3	32	1	34	3.19 [0.35, 29.09]
ALKK, 2003	10	149	12	151	0.84 [0.38, 1.89]
DECOPI, 2004	4	109	3	103	1.26 [0.29, 5.49]
OAT, 2006	57	1082	40	1084	1.43 [0.96, 2.12]
INSPIRE, 2006	5	104	7	101	0.69 [0.23, 2.11]
SWISSI 2, 2007	11	96	40	105	0.30 [0.16, 0.55]
VIAMI, 2012	2	106	2	110	1.04 [0.15, 7.23]
Unrevascularized post–MI studies (**p = 0.03**, Q = 26.36, df = 10, p for heterogeneity = 0.00; I^2 = 62.1%)					0.77 [0.61, 0.97]
Unstable CAD – Multivessel disease following STEMI					
Help–AMI, 2009	1	52	1	17	0.33 [0.02, 4.95]
Politi, 2010	6	130	7	84	0.55 [0.19, 1.59]
Dambrink, 2012	4	79	0	40	4.61 [0.25, 83.61]
PRAMI, 2013	7	234	20	231	0.35 [0.15, 0.80]
CvLPRIT, 2015	2	150	4	146	0.49 [0.09, 2.62]
DANAMI 3, 2015	15	313	16	314	0.94 [0.47, 1.87]
Zhang, 2015	9	215	14	213	0.64 [0.28, 1.44]
Hamza, 2016	1	50	2	50	0.50 [0.05, 5.34]
Compare ACUTE, 2017	7	295	28	590	0.50 [0.22, 1.13]
Complete, 2019	109	2016	160	2025	0.68 [0.54, 0.87]
Multivessel disease following STEMI studies (**p < 0.0001**, Q = 6.10, df = 9, p for heterogeneity = 0.73; I^2 = 0.0%)					0.66 [0.54, 0.80]
Unstable CAD – NSTEACS					
TIMI IIIB, 1994	38	740	42	733	0.90 [0.58, 1.37]
FRISC II, 2000	105	1219	143	1234	0.74 [0.59, 0.94]
TRUCS, 2000	3	76	3	72	0.95 [0.20, 4.54]
TACTICS 18, 2001	44	1114	66	1106	0.66 [0.46, 0.96]
RITA 3, 2002	45	895	56	915	0.82 [0.56, 1.20]
VINO, 2002	2	64	10	67	0.21 [0.05, 0.92]
ICTUS, 2005	90	604	59	596	1.51 [1.11, 2.05]
Savonitto, 2012	11	154	17	159	0.67 [0.32, 1.38]
After 80, 2016	39	229	69	228	0.56 [0.40, 0.80]
Sanchis, 2016	16	52	11	54	1.51 [0.78, 2.94]
NSTEMI studies (**p = 0.01**, Q = 28.31, df = 9.00, p for heterogeneity = 0.00; I^2 = 68.2%)					0.83 [0.73, 0.95]
**Unstable CAD studies (p = 0.000, Q = 64.78, df = 30.00, p for heterogeneity = 0.00; I^2 = 53.7%)**					0.77 [0.70, 0.85]
Stable CAD					
ACME 1, 1992	5	105	3	107	1.70 [0.42, 6.93]
MASS 1 PCI ONLY, 1995	2	72	2	72	1.00 [0.14, 6.91]
ACME 2, 1997	6	51	6	50	0.98 [0.34, 2.84]
RITA 2, 1997	21	504	10	514	2.14 [1.02, 4.50]
ACIP, 1997	7	192	18	366	0.74 [0.32, 1.74]
AVERT, 1999	4	177	5	164	0.74 [0.20, 2.71]
DEFER, 2001	3	90	0	91	7.08 [0.37, 135.07]
TIME, 2001	12	153	17	148	0.68 [0.34, 1.38]
MASS 2 PCI ONLY, 2004	16	205	10	203	1.58 [0.74, 3.41]
Hambrecht, 2004	1	50	0	51	3.06 [0.13, 73.35]
COURAGE, 2007	143	1149	128	1138	1.11 [0.88, 1.38]
JSAP, 2008	3	188	7	191	0.44 [0.11, 1.66]
BARI 2D, 2009	118	1176	138	1192	0.87 [0.69, 1.09]
FAME 2, 2012	15	447	14	441	1.06 [0.52, 2.16]
ISCHEMIA, 2019	206	2588	225	2591	0.92 [0.76, 1.10]
**Stable CAD studies (p = 0.62, Q = 14.34, df = 14.00, p for heterogeneity = 0.42; I^2 = 2.4%)**					0.97 [0.87, 1.09]

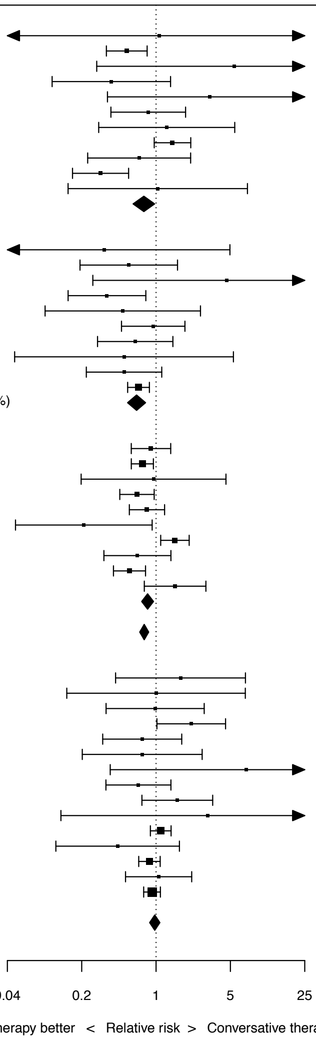

Online Figure 11. Sensitivity analysis excluding trials that permitted CABG for all-cause mortality

Study and Year	Active Events	Active N	Control Events	Control N	Relative risk [95% CI]
Unstable CAD – Unrevascularized post–MI					
TOPS, 1992	0	42	1	45	0.36 [0.01, 8.52]
DAKIK, 1998	1	21	1	23	1.10 [0.07, 16.43]
Horie, 1998	1	44	5	39	0.18 [0.02, 1.45]
TOAT, 2002	2	32	1	34	2.13 [0.20, 22.31]
ALKK, 2003	6	149	17	151	0.36 [0.15, 0.88]
DECOPI, 2004	8	109	9	103	0.84 [0.34, 2.09]
OAT, 2006	87	1082	84	1084	1.04 [0.78, 1.38]
SWISSI 2, 2007	6	96	22	105	0.30 [0.13, 0.70]
VIAMI, 2012	2	106	3	110	0.69 [0.12, 4.06]
Unrevascularized post–MI studies (**p = 0.06**, Q = 14.14, df = 8, p for heterogeneity = 0.08; I^2 = 44.2%)					0.61 [0.37, 1.02]
Unstable CAD – Multivessel disease following STEMI					
Help–AMI, 2009	1	52	0	17	1.02 [0.04, 23.91]
Politi, 2010	10	130	13	84	0.50 [0.23, 1.08]
Dambrink, 2012	2	79	0	40	2.56 [0.13, 52.14]
PRAMI, 2013	12	234	16	231	0.74 [0.36, 1.53]
CvLPRIT, 2015	4	150	10	146	0.39 [0.12, 1.21]
DANAMI 3, 2015	15	313	11	314	1.37 [0.64, 2.93]
Zhang, 2015	13	215	15	213	0.86 [0.42, 1.76]
Hamza, 2016	1	50	4	50	0.25 [0.03, 2.16]
Compare ACUTE, 2017	4	295	10	590	0.80 [0.25, 2.53]
Complete, 2019	96	2016	106	2025	0.91 [0.70, 1.19]
Multivessel disease following STEMI studies (**p = 0.11**, Q = 7.30, df = 9, p for heterogeneity = 0.61; I^2 = 0.0%)					0.84 [0.69, 1.04]
**Unstable CAD studies (p = 0.018, Q = 21.45, df = 18.00, p for heterogeneity = 0.26; I^2 = 31.0%)**					0.74 [0.58, 0.95]
Stable CAD					
ACME 1, 1992	0	105	1	107	0.34 [0.01, 8.24]
MASS 1 PCI ONLY, 1995	1	72	0	72	3.00 [0.12, 72.44]
ACME 2, 1997	9	51	10	50	0.88 [0.39, 1.99]
RITA 2, 1997	11	504	7	514	1.60 [0.63, 4.10]
ACIP, 1997	2	192	20	366	0.19 [0.05, 0.81]
AVERT, 1999	1	177	1	164	0.93 [0.06, 14.69]
DEFER, 2001	2	90	4	91	0.51 [0.09, 2.69]
MASS 2 PCI ONLY, 2004	9	205	3	203	2.97 [0.82, 10.82]
Hambrecht, 2004	0	50	0	51	1.02 [0.02, 50.41]
COURAGE, 2007	85	1149	95	1138	0.89 [0.67, 1.17]
JSAP, 2008	6	188	7	191	0.87 [0.30, 2.54]
FAME 2, 2012	1	447	3	441	0.33 [0.03, 3.15]
**Stable CAD studies (p = 0.38, Q = 11.32, df = 11.00, p for heterogeneity = 0.42; I^2 = 0.0%)**					0.90 [0.71, 1.14]

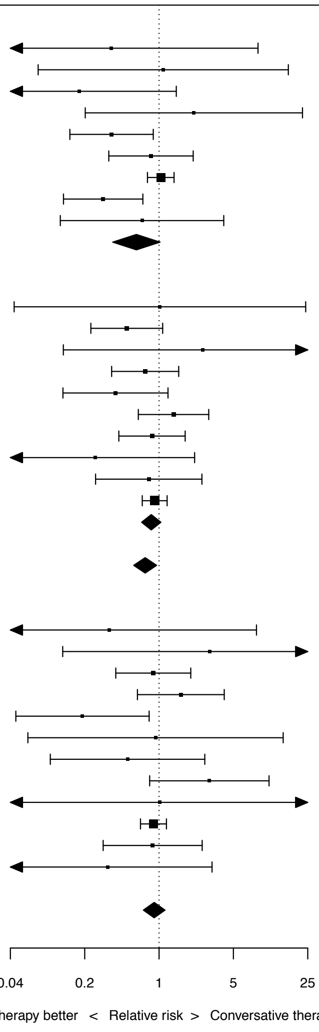

Online Figure 12. Sensitivity analysis excluding trials that permitted CABG for cardiac death

Study and Year	Active Events	Active N	Control Events	Control N	Relative risk [95% CI]
Unstable CAD – Unrevascularized post–MI					
TOPS, 1992	0	42	0	45	1.07 [0.02, 52.73]
DAKIK, 1998	1	21	1	23	1.10 [0.07, 16.43]
Horie, 1998	1	44	4	39	0.22 [0.03, 1.90]
ALKK, 2003	4	149	14	151	0.29 [0.10, 0.86]
DECOPI, 2004	6	109	7	103	0.81 [0.28, 2.33]
OAT, 2006	58	1082	52	1084	1.12 [0.78, 1.61]
SWISSI 2, 2007	3	96	22	105	0.15 [0.05, 0.48]
Unrevascularized post–MI studies (**p = 0.07**, Q = 15.87, df = 6, p for heterogeneity = 0.01; I^2 = 60.0%)					0.50 [0.24, 1.07]
Unstable CAD – Multivessel disease following STEMI					
Politi, 2010	6	130	10	84	0.39 [0.15, 1.03]
PRAMI, 2013	4	234	10	231	0.39 [0.13, 1.24]
CvLPRIT, 2015	2	150	7	146	0.28 [0.06, 1.32]
DANAMI 3, 2015	5	313	9	314	0.56 [0.19, 1.64]
Zhang, 2015	11	215	14	213	0.78 [0.36, 1.68]
Compare ACUTE, 2017	3	295	6	590	1.00 [0.25, 3.97]
Complete, 2019	59	2016	64	2025	0.93 [0.65, 1.31]
Multivessel disease following STEMI studies (**p = 0.04**, Q = 6.40, df = 6, p for heterogeneity = 0.38; I^2 = 21.8%)					0.68 [0.47, 0.98]
**Unstable CAD studies (p = 0.006, Q = 22.41, df = 13.00, p for heterogeneity = 0.05; I^2 = 50.3%)**					0.59 [0.41, 0.86]
Stable CAD					
ACME 1, 1992	0	105	1	107	0.34 [0.01, 8.24]
RITA 2, 1997	5	504	3	514	1.70 [0.41, 7.07]
AVERT, 1999	1	177	1	164	0.93 [0.06, 14.69]
DEFER, 2001	1	90	2	90	0.50 [0.05, 5.42]
MASS 2 PCI ONLY, 2004	9	205	3	203	2.97 [0.82, 10.82]
Hambrecht, 2004	0	50	0	51	1.02 [0.02, 50.41]
COURAGE, 2007	23	1149	25	1138	0.91 [0.52, 1.60]
JSAP, 2008	2	188	3	191	0.68 [0.11, 4.01]
FAME 2, 2012	1	447	1	441	0.99 [0.06, 15.72]
**Stable CAD studies (p = 0.83, Q = 4.27, df = 8.00, p for heterogeneity = 0.83; I^2 = 0.0%)**					1.05 [0.68, 1.63]

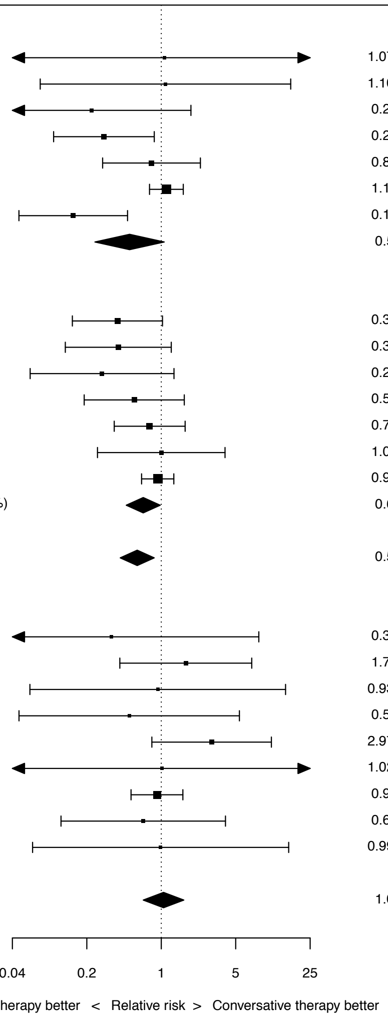

Online Figure 13. Sensitivity analysis excluding trials that permitted CABG for myocardial infarction

Study and Year	Active Events	Active N	Control Events	Control N	Relative risk [95% CI]
Unstable CAD – Unrevascularized post–MI					
TOPS, 1992	0	42	0	45	1.07 [0.02, 52.73]
DAKIK, 1998	2	21	0	23	5.45 [0.28, 107.47]
Horie, 1998	3	44	7	39	0.38 [0.11, 1.37]
TOAT, 2002	3	32	1	34	3.19 [0.35, 29.09]
ALKK, 2003	10	149	12	151	0.84 [0.38, 1.89]
DECOPI, 2004	4	109	3	103	1.26 [0.29, 5.49]
OAT, 2006	57	1082	40	1084	1.43 [0.96, 2.12]
SWISSI 2, 2007	11	96	40	105	0.30 [0.16, 0.55]
VIAMI, 2012	2	106	2	110	1.04 [0.15, 7.23]
Unrevascularized post–MI studies (**p = 0.62**, Q = 22.40, df = 8, p for heterogeneity = 0.00; I^2 = 59.0%)					0.86 [0.47, 1.56]
Unstable CAD – Multivessel disease following STEMI					
Help–AMI, 2009	1	52	1	17	0.33 [0.02, 4.95]
Politi, 2010	6	130	7	84	0.55 [0.19, 1.59]
Dambrink, 2012	4	79	0	40	4.61 [0.25, 83.61]
PRAMI, 2013	7	234	20	231	0.35 [0.15, 0.80]
CvLPRIT, 2015	2	150	4	146	0.49 [0.09, 2.62]
DANAMI 3, 2015	15	313	16	314	0.94 [0.47, 1.87]
Zhang, 2015	9	215	14	213	0.64 [0.28, 1.44]
Hamza, 2016	1	50	2	50	0.50 [0.05, 5.34]
Compare ACUTE, 2017	7	295	28	590	0.50 [0.22, 1.13]
Complete, 2019	109	2016	160	2025	0.68 [0.54, 0.87]
Multivessel disease following STEMI studies (**p = 0.00**, Q = 6.10, df = 9, p for heterogeneity = 0.73; I^2 = 0.0%)					0.66 [0.54, 0.80]
**Unstable CAD studies (p = 0.019, Q = 31.77, df = 18.00, p for heterogeneity = 0.02; I^2 = 47.6%)**					0.70 [0.51, 0.94]
Stable CAD					
ACME 1, 1992	5	105	3	107	1.70 [0.42, 6.93]
MASS 1 PCI ONLY, 1995	2	72	2	72	1.00 [0.14, 6.91]
ACME 2, 1997	6	51	6	50	0.98 [0.34, 2.84]
RITA 2, 1997	21	504	10	514	2.14 [1.02, 4.50]
ACIP, 1997	7	192	18	366	0.74 [0.32, 1.74]
AVERT, 1999	4	177	5	164	0.74 [0.20, 2.71]
DEFER, 2001	3	90	0	91	7.08 [0.37, 135.07]
MASS 2 PCI ONLY, 2004	16	205	10	203	1.58 [0.74, 3.41]
Hambrecht, 2004	1	50	0	51	3.06 [0.13, 73.35]
COURAGE, 2007	143	1149	128	1138	1.11 [0.88, 1.38]
JSAP, 2008	3	188	7	191	0.44 [0.11, 1.66]
FAME 2, 2012	15	447	14	441	1.06 [0.52, 2.16]
**Stable CAD studies (p = 0.17, Q = 9.22, df = 11.00, p for heterogeneity = 0.60; I^2 = 0.0%)**					1.14 [0.95, 1.37]

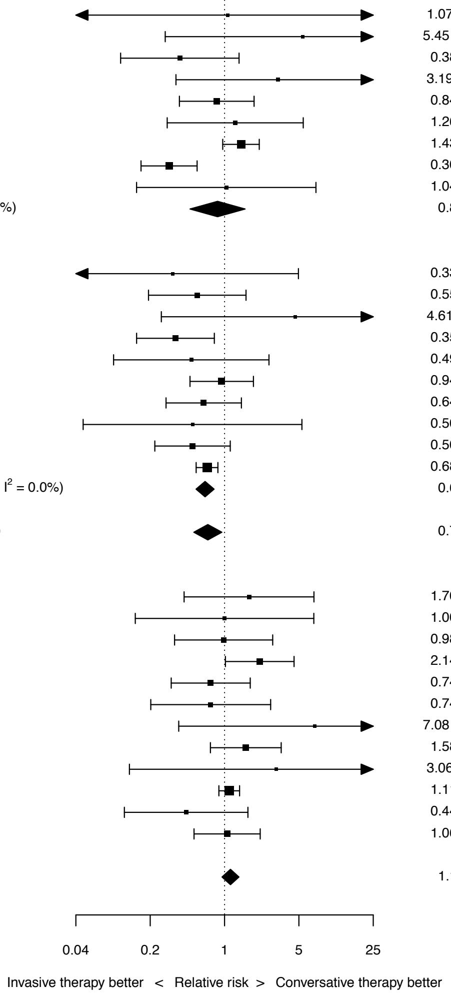

Online Figure 14. Sensitivity analysis for all-cause mortality excluding trials considered at high risk of bias

Study and Year	Active Events	Active N	Control Events	Control N	Relative risk [95% CI]
Unstable CAD – Unrevascularized post–MI					
TOPS, 1992	0	42	1	45	0.36 [0.01, 8.52]
DANAMI, 1997	18	503	22	505	0.82 [0.45, 1.51]
DAKIK, 1998	1	21	1	23	1.10 [0.07, 16.43]
Horie, 1998	1	44	5	39	0.18 [0.02, 1.45]
TOAT, 2002	2	32	1	34	2.13 [0.20, 22.31]
ALKK, 2003	6	149	17	151	0.36 [0.15, 0.88]
DECOPI, 2004	8	109	9	103	0.84 [0.34, 2.09]
OAT, 2006	87	1082	84	1084	1.04 [0.78, 1.38]
INSPIRE, 2006	2	104	1	101	1.94 [0.18, 21.09]
SWISSI 2, 2007	6	96	22	105	0.30 [0.13, 0.70]
Unrevascularized post–MI studies (**p = 0.13**, Q = 14.58, df = 9, p for heterogeneity = 0.10; I^2 = 38.3%)					0.84 [0.67, 1.05]
Unstable CAD – Multivessel disease following STEMI					
CvLPRIT, 2015	4	150	10	146	0.39 [0.12, 1.21]
DANAMI 3, 2015	15	313	11	314	1.37 [0.64, 2.93]
Compare ACUTE, 2017	4	295	10	590	0.80 [0.25, 2.53]
Complete, 2019	96	2016	106	2025	0.91 [0.70, 1.19]
Multivessel disease following STEMI studies (**p = 0.43**, Q = 3.29, df = 3, p for heterogeneity = 0.35; I^2 = 8.8%)					0.91 [0.71, 1.16]
Unstable CAD – NSTEACS					
TIMI IIIB, 1994	18	740	18	733	0.99 [0.52, 1.89]
FRISC II, 2000	27	1222	48	1234	0.57 [0.36, 0.90]
TACTICS 18, 2001	37	1114	39	1106	0.94 [0.61, 1.47]
RITA 3, 2002	60	895	72	915	0.85 [0.61, 1.19]
VINO, 2002	2	64	9	67	0.23 [0.05, 1.04]
ICTUS, 2005	15	604	15	596	0.99 [0.49, 2.00]
Savonitto, 2012	19	154	22	159	0.89 [0.50, 1.58]
After 80, 2016	57	229	62	228	0.92 [0.67, 1.25]
Sanchis, 2016	22	52	26	54	0.88 [0.58, 1.34]
NSTEMI studies (**p = 0.03**, Q = 6.64, df = 8.00, p for heterogeneity = 0.58; I^2 = 0.0%)					0.85 [0.73, 0.99]
**Unstable CAD studies (p = 0.007, Q = 24.78, df = 22.00, p for heterogeneity = 0.31; I^2 = 11.2%)**					0.86 [0.77, 0.96]
Stable CAD					
RITA 2, 1997	11	504	7	514	1.60 [0.63, 4.10]
DEFER, 2001	2	90	4	91	0.51 [0.09, 2.69]
TIME, 2001	13	153	6	148	2.10 [0.82, 5.37]
MASS 2 PCI ONLY, 2004	9	205	3	203	2.97 [0.82, 10.82]
COURAGE, 2007	85	1149	95	1138	0.89 [0.67, 1.17]
JSAP, 2008	6	188	7	191	0.87 [0.30, 2.54]
BARI 2D, 2009	155	1176	161	1192	0.98 [0.79, 1.20]
FAME 2, 2012	1	447	3	441	0.33 [0.03, 3.15]
**Stable CAD studies (p = 0.84, Q = 8.43, df = 7.00, p for heterogeneity = 0.30; I^2 = 17.0%)**					0.98 [0.84, 1.15]

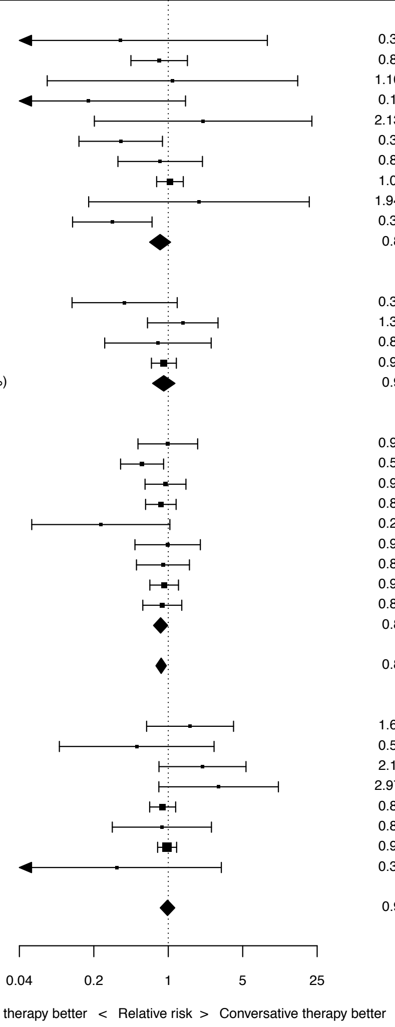

Online Figure 15. Sensitivity analysis for cardiac death excluding trials considered at high risk of bias

Study and Year	Active Events	Active N	Control Events	Control N	Relative risk [95% CI]
Unstable CAD – Unrevascularized post–MI					
TOPS, 1992	0	42	0	45	1.07 [0.02, 52.73]
DAKIK, 1998	1	21	1	23	1.10 [0.07, 16.43]
Horie, 1998	1	44	4	39	0.22 [0.03, 1.90]
ALKK, 2003	4	149	14	151	0.29 [0.10, 0.86]
DECOPI, 2004	6	109	7	103	0.81 [0.28, 2.33]
OAT, 2006	58	1082	52	1084	1.12 [0.78, 1.61]
INSPIRE, 2006	2	104	1	101	1.94 [0.18, 21.09]
SWISSI 2, 2007	3	96	22	105	0.15 [0.05, 0.48]
Unrevascularized post–MI studies (**p = 0.23**, Q = 16.36, df = 7, p for heterogeneity = 0.02; I^2 = 57.2%)					0.83 [0.61, 1.13]
Unstable CAD – Multivessel disease following STEMI					
CvLPRIT, 2015	2	150	7	146	0.28 [0.06, 1.32]
DANAMI 3, 2015	5	313	9	314	0.56 [0.19, 1.64]
Compare ACUTE, 2017	3	295	6	590	1.00 [0.25, 3.97]
Complete, 2019	59	2016	64	2025	0.93 [0.65, 1.31]
Multivessel disease following STEMI studies (**p = 0.30**, Q = 2.85, df = 3, p for heterogeneity = 0.41; I^2 = 0.0%)					0.85 [0.62, 1.16]
Unstable CAD – NSTEACS					
RITA 3, 2002	55	895	74	915	0.76 [0.54, 1.06]
Savonitto, 2012	16	154	17	159	0.97 [0.51, 1.85]
NSTEMI studies (**p = 0.14**, Q = 0.44, df = 1.00, p for heterogeneity = 0.51; I^2 = 0.0%)					0.80 [0.59, 1.08]
**Unstable CAD studies (p = 0.033, Q = 19.72, df = 13.00, p for heterogeneity = 0.10; I^2 = 34.1%)**					0.82 [0.69, 0.98]
Stable CAD					
RITA 2, 1997	5	504	3	514	1.70 [0.41, 7.07]
DEFER, 2001	1	90	2	90	0.50 [0.05, 5.42]
MASS 2 PCI ONLY, 2004	9	205	3	203	2.97 [0.82, 10.82]
COURAGE, 2007	23	1149	25	1138	0.91 [0.52, 1.60]
JSAP, 2008	2	188	3	191	0.68 [0.11, 4.01]
FAME 2, 2012	1	447	1	441	0.99 [0.06, 15.72]
**Stable CAD studies (p = 0.74, Q = 3.77, df = 5.00, p for heterogeneity = 0.58; I^2 = 0.0%)**					1.08 [0.69, 1.69]

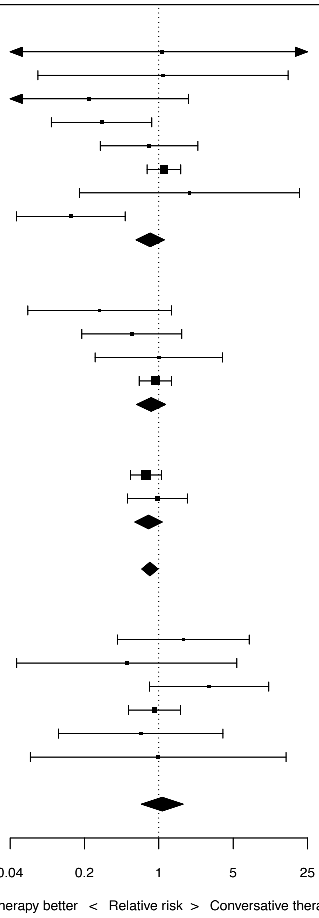

Online Figure 16. Sensitivity analysis for myocardial infarction excluding trials considered at high risk of bias

Study and Year	Active Events	Active N	Control Events	Control N	Relative risk [95% CI]
Unstable CAD – Unrevascularized post–MI					
TOPS, 1992	0	42	0	45	1.07 [0.02, 52.73]
DANAMI, 1997	28	503	53	505	0.53 [0.34, 0.82]
DAKIK, 1998	2	21	0	23	5.45 [0.28, 107.47]
Horie, 1998	3	44	7	39	0.38 [0.11, 1.37]
TOAT, 2002	3	32	1	34	3.19 [0.35, 29.09]
ALKK, 2003	10	149	12	151	0.84 [0.38, 1.89]
DECOPI, 2004	4	109	3	103	1.26 [0.29, 5.49]
OAT, 2006	57	1082	40	1084	1.43 [0.96, 2.12]
INSPIRE, 2006	5	104	7	101	0.69 [0.23, 2.11]
SWISSI 2, 2007	11	96	40	105	0.30 [0.16, 0.55]
Unrevascularized post–MI studies (**p = 0.03**, Q = 26.27, df = 9, p for heterogeneity = 0.00; I^2 = 65.7%)					0.76 [0.60, 0.97]
Unstable CAD – Multivessel disease following STEMI					
CvLPRIT, 2015	2	150	4	146	0.49 [0.09, 2.62]
DANAMI 3, 2015	15	313	16	314	0.94 [0.47, 1.87]
Compare ACUTE, 2017	7	295	28	590	0.50 [0.22, 1.13]
Complete, 2019	109	2016	160	2025	0.68 [0.54, 0.87]
Multivessel disease following STEMI studies (**p < 0.0001**, Q = 1.55, df = 3, p for heterogeneity = 0.67; I^2 = 0.0%)					0.69 [0.56, 0.85]
Unstable CAD – NSTEACS					
TIMI IIIB, 1994	38	740	42	733	0.90 [0.58, 1.37]
FRISC II, 2000	105	1219	143	1234	0.74 [0.59, 0.94]
TACTICS 18, 2001	44	1114	66	1106	0.66 [0.46, 0.96]
RITA 3, 2002	45	895	56	915	0.82 [0.56, 1.20]
VINO, 2002	2	64	10	67	0.21 [0.05, 0.92]
ICTUS, 2005	90	604	59	596	1.51 [1.11, 2.05]
Savonitto, 2012	11	154	17	159	0.67 [0.32, 1.38]
After 80, 2016	39	229	69	228	0.56 [0.40, 0.80]
Sanchis, 2016	16	52	11	54	1.51 [0.78, 2.94]
NSTEMI studies (**p = 0.01**, Q = 28.28, df = 8.00, p for heterogeneity = 0.00; I^2 = 71.7%)					0.83 [0.73, 0.95]
**Unstable CAD studies (p = 0.000, Q = 58.43, df = 22.00, p for heterogeneity = 0.00; I^2 = 62.3%)**					0.79 [0.71, 0.87]
Stable CAD					
RITA 2, 1997	21	504	10	514	2.14 [1.02, 4.50]
DEFER, 2001	3	90	0	91	7.08 [0.37, 135.07]
TIME, 2001	12	153	17	148	0.68 [0.34, 1.38]
MASS 2 PCI ONLY, 2004	16	205	10	203	1.58 [0.74, 3.41]
COURAGE, 2007	143	1149	128	1138	1.11 [0.88, 1.38]
JSAP, 2008	3	188	7	191	0.44 [0.11, 1.66]
BARI 2D, 2009	118	1176	138	1192	0.87 [0.69, 1.09]
FAME 2, 2012	15	447	14	441	1.06 [0.52, 2.16]
**Stable CAD studies (p = 0.86, Q = 11.96, df = 7.00, p for heterogeneity = 0.10; I^2 = 41.5%)**					1.01 [0.88, 1.17]

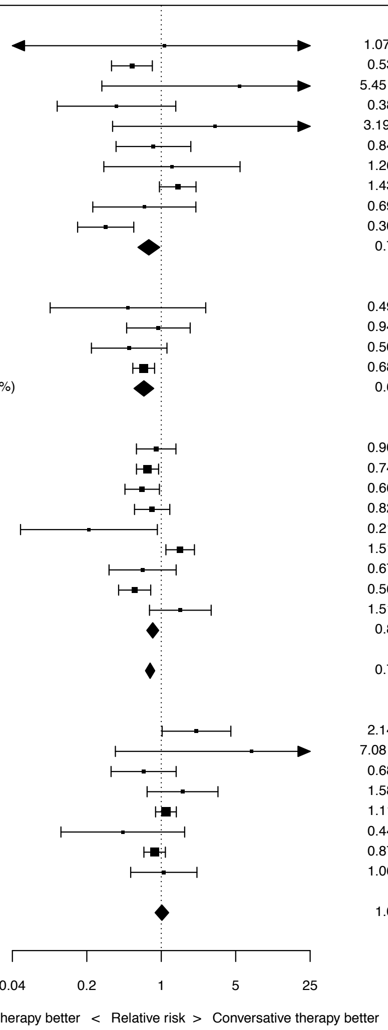

Online Figure 17. Sensitivity analysis for primary outcome of all-cause mortality excluding the ISCHEMIA trial

Study and Year	Active Events	Active N	Control Events	Control N	Relative risk [95% CI]
Unstable CAD – Unrevascularized post–MI					
TOPS, 1992	0	42	1	45	0.36 [0.01, 8.52]
DANAMI, 1997	18	503	22	505	0.82 [0.45, 1.51]
DAKIK, 1998	1	21	1	23	1.10 [0.07, 16.43]
Horie, 1998	1	44	5	39	0.18 [0.02, 1.45]
TOAT, 2002	2	32	1	34	2.13 [0.20, 22.31]
ALKK, 2003	6	149	17	151	0.36 [0.15, 0.88]
DECOPI, 2004	8	109	9	103	0.84 [0.34, 2.09]
OAT, 2006	87	1082	84	1084	1.04 [0.78, 1.38]
INSPIRE, 2006	2	104	1	101	1.94 [0.18, 21.09]
SWISSI 2, 2007	6	96	22	105	0.30 [0.13, 0.70]
VIAMI, 2012	2	106	3	110	0.69 [0.12, 4.06]
Unrevascularized post–MI studies (**p = 0.07**, Q = 14.63, df = 10, p for heterogeneity = 0.15; I^2 = 38.7%)					0.68 [0.45, 1.03]
Unstable CAD – Multivessel disease following STEMI					
Help–AMI, 2009	1	52	0	17	1.02 [0.04, 23.91]
Politi, 2010	10	130	13	84	0.50 [0.23, 1.08]
Dambrink, 2012	2	79	0	40	2.56 [0.13, 52.14]
PRAMI, 2013	12	234	16	231	0.74 [0.36, 1.53]
CvLPRIT, 2015	4	150	10	146	0.39 [0.12, 1.21]
DANAMI 3, 2015	15	313	11	314	1.37 [0.64, 2.93]
Zhang, 2015	13	215	15	213	0.86 [0.42, 1.76]
Hamza, 2016	1	50	4	50	0.25 [0.03, 2.16]
Compare ACUTE, 2017	4	295	10	590	0.80 [0.25, 2.53]
Complete, 2019	96	2016	106	2025	0.91 [0.70, 1.19]
Multivessel disease following STEMI studies (**p = 0.11**, Q = 7.30, df = 9, p for heterogeneity = 0.61; I^2 = 0.0%)					0.84 [0.69, 1.04]
Unstable CAD – NSTEACS					
TIMI IIIB, 1994	18	740	18	733	0.99 [0.52, 1.89]
FRISC II, 2000	27	1222	48	1234	0.57 [0.36, 0.90]
TRUCS, 2000	3	76	9	72	0.32 [0.09, 1.12]
TACTICS 18, 2001	37	1114	39	1106	0.94 [0.61, 1.47]
RITA 3, 2002	60	895	72	915	0.85 [0.61, 1.19]
VINO, 2002	2	64	9	67	0.23 [0.05, 1.04]
ICTUS, 2005	15	604	15	596	0.99 [0.49, 2.00]
Savonitto, 2012	19	154	22	159	0.89 [0.50, 1.58]
After 80, 2016	57	229	62	228	0.92 [0.67, 1.25]
Sanchis, 2016	22	52	26	54	0.88 [0.58, 1.34]
NSTEMI studies (**p = 0.02**, Q = 8.95, df = 9.00, p for heterogeneity = 0.44; I^2 = 0.0%)					0.84 [0.72, 0.97]
**Unstable CAD studies (p = 0.001, Q = 30.88, df = 30.00, p for heterogeneity = 0.42; I^2 = 0.0%)**					0.84 [0.75, 0.93]
Stable CAD					
ACME 1, 1992	0	105	1	107	0.34 [0.01, 8.24]
MASS 1 PCI ONLY, 1995	1	72	0	72	3.00 [0.12, 72.44]
ACME 2, 1997	9	51	10	50	0.88 [0.39, 1.99]
RITA 2, 1997	11	504	7	514	1.60 [0.63, 4.10]
ACIP, 1997	2	192	20	366	0.19 [0.05, 0.81]
AVERT, 1999	1	177	1	164	0.93 [0.06, 14.69]
DEFER, 2001	2	90	4	91	0.51 [0.09, 2.69]
TIME, 2001	13	153	6	148	2.10 [0.82, 5.37]
MASS 2 PCI ONLY, 2004	9	205	3	203	2.97 [0.82, 10.82]
Hambrecht, 2004	0	50	0	51	1.02 [0.02, 50.41]
COURAGE, 2007	85	1149	95	1138	0.89 [0.67, 1.17]
JSAP, 2008	6	188	7	191	0.87 [0.30, 2.54]
BARI 2D, 2009	155	1176	161	1192	0.98 [0.79, 1.20]
FAME 2, 2012	1	447	3	441	0.33 [0.03, 3.15]
**Stable CAD studies (p = 0.62, Q = 14.29, df = 13.00, p for heterogeneity = 0.35; I^2 = 0.0%)**					0.96 [0.83, 1.12]

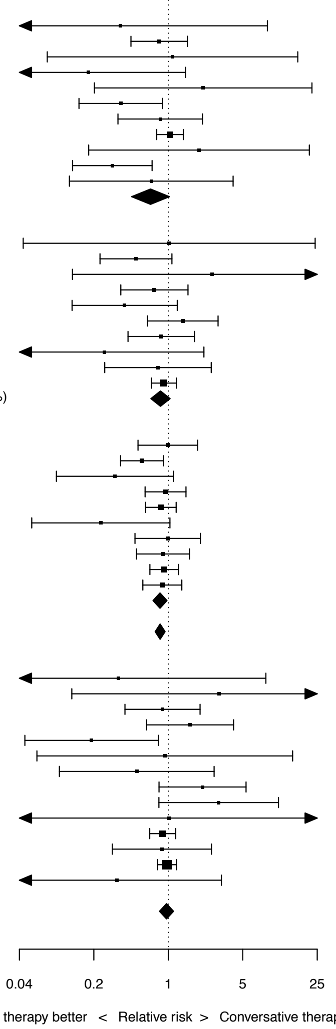

Online Figure 18. Sensitivity analysis for primary outcome of all-cause mortality excluding the ACIP trial

Study and Year	Active Events	Active N	Control Events	Control N	Relative risk [95% CI]
Unstable CAD – Unrevascularized post–MI					
TOPS, 1992	0	42	1	45	0.36 [0.01, 8.52]
DAKIK, 1998	1	21	1	23	1.10 [0.07, 16.43]
Horie, 1998	1	44	5	39	0.18 [0.02, 1.45]
ALKK, 2003	6	149	17	151	0.36 [0.15, 0.88]
DECOPI, 2004	8	109	9	103	0.84 [0.34, 2.09]
OAT, 2006	87	1082	84	1084	1.04 [0.78, 1.38]
SWISSI 2, 2007	6	96	22	105	0.30 [0.13, 0.70]
VIAMI, 2012	2	106	3	110	0.69 [0.12, 4.06]
Unrevascularized post–MI studies (**p = 0.04**, Q = 13.52, df = 7, p for heterogeneity = 0.06; I^2 = 48.3%)					0.58 [0.34, 0.98]
Unstable CAD – Multivessel disease following STEMI					
Help–AMI, 2009	1	52	0	17	1.02 [0.04, 23.91]
Politi, 2010	10	130	13	84	0.50 [0.23, 1.08]
Dambrink, 2012	2	79	0	40	2.56 [0.13, 52.14]
PRAMI, 2013	12	234	16	231	0.74 [0.36, 1.53]
CvLPRIT, 2015	4	150	10	146	0.39 [0.12, 1.21]
DANAMI 3, 2015	15	313	11	314	1.37 [0.64, 2.93]
Zhang, 2015	13	215	15	213	0.86 [0.42, 1.76]
Hamza, 2016	1	50	4	50	0.25 [0.03, 2.16]
Compare ACUTE, 2017	4	295	10	590	0.80 [0.25, 2.53]
Complete, 2019	96	2016	106	2025	0.91 [0.70, 1.19]
Multivessel disease following STEMI studies (**p = 0.11**, Q = 7.30, df = 9, p for heterogeneity = 0.61; I^2 = 0.0%)					0.84 [0.69, 1.04]
Unstable CAD – NSTEMI					
TIMI IIIB, 1994	18	740	18	733	0.99 [0.52, 1.89]
FRISC II, 2000	27	1222	48	1234	0.57 [0.36, 0.90]
TRUCS, 2000	3	76	9	72	0.32 [0.09, 1.12]
TACTICS 18, 2001	37	1114	39	1106	0.94 [0.61, 1.47]
RITA 3, 2002	60	895	72	915	0.85 [0.61, 1.19]
VINO, 2002	2	64	9	67	0.23 [0.05, 1.04]
ICTUS, 2005	15	604	15	596	0.99 [0.49, 2.00]
Savonitto, 2012	19	154	22	159	0.89 [0.50, 1.58]
After 80, 2016	57	229	62	228	0.92 [0.67, 1.25]
Sanchis, 2016	22	52	26	54	0.88 [0.58, 1.34]
NSTEMI studies (**p = 0.02**, Q = 8.95, df = 9.00, p for heterogeneity = 0.44; I^2 = 0.0%)					0.84 [0.72, 0.97]
**Unstable CAD studies (p = 0.001, Q = 29.79, df = 27.00, p for heterogeneity = 0.32; I^2 = 0.0%)**					0.84 [0.75, 0.93]
Stable CAD					
ACME 1, 1992	0	105	1	107	0.34 [0.01, 8.24]
MASS 1, 1995	1	72	0	72	3.00 [0.12, 72.44]
ACME 2, 1997	25	166	25	162	0.98 [0.59, 1.63]
RITA 2, 1997	11	504	7	514	1.60 [0.63, 4.10]
AVERT, 1999	1	177	1	164	0.93 [0.06, 14.69]
DEFER, 2001	2	90	4	91	0.51 [0.09, 2.69]
TIME, 2001	13	153	6	148	2.10 [0.82, 5.37]
MASS 2, 2004	9	205	3	203	2.97 [0.82, 10.82]
Hambrecht, 2004	0	50	0	51	1.02 [0.02, 50.41]
COURAGE, 2007	85	1149	95	1138	0.89 [0.67, 1.17]
JSAP, 2008	6	188	7	191	0.87 [0.30, 2.54]
BARI 2D, 2009	102	798	96	807	1.07 [0.83, 1.39]
FAME 2, 2012	1	447	3	441	0.33 [0.03, 3.15]
**Stable CAD studies (p = 0.78, Q = 9.54, df = 12.00, p for heterogeneity = 0.66; I^2 = 0.0%)**					1.02 [0.87, 1.21]

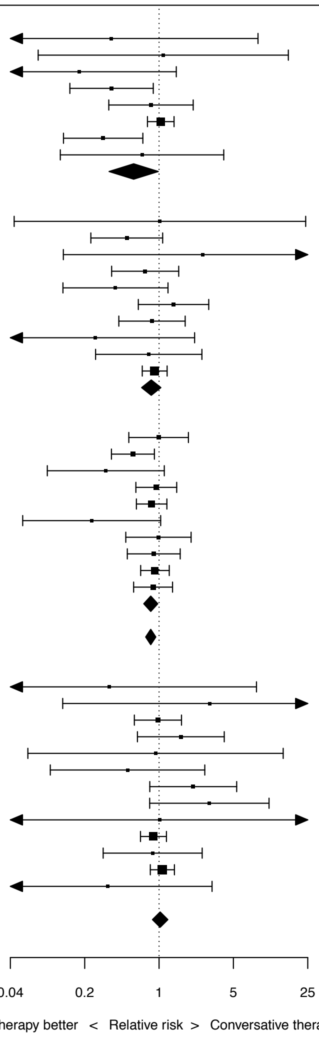

Online Figure 19. Sensitivity analysis for primary outcome of all-cause mortality excluding the ACME 1 trial

Study and Year	Active Events	N	Control Events	N	Relative risk [95% CI]
Unstable CAD – Unrevascularized post–MI					
TOPS, 1992	0	42	1	45	0.36 [0.01, 8.52]
DAKIK, 1998	1	21	1	23	1.10 [0.07, 16.43]
Horie, 1998	1	44	5	39	0.18 [0.02, 1.45]
ALKK, 2003	6	149	17	151	0.36 [0.15, 0.88]
DECOPI, 2004	8	109	9	103	0.84 [0.34, 2.09]
OAT, 2006	87	1082	84	1084	1.04 [0.78, 1.38]
SWISSI 2, 2007	6	96	22	105	0.30 [0.13, 0.70]
VIAMI, 2012	2	106	3	110	0.69 [0.12, 4.06]
Unrevascularized post–MI studies (**p = 0.04**, Q = 13.52, df = 7, p for heterogeneity = 0.06; I^2 = 48.3%)					0.58 [0.34, 0.98]
Unstable CAD – Multivessel disease following STEMI					
Help–AMI, 2009	1	52	0	17	1.02 [0.04, 23.91]
Politi, 2010	10	130	13	84	0.50 [0.23, 1.08]
Dambrink, 2012	2	79	0	40	2.56 [0.13, 52.14]
PRAMI, 2013	12	234	16	231	0.74 [0.36, 1.53]
CvLPRIT, 2015	4	150	10	146	0.39 [0.12, 1.21]
DANAMI 3, 2015	15	313	11	314	1.37 [0.64, 2.93]
Zhang, 2015	13	215	15	213	0.86 [0.42, 1.76]
Hamza, 2016	1	50	4	50	0.25 [0.03, 2.16]
Compare ACUTE, 2017	4	295	10	590	0.80 [0.25, 2.53]
Complete, 2019	96	2016	106	2025	0.91 [0.70, 1.19]
Multivessel disease following STEMI studies (**p = 0.11**, Q = 7.30, df = 9, p for heterogeneity = 0.61; I^2 = 0.0%)					0.84 [0.69, 1.04]
Unstable CAD – NSTEMI					
TIMI IIIB, 1994	18	740	18	733	0.99 [0.52, 1.89]
FRISC II, 2000	27	1222	48	1234	0.57 [0.36, 0.90]
TRUCS, 2000	3	76	9	72	0.32 [0.09, 1.12]
TACTICS 18, 2001	37	1114	39	1106	0.94 [0.61, 1.47]
RITA 3, 2002	60	895	72	915	0.85 [0.61, 1.19]
VINO, 2002	2	64	9	67	0.23 [0.05, 1.04]
ICTUS, 2005	15	604	15	596	0.99 [0.49, 2.00]
Savonitto, 2012	19	154	22	159	0.89 [0.50, 1.58]
After 80, 2016	57	229	62	228	0.92 [0.67, 1.25]
Sanchis, 2016	22	52	26	54	0.88 [0.58, 1.34]
NSTEMI studies (**p = 0.02**, Q = 8.95, df = 9.00, p for heterogeneity = 0.44; I^2 = 0.0%)					0.84 [0.72, 0.97]
**Unstable CAD studies (p = 0.001, Q = 29.79, df = 27.00, p for heterogeneity = 0.32; I^2 = 0.0%)**					0.84 [0.75, 0.93]
Stable CAD					
MASS 1, 1995	1	72	0	72	3.00 [0.12, 72.44]
ACME 2, 1997	25	166	25	162	0.98 [0.59, 1.63]
RITA 2, 1997	11	504	7	514	1.60 [0.63, 4.10]
ACIP, 1997	2	192	20	366	0.19 [0.05, 0.81]
AVERT, 1999	1	177	1	164	0.93 [0.06, 14.69]
DEFER, 2001	2	90	4	91	0.51 [0.09, 2.69]
TIME, 2001	13	153	6	148	2.10 [0.82, 5.37]
MASS 2, 2004	9	205	3	203	2.97 [0.82, 10.82]
Hambrecht, 2004	0	50	0	51	1.02 [0.02, 50.41]
COURAGE, 2007	85	1149	95	1138	0.89 [0.67, 1.17]
JSAP, 2008	6	188	7	191	0.87 [0.30, 2.54]
BARI 2D, 2009	102	798	96	807	1.07 [0.83, 1.39]
FAME 2, 2012	1	447	3	441	0.33 [0.03, 3.15]
**Stable CAD studies (p = 0.95, Q = 14.24, df = 12.00, p for heterogeneity = 0.29; I^2 = 0.0%)**					1.00 [0.85, 1.19]

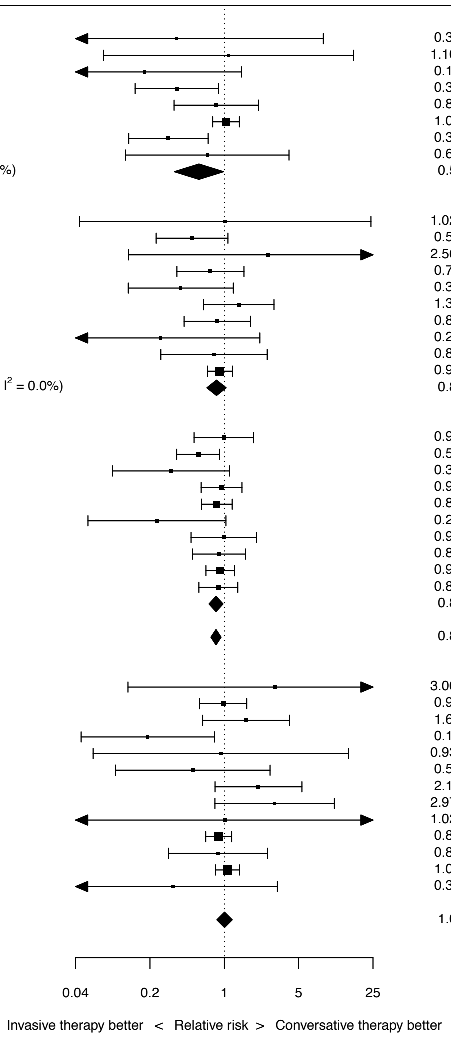

Online Figure 20. Sensitivity analysis for primary outcome of all-cause mortality excluding the ACME 2 trial

Study and Year	Active Events	N	Control Events	N	Relative risk [95% CI]
Unstable CAD – Unrevascularized post–MI					
TOPS, 1992	0	42	1	45	0.36 [0.01, 8.52]
DAKIK, 1998	1	21	1	23	1.10 [0.07, 16.43]
Horie, 1998	1	44	5	39	0.18 [0.02, 1.45]
ALKK, 2003	6	149	17	151	0.36 [0.15, 0.88]
DECOPI, 2004	8	109	9	103	0.84 [0.34, 2.09]
OAT, 2006	87	1082	84	1084	1.04 [0.78, 1.38]
SWISSI 2, 2007	6	96	22	105	0.30 [0.13, 0.70]
VIAMI, 2012	2	106	3	110	0.69 [0.12, 4.06]
Unrevascularized post–MI studies (**p = 0.04**, Q = 13.52, df = 7, p for heterogeneity = 0.06; I^2 = 48.3%)					0.58 [0.34, 0.98]
Unstable CAD – Multivessel disease following STEMI					
Help–AMI, 2009	1	52	0	17	1.02 [0.04, 23.91]
Politi, 2010	10	130	13	84	0.50 [0.23, 1.08]
Dambrink, 2012	2	79	0	40	2.56 [0.13, 52.14]
PRAMI, 2013	12	234	16	231	0.74 [0.36, 1.53]
CvLPRIT, 2015	4	150	10	146	0.39 [0.12, 1.21]
DANAMI 3, 2015	15	313	11	314	1.37 [0.64, 2.93]
Zhang, 2015	13	215	15	213	0.86 [0.42, 1.76]
Hamza, 2016	1	50	4	50	0.25 [0.03, 2.16]
Compare ACUTE, 2017	4	295	10	590	0.80 [0.25, 2.53]
Complete, 2019	96	2016	106	2025	0.91 [0.70, 1.19]
Multivessel disease following STEMI studies (**p = 0.11**, Q = 7.30, df = 9, p for heterogeneity = 0.61; I^2 = 0.0%)					0.84 [0.69, 1.04]
Unstable CAD – NSTEMI					
TIMI IIIB, 1994	18	740	18	733	0.99 [0.52, 1.89]
FRISC II, 2000	27	1222	48	1234	0.57 [0.36, 0.90]
TRUCS, 2000	3	76	9	72	0.32 [0.09, 1.12]
TACTICS 18, 2001	37	1114	39	1106	0.94 [0.61, 1.47]
RITA 3, 2002	60	895	72	915	0.85 [0.61, 1.19]
VINO, 2002	2	64	9	67	0.23 [0.05, 1.04]
ICTUS, 2005	15	604	15	596	0.99 [0.49, 2.00]
Savonitto, 2012	19	154	22	159	0.89 [0.50, 1.58]
After 80, 2016	57	229	62	228	0.92 [0.67, 1.25]
Sanchis, 2016	22	52	26	54	0.88 [0.58, 1.34]
NSTEMI studies (**p = 0.02**, Q = 8.95, df = 9.00, p for heterogeneity = 0.44; I^2 = 0.0%)					0.84 [0.72, 0.97]
**Unstable CAD studies (p = 0.001, Q = 29.79, df = 27.00, p for heterogeneity = 0.32; I^2 = 0.0%)**					0.84 [0.75, 0.93]
Stable CAD					
ACME 1, 1992	0	105	1	107	0.34 [0.01, 8.24]
MASS 1, 1995	1	72	0	72	3.00 [0.12, 72.44]
RITA 2, 1997	11	504	7	514	1.60 [0.63, 4.10]
ACIP, 1997	2	192	20	366	0.19 [0.05, 0.81]
AVERT, 1999	1	177	1	164	0.93 [0.06, 14.69]
DEFER, 2001	2	90	4	91	0.51 [0.09, 2.69]
TIME, 2001	13	153	6	148	2.10 [0.82, 5.37]
MASS 2, 2004	9	205	3	203	2.97 [0.82, 10.82]
Hambrecht, 2004	0	50	0	51	1.02 [0.02, 50.41]
COURAGE, 2007	85	1149	95	1138	0.89 [0.67, 1.17]
JSAP, 2008	6	188	7	191	0.87 [0.30, 2.54]
BARI 2D, 2009	102	798	96	807	1.07 [0.83, 1.39]
FAME 2, 2012	1	447	3	441	0.33 [0.03, 3.15]
**Stable CAD studies (p = 0.95, Q = 14.67, df = 12.00, p for heterogeneity = 0.26; I^2 = 1.2%)**					1.01 [0.84, 1.21]

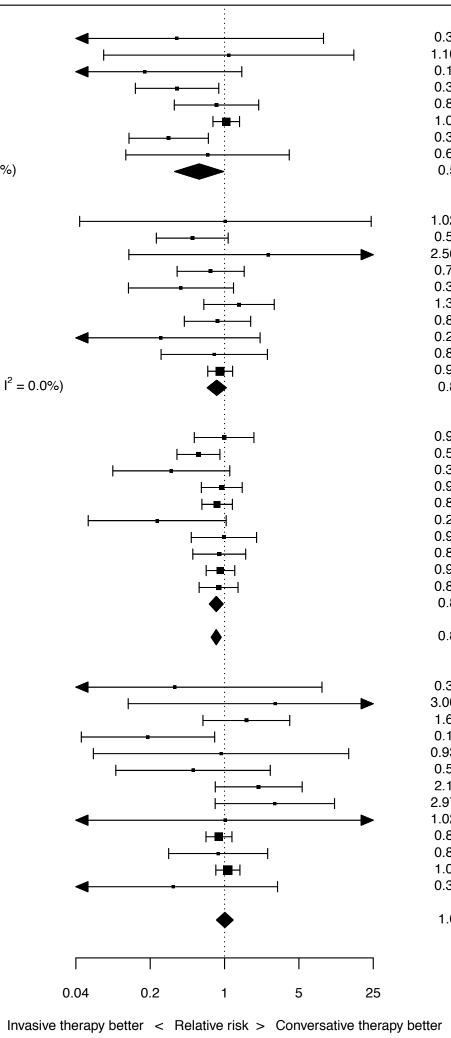

Online Figure 21. Sensitivity analysis for primary outcome of all-cause mortality excluding the After 80 trial

Study and Year	Active Events	N	Control Events	N	Relative risk [95% CI]
Unstable CAD – Unrevascularized post–MI					
TOPS, 1992	0	42	1	45	0.36 [0.01, 8.52]
DAKIK, 1998	1	21	1	23	1.10 [0.07, 16.43]
Horie, 1998	1	44	5	39	0.18 [0.02, 1.45]
ALKK, 2003	6	149	17	151	0.36 [0.15, 0.88]
DECOPI, 2004	8	109	9	103	0.84 [0.34, 2.09]
OAT, 2006	87	1082	84	1084	1.04 [0.78, 1.38]
SWISSI 2, 2007	6	96	22	105	0.30 [0.13, 0.70]
VIAMI, 2012	2	106	3	110	0.69 [0.12, 4.06]
Unrevascularized post–MI studies (**p = 0.04**, Q = 13.52, df = 7, p for heterogeneity = 0.06; I^2 = 48.3%)					0.58 [0.34, 0.98]
Unstable CAD – Multivessel disease following STEMI					
Help–AMI, 2009	1	52	0	17	1.02 [0.04, 23.91]
Politi, 2010	10	130	13	84	0.50 [0.23, 1.08]
Dambrink, 2012	2	79	0	40	2.56 [0.13, 52.14]
PRAMI, 2013	12	234	16	231	0.74 [0.36, 1.53]
CvLPRIT, 2015	4	150	10	146	0.39 [0.12, 1.21]
DANAMI 3, 2015	15	313	11	314	1.37 [0.64, 2.93]
Zhang, 2015	13	215	15	213	0.86 [0.42, 1.76]
Hamza, 2016	1	50	4	50	0.25 [0.03, 2.16]
Compare ACUTE, 2017	4	295	10	590	0.80 [0.25, 2.53]
Complete, 2019	96	2016	106	2025	0.91 [0.70, 1.19]
Multivessel disease following STEMI studies (**p = 0.11**, Q = 7.30, df = 9, p for heterogeneity = 0.61; I^2 = 0.0%)					0.84 [0.69, 1.04]
Unstable CAD – NSTEMI					
TIMI IIIB, 1994	18	740	18	733	0.99 [0.52, 1.89]
FRISC II, 2000	27	1222	48	1234	0.57 [0.36, 0.90]
TRUCS, 2000	3	76	9	72	0.32 [0.09, 1.12]
TACTICS 18, 2001	37	1114	39	1106	0.94 [0.61, 1.47]
RITA 3, 2002	60	895	72	915	0.85 [0.61, 1.19]
VINO, 2002	2	64	9	67	0.23 [0.05, 1.04]
ICTUS, 2005	15	604	15	596	0.99 [0.49, 2.00]
Savonitto, 2012	19	154	22	159	0.89 [0.50, 1.58]
Sanchis, 2016	22	52	26	54	0.88 [0.58, 1.34]
NSTEMI studies (**p = 0.02**, Q = 8.51, df = 8.00, p for heterogeneity = 0.39; I^2 = 0.0%)					0.81 [0.68, 0.97]
**Unstable CAD studies (p = 0.001, Q = 29.40, df = 26.00, p for heterogeneity = 0.29; I^2 = 7.8%)**					0.81 [0.71, 0.92]
Stable CAD					
ACME 1, 1992	0	105	1	107	0.34 [0.01, 8.24]
MASS 1, 1995	1	72	0	72	3.00 [0.12, 72.44]
ACME 2, 1997	25	166	25	162	0.98 [0.59, 1.63]
RITA 2, 1997	11	504	7	514	1.60 [0.63, 4.10]
ACIP, 1997	2	192	20	366	0.19 [0.05, 0.81]
AVERT, 1999	1	177	1	164	0.93 [0.06, 14.69]
DEFER, 2001	2	90	4	91	0.51 [0.09, 2.69]
TIME, 2001	13	153	6	148	2.10 [0.82, 5.37]
MASS 2, 2004	9	205	3	203	2.97 [0.82, 10.82]
Hambrecht, 2004	0	50	0	51	1.02 [0.02, 50.41]
COURAGE, 2007	85	1149	95	1138	0.89 [0.67, 1.17]
JSAP, 2008	6	188	7	191	0.87 [0.30, 2.54]
BARI 2D, 2009	102	798	96	807	1.07 [0.83, 1.39]
FAME 2, 2012	1	447	3	441	0.33 [0.03, 3.15]
**Stable CAD studies (p = 0.98, Q = 14.69, df = 13.00, p for heterogeneity = 0.33; I^2 = 0.0%)**					1.00 [0.85, 1.18]

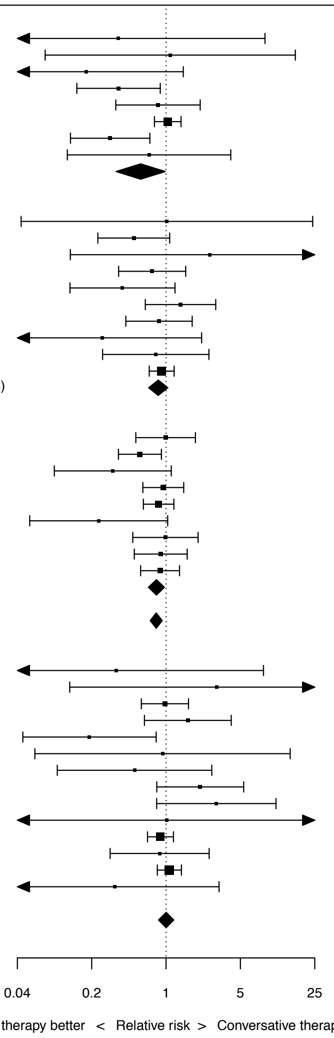

Online Figure 22. Sensitivity analysis for primary outcome of all-cause mortality excluding the ALKK trial

Study and Year	Active Events	N	Control Events	N	Relative risk [95% CI]
Unstable CAD – Unrevascularized post–MI					
TOPS, 1992	0	42	1	45	0.36 [0.01, 8.52]
DAKIK, 1998	1	21	1	23	1.10 [0.07, 16.43]
Horie, 1998	1	44	5	39	0.18 [0.02, 1.45]
DECOPI, 2004	8	109	9	103	0.84 [0.34, 2.09]
OAT, 2006	87	1082	84	1084	1.04 [0.78, 1.38]
SWISSI 2, 2007	6	96	22	105	0.30 [0.13, 0.70]
VIAMI, 2012	2	106	3	110	0.69 [0.12, 4.06]
Unrevascularized post–MI studies (**p = 0.13**, Q = 10.00, df = 6, p for heterogeneity = 0.12; I^2 = 45.1%)					0.64 [0.35, 1.14]
Unstable CAD – Multivessel disease following STEMI					
Help–AMI, 2009	1	52	0	17	1.02 [0.04, 23.91]
Politi, 2010	10	130	13	84	0.50 [0.23, 1.08]
Dambrink, 2012	2	79	0	40	2.56 [0.13, 52.14]
PRAMI, 2013	12	234	16	231	0.74 [0.36, 1.53]
CvLPRIT, 2015	4	150	10	146	0.39 [0.12, 1.21]
DANAMI 3, 2015	15	313	11	314	1.37 [0.64, 2.93]
Zhang, 2015	13	215	15	213	0.86 [0.42, 1.76]
Hamza, 2016	1	50	4	50	0.25 [0.03, 2.16]
Compare ACUTE, 2017	4	295	10	590	0.80 [0.25, 2.53]
Complete, 2019	96	2016	106	2025	0.91 [0.70, 1.19]
Multivessel disease following STEMI studies (**p = 0.11**, Q = 7.30, df = 9, p for heterogeneity = 0.61; I^2 = 0.0%)					0.84 [0.69, 1.04]
Unstable CAD – NSTEMI					
TIMI IIIB, 1994	18	740	18	733	0.99 [0.52, 1.89]
FRISC II, 2000	27	1222	48	1234	0.57 [0.36, 0.90]
TRUCS, 2000	3	76	9	72	0.32 [0.09, 1.12]
TACTICS 18, 2001	37	1114	39	1106	0.94 [0.61, 1.47]
RITA 3, 2002	60	895	72	915	0.85 [0.61, 1.19]
VINO, 2002	2	64	9	67	0.23 [0.05, 1.04]
ICTUS, 2005	15	604	15	596	0.99 [0.49, 2.00]
Savonitto, 2012	19	154	22	159	0.89 [0.50, 1.58]
After 80, 2016	57	229	62	228	0.92 [0.67, 1.25]
Sanchis, 2016	22	52	26	54	0.88 [0.58, 1.34]
NSTEMI studies (**p = 0.02**, Q = 8.95, df = 9.00, p for heterogeneity = 0.44; I^2 = 0.0%)					0.84 [0.72, 0.97]
**Unstable CAD studies (p = 0.003, Q = 26.35, df = 26.00, p for heterogeneity = 0.44; I^2 = 0.0%)**					0.85 [0.76, 0.94]
Stable CAD					
ACME 1, 1992	0	105	1	107	0.34 [0.01, 8.24]
MASS 1, 1995	1	72	0	72	3.00 [0.12, 72.44]
ACME 2, 1997	25	166	25	162	0.98 [0.59, 1.63]
RITA 2, 1997	11	504	7	514	1.60 [0.63, 4.10]
ACIP, 1997	2	192	20	366	0.19 [0.05, 0.81]
AVERT, 1999	1	177	1	164	0.93 [0.06, 14.69]
DEFER, 2001	2	90	4	91	0.51 [0.09, 2.69]
TIME, 2001	13	153	6	148	2.10 [0.82, 5.37]
MASS 2, 2004	9	205	3	203	2.97 [0.82, 10.82]
Hambrecht, 2004	0	50	0	51	1.02 [0.02, 50.41]
COURAGE, 2007	85	1149	95	1138	0.89 [0.67, 1.17]
JSAP, 2008	6	188	7	191	0.87 [0.30, 2.54]
BARI 2D, 2009	102	798	96	807	1.07 [0.83, 1.39]
FAME 2, 2012	1	447	3	441	0.33 [0.03, 3.15]
**Stable CAD studies (p = 0.98, Q = 14.69, df = 13.00, p for heterogeneity = 0.33; I^2 = 0.0%)**					1.00 [0.85, 1.18]

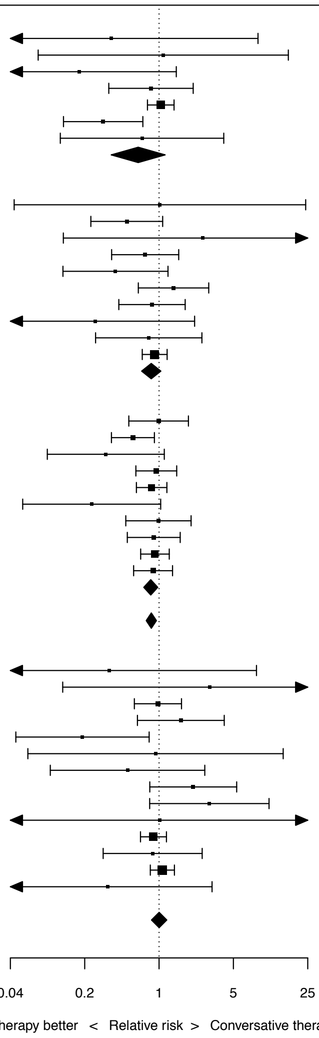

Online Figure 23. Sensitivity analysis for primary outcome of all-cause mortality excluding the AVERT trial

Study and Year	Active Events	N	Control Events	N	Relative risk [95% CI]
Unstable CAD – Unrevascularized post–MI					
TOPS, 1992	0	42	1	45	0.36 [0.01, 8.52]
DAKIK, 1998	1	21	1	23	1.10 [0.07, 16.43]
Horie, 1998	1	44	5	39	0.18 [0.02, 1.45]
ALKK, 2003	6	149	17	151	0.36 [0.15, 0.88]
DECOPI, 2004	8	109	9	103	0.84 [0.34, 2.09]
OAT, 2006	87	1082	84	1084	1.04 [0.78, 1.38]
SWISSI 2, 2007	6	96	22	105	0.30 [0.13, 0.70]
VIAMI, 2012	2	106	3	110	0.69 [0.12, 4.06]
Unrevascularized post–MI studies (**p = 0.04**, Q = 13.52, df = 7, p for heterogeneity = 0.06; I^2 = 48.3%)					0.58 [0.34, 0.98]
Unstable CAD – Multivessel disease following STEMI					
Help–AMI, 2009	1	52	0	17	1.02 [0.04, 23.91]
Politi, 2010	10	130	13	84	0.50 [0.23, 1.08]
Dambrink, 2012	2	79	0	40	2.56 [0.13, 52.14]
PRAMI, 2013	12	234	16	231	0.74 [0.36, 1.53]
CvLPRIT, 2015	4	150	10	146	0.39 [0.12, 1.21]
DANAMI 3, 2015	15	313	11	314	1.37 [0.64, 2.93]
Zhang, 2015	13	215	15	213	0.86 [0.42, 1.76]
Hamza, 2016	1	50	4	50	0.25 [0.03, 2.16]
Compare ACUTE, 2017	4	295	10	590	0.80 [0.25, 2.53]
Complete, 2019	96	2016	106	2025	0.91 [0.70, 1.19]
Multivessel disease following STEMI studies (**p = 0.11**, Q = 7.30, df = 9, p for heterogeneity = 0.61; I^2 = 0.0%)					0.84 [0.69, 1.04]
Unstable CAD – NSTEMI					
TIMI IIIB, 1994	18	740	18	733	0.99 [0.52, 1.89]
FRISC II, 2000	27	1222	48	1234	0.57 [0.36, 0.90]
TRUCS, 2000	3	76	9	72	0.32 [0.09, 1.12]
TACTICS 18, 2001	37	1114	39	1106	0.94 [0.61, 1.47]
RITA 3, 2002	60	895	72	915	0.85 [0.61, 1.19]
VINO, 2002	2	64	9	67	0.23 [0.05, 1.04]
ICTUS, 2005	15	604	15	596	0.99 [0.49, 2.00]
Savonitto, 2012	19	154	22	159	0.89 [0.50, 1.58]
After 80, 2016	57	229	62	228	0.92 [0.67, 1.25]
Sanchis, 2016	22	52	26	54	0.88 [0.58, 1.34]
NSTEMI studies (**p = 0.02**, Q = 8.95, df = 9.00, p for heterogeneity = 0.44; I^2 = 0.0%)					0.84 [0.72, 0.97]
**Unstable CAD studies (p = 0.001, Q = 29.79, df = 27.00, p for heterogeneity = 0.32; I^2 = 0.0%)**					0.84 [0.75, 0.93]
Stable CAD					
ACME 1, 1992	0	105	1	107	0.34 [0.01, 8.24]
MASS 1, 1995	1	72	0	72	3.00 [0.12, 72.44]
ACME 2, 1997	25	166	25	162	0.98 [0.59, 1.63]
RITA 2, 1997	11	504	7	514	1.60 [0.63, 4.10]
ACIP, 1997	2	192	20	366	0.19 [0.05, 0.81]
DEFER, 2001	2	90	4	91	0.51 [0.09, 2.69]
TIME, 2001	13	153	6	148	2.10 [0.82, 5.37]
MASS 2, 2004	9	205	3	203	2.97 [0.82, 10.82]
Hambrecht, 2004	0	50	0	51	1.02 [0.02, 50.41]
COURAGE, 2007	85	1149	95	1138	0.89 [0.67, 1.17]
JSAP, 2008	6	188	7	191	0.87 [0.30, 2.54]
BARI 2D, 2009	102	798	96	807	1.07 [0.83, 1.39]
FAME 2, 2012	1	447	3	441	0.33 [0.03, 3.15]
**Stable CAD studies (p = 0.98, Q = 14.68, df = 12.00, p for heterogeneity = 0.26; I^2 = 0.0%)**					1.00 [0.85, 1.18]

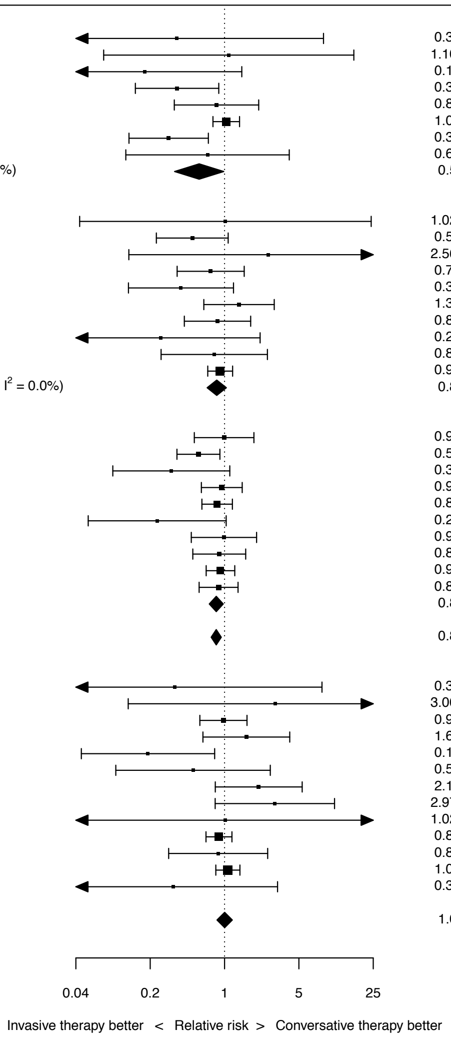

Online Figure 24. Sensitivity analysis for primary outcome of all-cause mortality excluding the BARI 2D trial

Study and Year	Active Events	N	Control Events	N	Relative risk [95% CI]
Unstable CAD – Unrevascularized post–MI					
TOPS, 1992	0	42	1	45	0.36 [0.01, 8.52]
DAKIK, 1998	1	21	1	23	1.10 [0.07, 16.43]
Horie, 1998	1	44	5	39	0.18 [0.02, 1.45]
ALKK, 2003	6	149	17	151	0.36 [0.15, 0.88]
DECOPI, 2004	8	109	9	103	0.84 [0.34, 2.09]
OAT, 2006	87	1082	84	1084	1.04 [0.78, 1.38]
SWISSI 2, 2007	6	96	22	105	0.30 [0.13, 0.70]
VIAMI, 2012	2	106	3	110	0.69 [0.12, 4.06]
Unrevascularized post–MI studies (**p = 0.04**, Q = 13.52, df = 7, p for heterogeneity = 0.06; I^2 = 48.3%)					0.58 [0.34, 0.98]
Unstable CAD – Multivessel disease following STEMI					
Help–AMI, 2009	1	52	0	17	1.02 [0.04, 23.91]
Politi, 2010	10	130	13	84	0.50 [0.23, 1.08]
Dambrink, 2012	2	79	0	40	2.56 [0.13, 52.14]
PRAMI, 2013	12	234	16	231	0.74 [0.36, 1.53]
CvLPRIT, 2015	4	150	10	146	0.39 [0.12, 1.21]
DANAMI 3, 2015	15	313	11	314	1.37 [0.64, 2.93]
Zhang, 2015	13	215	15	213	0.86 [0.42, 1.76]
Hamza, 2016	1	50	4	50	0.25 [0.03, 2.16]
Compare ACUTE, 2017	4	295	10	590	0.80 [0.25, 2.53]
Complete, 2019	96	2016	106	2025	0.91 [0.70, 1.19]
Multivessel disease following STEMI studies (**p = 0.11**, Q = 7.30, df = 9, p for heterogeneity = 0.61; I^2 = 0.0%)					0.84 [0.69, 1.04]
Unstable CAD – NSTEMI					
TIMI IIIB, 1994	18	740	18	733	0.99 [0.52, 1.89]
FRISC II, 2000	27	1222	48	1234	0.57 [0.36, 0.90]
TRUCS, 2000	3	76	9	72	0.32 [0.09, 1.12]
TACTICS 18, 2001	37	1114	39	1106	0.94 [0.61, 1.47]
RITA 3, 2002	60	895	72	915	0.85 [0.61, 1.19]
VINO, 2002	2	64	9	67	0.23 [0.05, 1.04]
ICTUS, 2005	15	604	15	596	0.99 [0.49, 2.00]
Savonitto, 2012	19	154	22	159	0.89 [0.50, 1.58]
After 80, 2016	57	229	62	228	0.92 [0.67, 1.25]
Sanchis, 2016	22	52	26	54	0.88 [0.58, 1.34]
NSTEMI studies (**p = 0.02**, Q = 8.95, df = 9.00, p for heterogeneity = 0.44; I^2 = 0.0%)					0.84 [0.72, 0.97]
**Unstable CAD studies (p = 0.001, Q = 29.79, df = 27.00, p for heterogeneity = 0.32; I^2 = 0.0%)**					0.84 [0.75, 0.93]
Stable CAD					
ACME 1, 1992	0	105	1	107	0.34 [0.01, 8.24]
MASS 1, 1995	1	72	0	72	3.00 [0.12, 72.44]
ACME 2, 1997	25	166	25	162	0.98 [0.59, 1.63]
RITA 2, 1997	11	504	7	514	1.60 [0.63, 4.10]
ACIP, 1997	2	192	20	366	0.19 [0.05, 0.81]
AVERT, 1999	1	177	1	164	0.93 [0.06, 14.69]
DEFER, 2001	2	90	4	91	0.51 [0.09, 2.69]
TIME, 2001	13	153	6	148	2.10 [0.82, 5.37]
MASS 2, 2004	9	205	3	203	2.97 [0.82, 10.82]
Hambrecht, 2004	0	50	0	51	1.02 [0.02, 50.41]
COURAGE, 2007	85	1149	95	1138	0.89 [0.67, 1.17]
JSAP, 2008	6	188	7	191	0.87 [0.30, 2.54]
FAME 2, 2012	1	447	3	441	0.33 [0.03, 3.15]
**Stable CAD studies (p = 0.68, Q = 14.22, df = 12.00, p for heterogeneity = 0.29; I^2 = 0.0%)**					0.96 [0.77, 1.18]

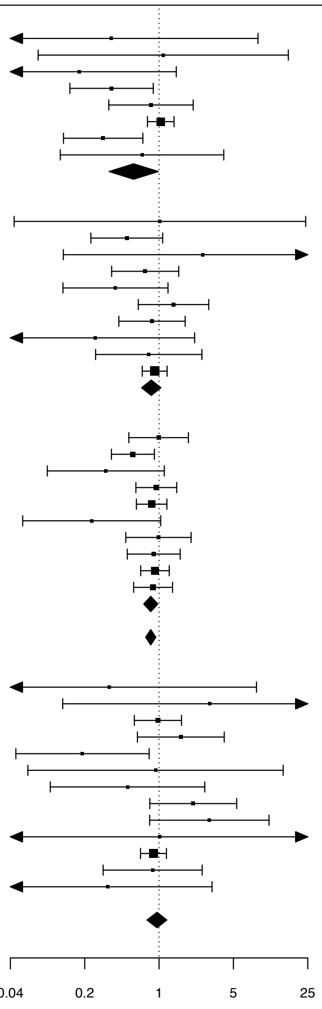

Online Figure 25. Sensitivity analysis for primary outcome of all-cause mortality excluding the COMPARE-ACUTE trial

Study and Year	Active Events	Active N	Control Events	Control N	Relative risk [95% CI]
Unstable CAD – Unrevascularized post–MI					
TOPS, 1992	0	42	1	45	0.36 [0.01, 8.52]
DAKIK, 1998	1	21	1	23	1.10 [0.07, 16.43]
Horie, 1998	1	44	5	39	0.18 [0.02, 1.45]
ALKK, 2003	6	149	17	151	0.36 [0.15, 0.88]
DECOPI, 2004	8	109	9	103	0.84 [0.34, 2.09]
OAT, 2006	87	1082	84	1084	1.04 [0.78, 1.38]
SWISSI 2, 2007	6	96	22	105	0.30 [0.13, 0.70]
VIAMI, 2012	2	106	3	110	0.69 [0.12, 4.06]
Unrevascularized post–MI studies (**p = 0.04**, Q = 13.52, df = 7, p for heterogeneity = 0.06; I^2 = 48.3%)					0.58 [0.34, 0.98]
Unstable CAD – Multivessel disease following STEMI					
Help–AMI, 2009	1	52	0	17	1.02 [0.04, 23.91]
Politi, 2010	10	130	13	84	0.50 [0.23, 1.08]
Dambrink, 2012	2	79	0	40	2.56 [0.13, 52.14]
PRAMI, 2013	12	234	16	231	0.74 [0.36, 1.53]
CvLPRIT, 2015	4	150	10	146	0.39 [0.12, 1.21]
DANAMI 3, 2015	15	313	11	314	1.37 [0.64, 2.93]
Zhang, 2015	13	215	15	213	0.86 [0.42, 1.76]
Hamza, 2016	1	50	4	50	0.25 [0.03, 2.16]
Complete, 2019	96	2016	106	2025	0.91 [0.70, 1.19]
Multivessel disease following STEMI studies (**p = 0.12**, Q = 7.29, df = 8, p for heterogeneity = 0.51; I^2 = 0.0%)					0.85 [0.68, 1.05]
Unstable CAD – NSTEMI					
TIMI IIIB, 1994	18	740	18	733	0.99 [0.52, 1.89]
FRISC II, 2000	27	1222	48	1234	0.57 [0.36, 0.90]
TRUCS, 2000	3	76	9	72	0.32 [0.09, 1.12]
TACTICS 18, 2001	37	1114	39	1106	0.94 [0.61, 1.47]
RITA 3, 2002	60	895	72	915	0.85 [0.61, 1.19]
VINO, 2002	2	64	9	67	0.23 [0.05, 1.04]
ICTUS, 2005	15	604	15	596	0.99 [0.49, 2.00]
Savonitto, 2012	19	154	22	159	0.89 [0.50, 1.58]
After 80, 2016	57	229	62	228	0.92 [0.67, 1.25]
Sanchis, 2016	22	52	26	54	0.88 [0.58, 1.34]
NSTEMI studies (**p = 0.02**, Q = 8.95, df = 9.00, p for heterogeneity = 0.44; I^2 = 0.0%)					0.84 [0.72, 0.97]
**Unstable CAD studies (p = 0.001, Q = 29.78, df = 26.00, p for heterogeneity = 0.28; I^2 = 0.0%)**					0.84 [0.75, 0.93]
Stable CAD					
ACME 1, 1992	0	105	1	107	0.34 [0.01, 8.24]
MASS 1, 1995	1	72	0	72	3.00 [0.12, 72.44]
ACME 2, 1997	25	166	25	162	0.98 [0.59, 1.63]
RITA 2, 1997	11	504	7	514	1.60 [0.63, 4.10]
ACIP, 1997	2	192	20	366	0.19 [0.05, 0.81]
AVERT, 1999	1	177	1	164	0.93 [0.06, 14.69]
DEFER, 2001	2	90	4	91	0.51 [0.09, 2.69]
TIME, 2001	13	153	6	148	2.10 [0.82, 5.37]
MASS 2, 2004	9	205	3	203	2.97 [0.82, 10.82]
Hambrecht, 2004	0	50	0	51	1.02 [0.02, 50.41]
COURAGE, 2007	85	1149	95	1138	0.89 [0.67, 1.17]
JSAP, 2008	6	188	7	191	0.87 [0.30, 2.54]
BARI 2D, 2009	102	798	96	807	1.07 [0.83, 1.39]
FAME 2, 2012	1	447	3	441	0.33 [0.03, 3.15]
**Stable CAD studies (p = 0.98, Q = 14.69, df = 13.00, p for heterogeneity = 0.33; I^2 = 0.0%)**					1.00 [0.85, 1.18]

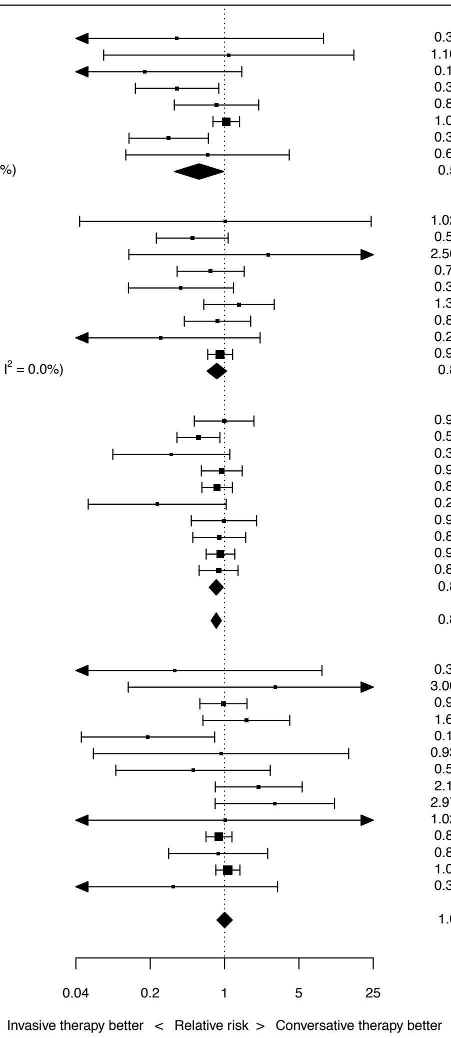

Online Figure 26. Sensitivity analysis for primary outcome of all-cause mortality excluding the COMPLETE trial

Study and Year	Active Events	Active N	Control Events	Control N	Relative risk [95% CI]
Unstable CAD – Unrevascularized post–MI					
TOPS, 1992	0	42	1	45	0.36 [0.01, 8.52]
DAKIK, 1998	1	21	1	23	1.10 [0.07, 16.43]
Horie, 1998	1	44	5	39	0.18 [0.02, 1.45]
ALKK, 2003	6	149	17	151	0.36 [0.15, 0.88]
DECOPI, 2004	8	109	9	103	0.84 [0.34, 2.09]
OAT, 2006	87	1082	84	1084	1.04 [0.78, 1.38]
SWISSI 2, 2007	6	96	22	105	0.30 [0.13, 0.70]
VIAMI, 2012	2	106	3	110	0.69 [0.12, 4.06]
Unrevascularized post–MI studies (**p = 0.04**, Q = 13.52, df = 7, p for heterogeneity = 0.06; I^2 = 48.3%)					0.58 [0.34, 0.98]
Unstable CAD – Multivessel disease following STEMI					
Help–AMI, 2009	1	52	0	17	1.02 [0.04, 23.91]
Politi, 2010	10	130	13	84	0.50 [0.23, 1.08]
Dambrink, 2012	2	79	0	40	2.56 [0.13, 52.14]
PRAMI, 2013	12	234	16	231	0.74 [0.36, 1.53]
CvLPRIT, 2015	4	150	10	146	0.39 [0.12, 1.21]
DANAMI 3, 2015	15	313	11	314	1.37 [0.64, 2.93]
Zhang, 2015	13	215	15	213	0.86 [0.42, 1.76]
Hamza, 2016	1	50	4	50	0.25 [0.03, 2.16]
Compare ACUTE, 2017	4	295	10	590	0.80 [0.25, 2.53]
Multivessel disease following STEMI studies (**p = 0.10**, Q = 6.56, df = 8, p for heterogeneity = 0.58; I^2 = 0.0%)					0.76 [0.54, 1.05]
Unstable CAD – NSTEMI					
TIMI IIIB, 1994	18	740	18	733	0.99 [0.52, 1.89]
FRISC II, 2000	27	1222	48	1234	0.57 [0.36, 0.90]
TRUCS, 2000	3	76	9	72	0.32 [0.09, 1.12]
TACTICS 18, 2001	37	1114	39	1106	0.94 [0.61, 1.47]
RITA 3, 2002	60	895	72	915	0.85 [0.61, 1.19]
VINO, 2002	2	64	9	67	0.23 [0.05, 1.04]
ICTUS, 2005	15	604	15	596	0.99 [0.49, 2.00]
Savonitto, 2012	19	154	22	159	0.89 [0.50, 1.58]
After 80, 2016	57	229	62	228	0.92 [0.67, 1.25]
Sanchis, 2016	22	52	26	54	0.88 [0.58, 1.34]
NSTEMI studies (**p = 0.02**, Q = 8.95, df = 9.00, p for heterogeneity = 0.44; I^2 = 0.0%)					0.84 [0.72, 0.97]
**Unstable CAD studies (p = 0.001, Q = 29.33, df = 26.00, p for heterogeneity = 0.30; I^2 = 8.4%)**					0.80 [0.70, 0.92]
Stable CAD					
ACME 1, 1992	0	105	1	107	0.34 [0.01, 8.24]
MASS 1, 1995	1	72	0	72	3.00 [0.12, 72.44]
ACME 2, 1997	25	166	25	162	0.98 [0.59, 1.63]
RITA 2, 1997	11	504	7	514	1.60 [0.63, 4.10]
ACIP, 1997	2	192	20	366	0.19 [0.05, 0.81]
AVERT, 1999	1	177	1	164	0.93 [0.06, 14.69]
DEFER, 2001	2	90	4	91	0.51 [0.09, 2.69]
TIME, 2001	13	153	6	148	2.10 [0.82, 5.37]
MASS 2, 2004	9	205	3	203	2.97 [0.82, 10.82]
Hambrecht, 2004	0	50	0	51	1.02 [0.02, 50.41]
COURAGE, 2007	85	1149	95	1138	0.89 [0.67, 1.17]
JSAP, 2008	6	188	7	191	0.87 [0.30, 2.54]
BARI 2D, 2009	102	798	96	807	1.07 [0.83, 1.39]
FAME 2, 2012	1	447	3	441	0.33 [0.03, 3.15]
**Stable CAD studies (p = 0.98, Q = 14.69, df = 13.00, p for heterogeneity = 0.33; I^2 = 0.0%)**					1.00 [0.85, 1.18]

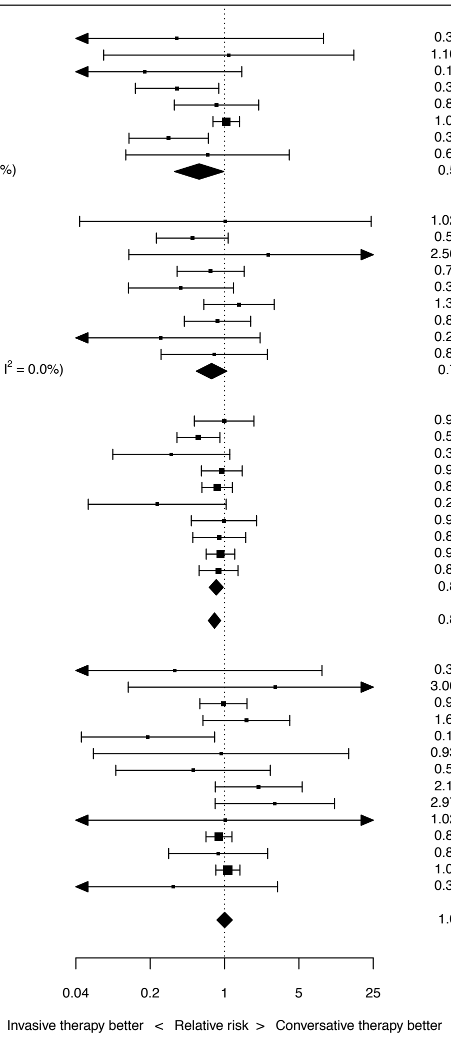

Online Figure 27. Sensitivity analysis for primary outcome of all-cause mortality excluding the COURAGE trial

Study and Year	Active Events	Active N	Control Events	Control N	Relative risk [95% CI]
Unstable CAD – Unrevascularized post–MI					
TOPS, 1992	0	42	1	45	0.36 [0.01, 8.52]
DAKIK, 1998	1	21	1	23	1.10 [0.07, 16.43]
Horie, 1998	1	44	5	39	0.18 [0.02, 1.45]
ALKK, 2003	6	149	17	151	0.36 [0.15, 0.88]
DECOPI, 2004	8	109	9	103	0.84 [0.34, 2.09]
OAT, 2006	87	1082	84	1084	1.04 [0.78, 1.38]
SWISSI 2, 2007	6	96	22	105	0.30 [0.13, 0.70]
VIAMI, 2012	2	106	3	110	0.69 [0.12, 4.06]
Unrevascularized post–MI studies (**p = 0.04**, Q = 13.52, df = 7, p for heterogeneity = 0.06; I^2 = 48.3%)					0.58 [0.34, 0.98]
Unstable CAD – Multivessel disease following STEMI					
Help–AMI, 2009	1	52	0	17	1.02 [0.04, 23.91]
Politi, 2010	10	130	13	84	0.50 [0.23, 1.08]
Dambrink, 2012	2	79	0	40	2.56 [0.13, 52.14]
PRAMI, 2013	12	234	16	231	0.74 [0.36, 1.53]
CvLPRIT, 2015	4	150	10	146	0.39 [0.12, 1.21]
DANAMI 3, 2015	15	313	11	314	1.37 [0.64, 2.93]
Zhang, 2015	13	215	15	213	0.86 [0.42, 1.76]
Hamza, 2016	1	50	4	50	0.25 [0.03, 2.16]
Compare ACUTE, 2017	4	295	10	590	0.80 [0.25, 2.53]
Complete, 2019	96	2016	106	2025	0.91 [0.70, 1.19]
Multivessel disease following STEMI studies (**p = 0.11**, Q = 7.30, df = 9, p for heterogeneity = 0.61; I^2 = 0.0%)					0.84 [0.69, 1.04]
Unstable CAD – NSTEMI					
TIMI IIIB, 1994	18	740	18	733	0.99 [0.52, 1.89]
FRISC II, 2000	27	1222	48	1234	0.57 [0.36, 0.90]
TRUCS, 2000	3	76	9	72	0.32 [0.09, 1.12]
TACTICS 18, 2001	37	1114	39	1106	0.94 [0.61, 1.47]
RITA 3, 2002	60	895	72	915	0.85 [0.61, 1.19]
VINO, 2002	2	64	9	67	0.23 [0.05, 1.04]
ICTUS, 2005	15	604	15	596	0.99 [0.49, 2.00]
Savonitto, 2012	19	154	22	159	0.89 [0.50, 1.58]
After 80, 2016	57	229	62	228	0.92 [0.67, 1.25]
Sanchis, 2016	22	52	26	54	0.88 [0.58, 1.34]
NSTEMI studies (**p = 0.02**, Q = 8.95, df = 9.00, p for heterogeneity = 0.44; I^2 = 0.0%)					0.84 [0.72, 0.97]
**Unstable CAD studies (p = 0.001, Q = 29.79, df = 27.00, p for heterogeneity = 0.32; I^2 = 0.0%)**					0.84 [0.75, 0.93]
Stable CAD					
ACME 1, 1992	0	105	1	107	0.34 [0.01, 8.24]
MASS 1, 1995	1	72	0	72	3.00 [0.12, 72.44]
ACME 2, 1997	25	166	25	162	0.98 [0.59, 1.63]
RITA 2, 1997	11	504	7	514	1.60 [0.63, 4.10]
ACIP, 1997	2	192	20	366	0.19 [0.05, 0.81]
AVERT, 1999	1	177	1	164	0.93 [0.06, 14.69]
DEFER, 2001	2	90	4	91	0.51 [0.09, 2.69]
TIME, 2001	13	153	6	148	2.10 [0.82, 5.37]
MASS 2, 2004	9	205	3	203	2.97 [0.82, 10.82]
Hambrecht, 2004	0	50	0	51	1.02 [0.02, 50.41]
JSAP, 2008	6	188	7	191	0.87 [0.30, 2.54]
BARI 2D, 2009	102	798	96	807	1.07 [0.83, 1.39]
FAME 2, 2012	1	447	3	441	0.33 [0.03, 3.15]
**Stable CAD studies (p = 0.52, Q = 13.56, df = 12.00, p for heterogeneity = 0.33; I^2 = 0.0%)**					1.07 [0.87, 1.31]

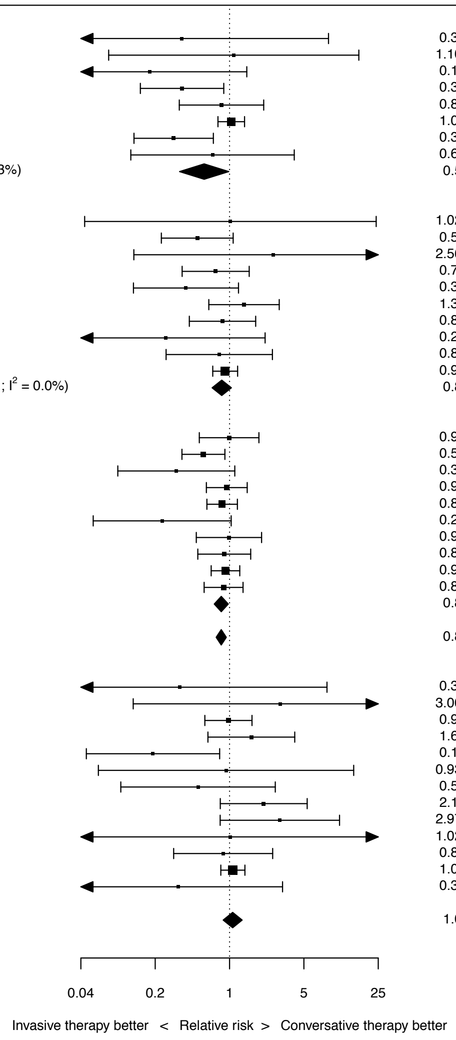

Online Figure 28. Sensitivity analysis for primary outcome of all-cause mortality excluding the CVLPRIT trial

Study and Year	Active Events	N	Control Events	N	Relative risk [95% CI]
Unstable CAD – Unrevascularized post–MI					
TOPS, 1992	0	42	1	45	0.36 [0.01, 8.52]
DAKIK, 1998	1	21	1	23	1.10 [0.07, 16.43]
Horie, 1998	1	44	5	39	0.18 [0.02, 1.45]
ALKK, 2003	6	149	17	151	0.36 [0.15, 0.88]
DECOPI, 2004	8	109	9	103	0.84 [0.34, 2.09]
OAT, 2006	87	1082	84	1084	1.04 [0.78, 1.38]
SWISSI 2, 2007	6	96	22	105	0.30 [0.13, 0.70]
VIAMI, 2012	2	106	3	110	0.69 [0.12, 4.06]
Unrevascularized post–MI studies (**p = 0.04**, Q = 13.52, df = 7, p for heterogeneity = 0.06; I^2 = 48.3%)					0.58 [0.34, 0.98]
Unstable CAD – Multivessel disease following STEMI					
Help–AMI, 2009	1	52	0	17	1.02 [0.04, 23.91]
Politi, 2010	10	130	13	84	0.50 [0.23, 1.08]
Dambrink, 2012	2	79	0	40	2.56 [0.13, 52.14]
PRAMI, 2013	12	234	16	231	0.74 [0.36, 1.53]
DANAMI 3, 2015	15	313	11	314	1.37 [0.64, 2.93]
Zhang, 2015	13	215	15	213	0.86 [0.42, 1.76]
Hamza, 2016	1	50	4	50	0.25 [0.03, 2.16]
Compare ACUTE, 2017	4	295	10	590	0.80 [0.25, 2.53]
Complete, 2019	96	2016	106	2025	0.91 [0.70, 1.19]
Multivessel disease following STEMI studies (**p = 0.19**, Q = 5.45, df = 8, p for heterogeneity = 0.71; I^2 = 0.0%)					0.87 [0.70, 1.07]
Unstable CAD – NSTEMI					
TIMI IIIB, 1994	18	740	18	733	0.99 [0.52, 1.89]
FRISC II, 2000	27	1222	48	1234	0.57 [0.36, 0.90]
TRUCS, 2000	3	76	9	72	0.32 [0.09, 1.12]
TACTICS 18, 2001	37	1114	39	1106	0.94 [0.61, 1.47]
RITA 3, 2002	60	895	72	915	0.85 [0.61, 1.19]
VINO, 2002	2	64	9	67	0.23 [0.05, 1.04]
ICTUS, 2005	15	604	15	596	0.99 [0.49, 2.00]
Savonitto, 2012	19	154	22	159	0.89 [0.50, 1.58]
After 80, 2016	57	229	62	228	0.92 [0.67, 1.25]
Sanchis, 2016	22	52	26	54	0.88 [0.58, 1.34]
NSTEMI studies (**p = 0.02**, Q = 8.95, df = 9.00, p for heterogeneity = 0.44; I^2 = 0.0%)					0.84 [0.72, 0.97]
**Unstable CAD studies (p = 0.002, Q = 28.04, df = 26.00, p for heterogeneity = 0.36; I^2 = 0.0%)**					0.84 [0.75, 0.94]
Stable CAD					
ACME 1, 1992	0	105	1	107	0.34 [0.01, 8.24]
MASS 1, 1995	1	72	0	72	3.00 [0.12, 72.44]
ACME 2, 1997	25	166	25	162	0.98 [0.59, 1.63]
RITA 2, 1997	11	504	7	514	1.60 [0.63, 4.10]
ACIP, 1997	2	192	20	366	0.19 [0.05, 0.81]
AVERT, 1999	1	177	1	164	0.93 [0.06, 14.69]
DEFER, 2001	2	90	4	91	0.51 [0.09, 2.69]
TIME, 2001	13	153	6	148	2.10 [0.82, 5.37]
MASS 2, 2004	9	205	3	203	2.97 [0.82, 10.82]
Hambrecht, 2004	0	50	0	51	1.02 [0.02, 50.41]
COURAGE, 2007	85	1149	95	1138	0.89 [0.67, 1.17]
JSAP, 2008	6	188	7	191	0.87 [0.30, 2.54]
BARI 2D, 2009	102	798	96	807	1.07 [0.83, 1.39]
FAME 2, 2012	1	447	3	441	0.33 [0.03, 3.15]
**Stable CAD studies (p = 0.98, Q = 14.69, df = 13.00, p for heterogeneity = 0.33; I^2 = 0.0%)**					1.00 [0.85, 1.18]

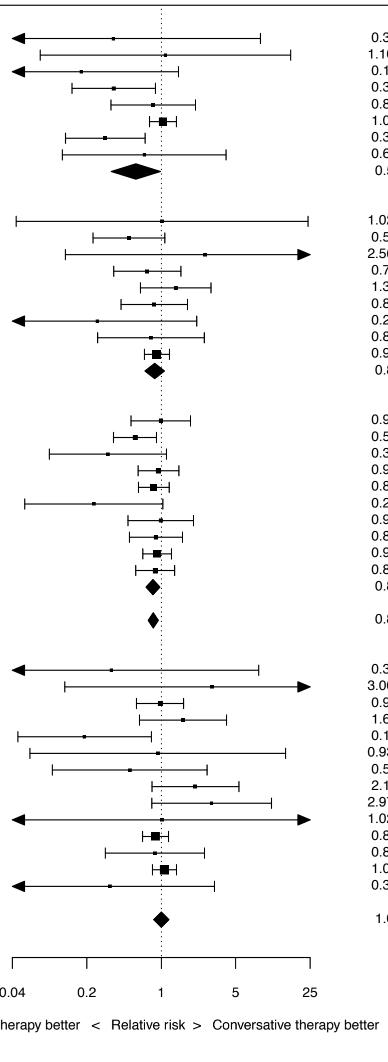

Online Figure 29. Sensitivity analysis for primary outcome of all-cause mortality excluding the DAKIK trial

Study and Year	Active Events	Active N	Control Events	Control N	Relative risk [95% CI]
Unstable CAD – Unrevascularized post–MI					
TOPS, 1992	0	42	1	45	0.36 [0.01, 8.52]
Horie, 1998	1	44	5	39	0.18 [0.02, 1.45]
ALKK, 2003	6	149	17	151	0.36 [0.15, 0.88]
DECOPI, 2004	8	109	9	103	0.84 [0.34, 2.09]
OAT, 2006	87	1082	84	1084	1.04 [0.78, 1.38]
SWISSI 2, 2007	6	96	22	105	0.30 [0.13, 0.70]
VIAMI, 2012	2	106	3	110	0.69 [0.12, 4.06]
Unrevascularized post–MI studies (**p = 0.04**, Q = 13.48, df = 6, p for heterogeneity = 0.04; I^2 = 53.5%)					0.56 [0.32, 0.98]
Unstable CAD – Multivessel disease following STEMI					
Help–AMI, 2009	1	52	0	17	1.02 [0.04, 23.91]
Politi, 2010	10	130	13	84	0.50 [0.23, 1.08]
Dambrink, 2012	2	79	0	40	2.56 [0.13, 52.14]
PRAMI, 2013	12	234	16	231	0.74 [0.36, 1.53]
CvLPRIT, 2015	4	150	10	146	0.39 [0.12, 1.21]
DANAMI 3, 2015	15	313	11	314	1.37 [0.64, 2.93]
Zhang, 2015	13	215	15	213	0.86 [0.42, 1.76]
Hamza, 2016	1	50	4	50	0.25 [0.03, 2.16]
Compare ACUTE, 2017	4	295	10	590	0.80 [0.25, 2.53]
Complete, 2019	96	2016	106	2025	0.91 [0.70, 1.19]
Multivessel disease following STEMI studies (**p = 0.11**, Q = 7.30, df = 9, p for heterogeneity = 0.61; I^2 = 0.0%)					0.84 [0.69, 1.04]
Unstable CAD – NSTEMI					
TIMI IIIB, 1994	18	740	18	733	0.99 [0.52, 1.89]
FRISC II, 2000	27	1222	48	1234	0.57 [0.36, 0.90]
TRUCS, 2000	3	76	9	72	0.32 [0.09, 1.12]
TACTICS 18, 2001	37	1114	39	1106	0.94 [0.61, 1.47]
RITA 3, 2002	60	895	72	915	0.85 [0.61, 1.19]
VINO, 2002	2	64	9	67	0.23 [0.05, 1.04]
ICTUS, 2005	15	604	15	596	0.99 [0.49, 2.00]
Savonitto, 2012	19	154	22	159	0.89 [0.50, 1.58]
After 80, 2016	57	229	62	228	0.92 [0.67, 1.25]
Sanchis, 2016	22	52	26	54	0.88 [0.58, 1.34]
NSTEMI studies (**p = 0.02**, Q = 8.95, df = 9.00, p for heterogeneity = 0.44; I^2 = 0.0%)					0.84 [0.72, 0.97]
**Unstable CAD studies (p = 0.001, Q = 29.75, df = 26.00, p for heterogeneity = 0.28; I^2 = 0.0%)**					0.83 [0.75, 0.93]
Stable CAD					
ACME 1, 1992	0	105	1	107	0.34 [0.01, 8.24]
MASS 1, 1995	1	72	0	72	3.00 [0.12, 72.44]
ACME 2, 1997	25	166	25	162	0.98 [0.59, 1.63]
RITA 2, 1997	11	504	7	514	1.60 [0.63, 4.10]
ACIP, 1997	2	192	20	366	0.19 [0.05, 0.81]
AVERT, 1999	1	177	1	164	0.93 [0.06, 14.69]
DEFER, 2001	2	90	4	91	0.51 [0.09, 2.69]
TIME, 2001	13	153	6	148	2.10 [0.82, 5.37]
MASS 2, 2004	9	205	3	203	2.97 [0.82, 10.82]
Hambrecht, 2004	0	50	0	51	1.02 [0.02, 50.41]
COURAGE, 2007	85	1149	95	1138	0.89 [0.67, 1.17]
JSAP, 2008	6	188	7	191	0.87 [0.30, 2.54]
BARI 2D, 2009	102	798	96	807	1.07 [0.83, 1.39]
FAME 2, 2012	1	447	3	441	0.33 [0.03, 3.15]
**Stable CAD studies (p = 0.98, Q = 14.69, df = 13.00, p for heterogeneity = 0.33; I^2 = 0.0%)**					1.00 [0.85, 1.18]

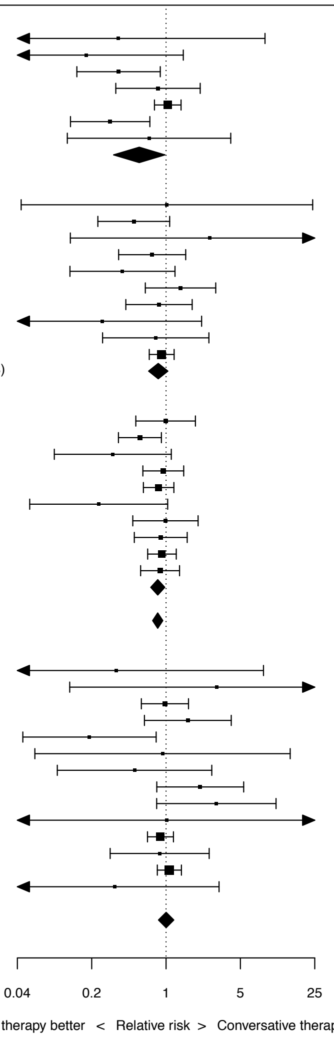

Online Figure 30. Sensitivity analysis for primary outcome of all-cause mortality excluding the Dambrink trial

Study and Year	Active Events	Active N	Control Events	Control N	Relative risk [95% CI]
Unstable CAD – Unrevascularized post–MI					
TOPS, 1992	0	42	1	45	0.36 [0.01, 8.52]
DAKIK, 1998	1	21	1	23	1.10 [0.07, 16.43]
Horie, 1998	1	44	5	39	0.18 [0.02, 1.45]
ALKK, 2003	6	149	17	151	0.36 [0.15, 0.88]
DECOPI, 2004	8	109	9	103	0.84 [0.34, 2.09]
OAT, 2006	87	1082	84	1084	1.04 [0.78, 1.38]
SWISSI 2, 2007	6	96	22	105	0.30 [0.13, 0.70]
VIAMI, 2012	2	106	3	110	0.69 [0.12, 4.06]
Unrevascularized post–MI studies (**p = 0.04**, Q = 13.52, df = 7, p for heterogeneity = 0.06; I^2 = 48.3%)					0.58 [0.34, 0.98]
Unstable CAD – Multivessel disease following STEMI					
Help–AMI, 2009	1	52	0	17	1.02 [0.04, 23.91]
Politi, 2010	10	130	13	84	0.50 [0.23, 1.08]
PRAMI, 2013	12	234	16	231	0.74 [0.36, 1.53]
CvLPRIT, 2015	4	150	10	146	0.39 [0.12, 1.21]
DANAMI 3, 2015	15	313	11	314	1.37 [0.64, 2.93]
Zhang, 2015	13	215	15	213	0.86 [0.42, 1.76]
Hamza, 2016	1	50	4	50	0.25 [0.03, 2.16]
Compare ACUTE, 2017	4	295	10	590	0.80 [0.25, 2.53]
Complete, 2019	96	2016	106	2025	0.91 [0.70, 1.19]
Multivessel disease following STEMI studies (**p = 0.10**, Q = 6.77, df = 8, p for heterogeneity = 0.56; I^2 = 0.0%)					0.84 [0.68, 1.04]
Unstable CAD – NSTEMI					
TIMI IIIB, 1994	18	740	18	733	0.99 [0.52, 1.89]
FRISC II, 2000	27	1222	48	1234	0.57 [0.36, 0.90]
TRUCS, 2000	3	76	9	72	0.32 [0.09, 1.12]
TACTICS 18, 2001	37	1114	39	1106	0.94 [0.61, 1.47]
RITA 3, 2002	60	895	72	915	0.85 [0.61, 1.19]
VINO, 2002	2	64	9	67	0.23 [0.05, 1.04]
ICTUS, 2005	15	604	15	596	0.99 [0.49, 2.00]
Savonitto, 2012	19	154	22	159	0.89 [0.50, 1.58]
After 80, 2016	57	229	62	228	0.92 [0.67, 1.25]
Sanchis, 2016	22	52	26	54	0.88 [0.58, 1.34]
NSTEMI studies (**p = 0.02**, Q = 8.95, df = 9.00, p for heterogeneity = 0.44; I^2 = 0.0%)					0.84 [0.72, 0.97]
**Unstable CAD studies (p = 0.001, Q = 29.26, df = 26.00, p for heterogeneity = 0.30; I^2 = 0.0%)**					0.83 [0.75, 0.93]
Stable CAD					
ACME 1, 1992	0	105	1	107	0.34 [0.01, 8.24]
MASS 1, 1995	1	72	0	72	3.00 [0.12, 72.44]
ACME 2, 1997	25	166	25	162	0.98 [0.59, 1.63]
RITA 2, 1997	11	504	7	514	1.60 [0.63, 4.10]
ACIP, 1997	2	192	20	366	0.19 [0.05, 0.81]
AVERT, 1999	1	177	1	164	0.93 [0.06, 14.69]
DEFER, 2001	2	90	4	91	0.51 [0.09, 2.69]
TIME, 2001	13	153	6	148	2.10 [0.82, 5.37]
MASS 2, 2004	9	205	3	203	2.97 [0.82, 10.82]
Hambrecht, 2004	0	50	0	51	1.02 [0.02, 50.41]
COURAGE, 2007	85	1149	95	1138	0.89 [0.67, 1.17]
JSAP, 2008	6	188	7	191	0.87 [0.30, 2.54]
BARI 2D, 2009	102	798	96	807	1.07 [0.83, 1.39]
FAME 2, 2012	1	447	3	441	0.33 [0.03, 3.15]
**Stable CAD studies (p = 0.98, Q = 14.69, df = 13.00, p for heterogeneity = 0.33; I^2 = 0.0%)**					1.00 [0.85, 1.18]

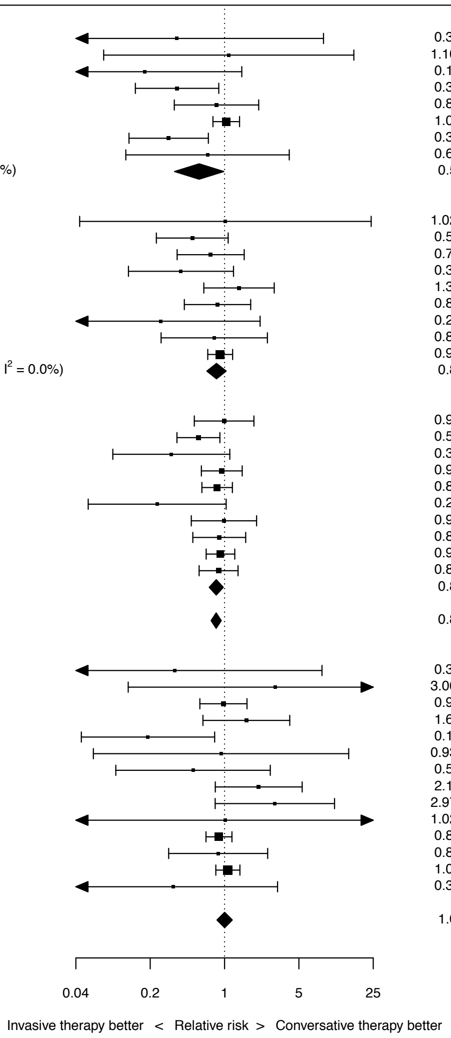

Online Figure 31. Sensitivity analysis for primary outcome of all-cause mortality excluding the DANAMI 3 trial

Study and Year	Active Events	Active N	Control Events	Control N	Relative risk [95% CI]
Unstable CAD – Unrevascularized post–MI					
TOPS, 1992	0	42	1	45	0.36 [0.01, 8.52]
DAKIK, 1998	1	21	1	23	1.10 [0.07, 16.43]
Horie, 1998	1	44	5	39	0.18 [0.02, 1.45]
ALKK, 2003	6	149	17	151	0.36 [0.15, 0.88]
DECOPI, 2004	8	109	9	103	0.84 [0.34, 2.09]
OAT, 2006	87	1082	84	1084	1.04 [0.78, 1.38]
SWISSI 2, 2007	6	96	22	105	0.30 [0.13, 0.70]
VIAMI, 2012	2	106	3	110	0.69 [0.12, 4.06]
Unrevascularized post–MI studies (**p = 0.04**, Q = 13.52, df = 7, p for heterogeneity = 0.06; I^2 = 48.3%)					0.58 [0.34, 0.98]
Unstable CAD – Multivessel disease following STEMI					
Help–AMI, 2009	1	52	0	17	1.02 [0.04, 23.91]
Politi, 2010	10	130	13	84	0.50 [0.23, 1.08]
Dambrink, 2012	2	79	0	40	2.56 [0.13, 52.14]
PRAMI, 2013	12	234	16	231	0.74 [0.36, 1.53]
CvLPRIT, 2015	4	150	10	146	0.39 [0.12, 1.21]
Zhang, 2015	13	215	15	213	0.86 [0.42, 1.76]
Hamza, 2016	1	50	4	50	0.25 [0.03, 2.16]
Compare ACUTE, 2017	4	295	10	590	0.80 [0.25, 2.53]
Complete, 2019	96	2016	106	2025	0.91 [0.70, 1.19]
Multivessel disease following STEMI studies (**p = 0.06**, Q = 5.63, df = 8, p for heterogeneity = 0.69; I^2 = 3.9%)					0.79 [0.62, 1.01]
Unstable CAD – NSTEMI					
TIMI IIIB, 1994	18	740	18	733	0.99 [0.52, 1.89]
FRISC II, 2000	27	1222	48	1234	0.57 [0.36, 0.90]
TRUCS, 2000	3	76	9	72	0.32 [0.09, 1.12]
TACTICS 18, 2001	37	1114	39	1106	0.94 [0.61, 1.47]
RITA 3, 2002	60	895	72	915	0.85 [0.61, 1.19]
VINO, 2002	2	64	9	67	0.23 [0.05, 1.04]
ICTUS, 2005	15	604	15	596	0.99 [0.49, 2.00]
Savonitto, 2012	19	154	22	159	0.89 [0.50, 1.58]
After 80, 2016	57	229	62	228	0.92 [0.67, 1.25]
Sanchis, 2016	22	52	26	54	0.88 [0.58, 1.34]
NSTEMI studies (**p = 0.02**, Q = 8.95, df = 9.00, p for heterogeneity = 0.44; I^2 = 0.0%)					0.84 [0.72, 0.97]
**Unstable CAD studies (p = 0.001, Q = 28.15, df = 26.00, p for heterogeneity = 0.35; I^2 = 1.0%)**					0.82 [0.74, 0.92]
Stable CAD					
ACME 1, 1992	0	105	1	107	0.34 [0.01, 8.24]
MASS 1, 1995	1	72	0	72	3.00 [0.12, 72.44]
ACME 2, 1997	25	166	25	162	0.98 [0.59, 1.63]
RITA 2, 1997	11	504	7	514	1.60 [0.63, 4.10]
ACIP, 1997	2	192	20	366	0.19 [0.05, 0.81]
AVERT, 1999	1	177	1	164	0.93 [0.06, 14.69]
DEFER, 2001	2	90	4	91	0.51 [0.09, 2.69]
TIME, 2001	13	153	6	148	2.10 [0.82, 5.37]
MASS 2, 2004	9	205	3	203	2.97 [0.82, 10.82]
Hambrecht, 2004	0	50	0	51	1.02 [0.02, 50.41]
COURAGE, 2007	85	1149	95	1138	0.89 [0.67, 1.17]
JSAP, 2008	6	188	7	191	0.87 [0.30, 2.54]
BARI 2D, 2009	102	798	96	807	1.07 [0.83, 1.39]
FAME 2, 2012	1	447	3	441	0.33 [0.03, 3.15]
**Stable CAD studies (p = 0.98, Q = 14.69, df = 13.00, p for heterogeneity = 0.33; I^2 = 0.0%)**					1.00 [0.85, 1.18]

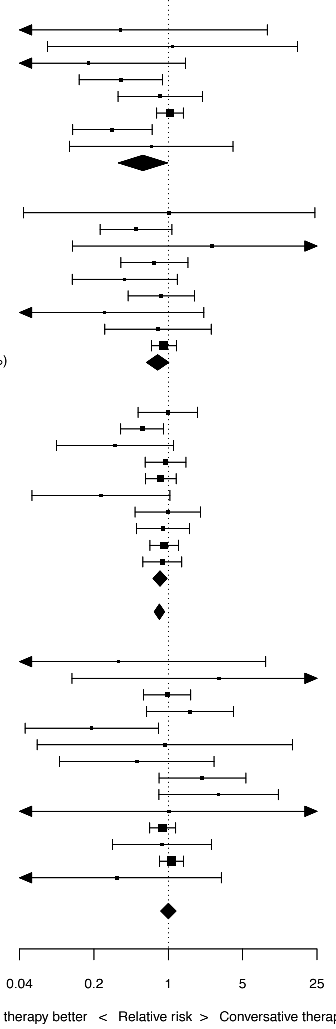

Online Figure 32. Sensitivity analysis for primary outcome of all-cause mortality excluding the DECOPI trial

Study and Year	Active Events	Active N	Control Events	Control N	Relative risk [95% CI]
Unstable CAD – Unrevascularized post–MI					
TOPS, 1992	0	42	1	45	0.36 [0.01, 8.52]
DAKIK, 1998	1	21	1	23	1.10 [0.07, 16.43]
Horie, 1998	1	44	5	39	0.18 [0.02, 1.45]
ALKK, 2003	6	149	17	151	0.36 [0.15, 0.88]
OAT, 2006	87	1082	84	1084	1.04 [0.78, 1.38]
SWISSI 2, 2007	6	96	22	105	0.30 [0.13, 0.70]
VIAMI, 2012	2	106	3	110	0.69 [0.12, 4.06]
Unrevascularized post–MI studies (**p = 0.04**, Q = 13.52, df = 6, p for heterogeneity = 0.04; I^2 = 51.5%)					0.52 [0.28, 0.98]
Unstable CAD – Multivessel disease following STEMI					
Help–AMI, 2009	1	52	0	17	1.02 [0.04, 23.91]
Politi, 2010	10	130	13	84	0.50 [0.23, 1.08]
Dambrink, 2012	2	79	0	40	2.56 [0.13, 52.14]
PRAMI, 2013	12	234	16	231	0.74 [0.36, 1.53]
CvLPRIT, 2015	4	150	10	146	0.39 [0.12, 1.21]
DANAMI 3, 2015	15	313	11	314	1.37 [0.64, 2.93]
Zhang, 2015	13	215	15	213	0.86 [0.42, 1.76]
Hamza, 2016	1	50	4	50	0.25 [0.03, 2.16]
Compare ACUTE, 2017	4	295	10	590	0.80 [0.25, 2.53]
Complete, 2019	96	2016	106	2025	0.91 [0.70, 1.19]
Multivessel disease following STEMI studies (**p = 0.11**, Q = 7.30, df = 9, p for heterogeneity = 0.61; I^2 = 0.0%)					0.84 [0.69, 1.04]
Unstable CAD – NSTEMI					
TIMI IIIB, 1994	18	740	18	733	0.99 [0.52, 1.89]
FRISC II, 2000	27	1222	48	1234	0.57 [0.36, 0.90]
TRUCS, 2000	3	76	9	72	0.32 [0.09, 1.12]
TACTICS 18, 2001	37	1114	39	1106	0.94 [0.61, 1.47]
RITA 3, 2002	60	895	72	915	0.85 [0.61, 1.19]
VINO, 2002	2	64	9	67	0.23 [0.05, 1.04]
ICTUS, 2005	15	604	15	596	0.99 [0.49, 2.00]
Savonitto, 2012	19	154	22	159	0.89 [0.50, 1.58]
After 80, 2016	57	229	62	228	0.92 [0.67, 1.25]
Sanchis, 2016	22	52	26	54	0.88 [0.58, 1.34]
NSTEMI studies (**p = 0.02**, Q = 8.95, df = 9.00, p for heterogeneity = 0.44; I^2 = 0.0%)					0.84 [0.72, 0.97]
**Unstable CAD studies (p = 0.001, Q = 29.79, df = 26.00, p for heterogeneity = 0.28; I^2 = 0.0%)**					0.84 [0.75, 0.93]
Stable CAD					
ACME 1, 1992	0	105	1	107	0.34 [0.01, 8.24]
MASS 1, 1995	1	72	0	72	3.00 [0.12, 72.44]
ACME 2, 1997	25	166	25	162	0.98 [0.59, 1.63]
RITA 2, 1997	11	504	7	514	1.60 [0.63, 4.10]
ACIP, 1997	2	192	20	366	0.19 [0.05, 0.81]
AVERT, 1999	1	177	1	164	0.93 [0.06, 14.69]
DEFER, 2001	2	90	4	91	0.51 [0.09, 2.69]
TIME, 2001	13	153	6	148	2.10 [0.82, 5.37]
MASS 2, 2004	9	205	3	203	2.97 [0.82, 10.82]
Hambrecht, 2004	0	50	0	51	1.02 [0.02, 50.41]
COURAGE, 2007	85	1149	95	1138	0.89 [0.67, 1.17]
JSAP, 2008	6	188	7	191	0.87 [0.30, 2.54]
BARI 2D, 2009	102	798	96	807	1.07 [0.83, 1.39]
FAME 2, 2012	1	447	3	441	0.33 [0.03, 3.15]
**Stable CAD studies (p = 0.98, Q = 14.69, df = 13.00, p for heterogeneity = 0.33; I^2 = 0.0%)**					1.00 [0.85, 1.18]

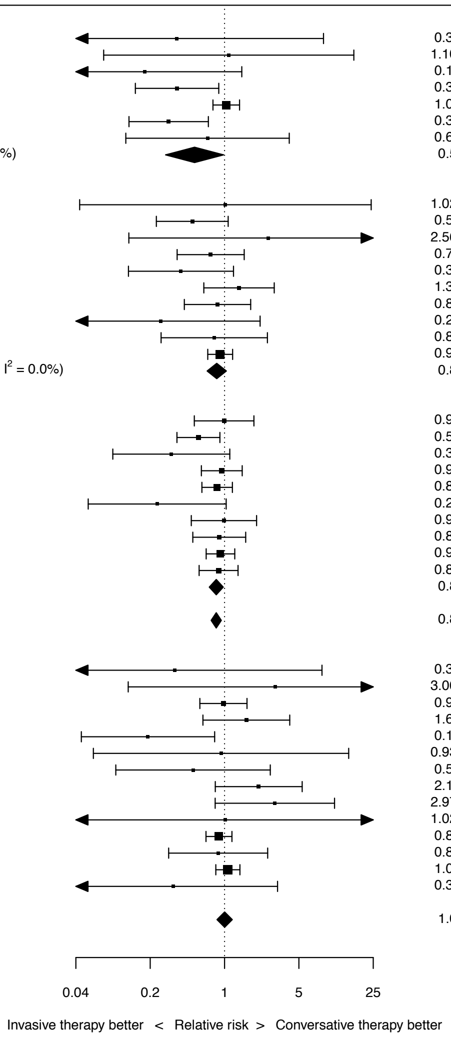

Online Figure 33. Sensitivity analysis for primary outcome of all-cause mortality excluding the DEFER trial

Study and Year	Active Events	N	Control Events	N	Relative risk [95% CI]
Unstable CAD – Unrevascularized post–MI					
TOPS, 1992	0	42	1	45	0.36 [0.01, 8.52]
DAKIK, 1998	1	21	1	23	1.10 [0.07, 16.43]
Horie, 1998	1	44	5	39	0.18 [0.02, 1.45]
ALKK, 2003	6	149	17	151	0.36 [0.15, 0.88]
DECOPI, 2004	8	109	9	103	0.84 [0.34, 2.09]
OAT, 2006	87	1082	84	1084	1.04 [0.78, 1.38]
SWISSI 2, 2007	6	96	22	105	0.30 [0.13, 0.70]
VIAMI, 2012	2	106	3	110	0.69 [0.12, 4.06]
Unrevascularized post–MI studies (**p = 0.04**, Q = 13.52, df = 7, p for heterogeneity = 0.06; I^2 = 48.3%)					0.58 [0.34, 0.98]
Unstable CAD – Multivessel disease following STEMI					
Help–AMI, 2009	1	52	0	17	1.02 [0.04, 23.91]
Politi, 2010	10	130	13	84	0.50 [0.23, 1.08]
Dambrink, 2012	2	79	0	40	2.56 [0.13, 52.14]
PRAMI, 2013	12	234	16	231	0.74 [0.36, 1.53]
CvLPRIT, 2015	4	150	10	146	0.39 [0.12, 1.21]
DANAMI 3, 2015	15	313	11	314	1.37 [0.64, 2.93]
Zhang, 2015	13	215	15	213	0.86 [0.42, 1.76]
Hamza, 2016	1	50	4	50	0.25 [0.03, 2.16]
Compare ACUTE, 2017	4	295	10	590	0.80 [0.25, 2.53]
Complete, 2019	96	2016	106	2025	0.91 [0.70, 1.19]
Multivessel disease following STEMI studies (**p = 0.11**, Q = 7.30, df = 9, p for heterogeneity = 0.61; I^2 = 0.0%)					0.84 [0.69, 1.04]
Unstable CAD – NSTEMI					
TIMI IIIB, 1994	18	740	18	733	0.99 [0.52, 1.89]
FRISC II, 2000	27	1222	48	1234	0.57 [0.36, 0.90]
TRUCS, 2000	3	76	9	72	0.32 [0.09, 1.12]
TACTICS 18, 2001	37	1114	39	1106	0.94 [0.61, 1.47]
RITA 3, 2002	60	895	72	915	0.85 [0.61, 1.19]
VINO, 2002	2	64	9	67	0.23 [0.05, 1.04]
ICTUS, 2005	15	604	15	596	0.99 [0.49, 2.00]
Savonitto, 2012	19	154	22	159	0.89 [0.50, 1.58]
After 80, 2016	57	229	62	228	0.92 [0.67, 1.25]
Sanchis, 2016	22	52	26	54	0.88 [0.58, 1.34]
NSTEMI studies (**p = 0.02**, Q = 8.95, df = 9.00, p for heterogeneity = 0.44; I^2 = 0.0%)					0.84 [0.72, 0.97]
**Unstable CAD studies (p = 0.001, Q = 29.79, df = 27.00, p for heterogeneity = 0.32; I^2 = 0.0%)**					0.84 [0.75, 0.93]
Stable CAD					
ACME 1, 1992	0	105	1	107	0.34 [0.01, 8.24]
MASS 1, 1995	1	72	0	72	3.00 [0.12, 72.44]
ACME 2, 1997	25	166	25	162	0.98 [0.59, 1.63]
RITA 2, 1997	11	504	7	514	1.60 [0.63, 4.10]
ACIP, 1997	2	192	20	366	0.19 [0.05, 0.81]
AVERT, 1999	1	177	1	164	0.93 [0.06, 14.69]
TIME, 2001	13	153	6	148	2.10 [0.82, 5.37]
MASS 2, 2004	9	205	3	203	2.97 [0.82, 10.82]
Hambrecht, 2004	0	50	0	51	1.02 [0.02, 50.41]
COURAGE, 2007	85	1149	95	1138	0.89 [0.67, 1.17]
JSAP, 2008	6	188	7	191	0.87 [0.30, 2.54]
BARI 2D, 2009	102	798	96	807	1.07 [0.83, 1.39]
FAME 2, 2012	1	447	3	441	0.33 [0.03, 3.15]
**Stable CAD studies (p = 0.92, Q = 14.04, df = 12.00, p for heterogeneity = 0.30; I^2 = 0.0%)**					1.01 [0.85, 1.19]

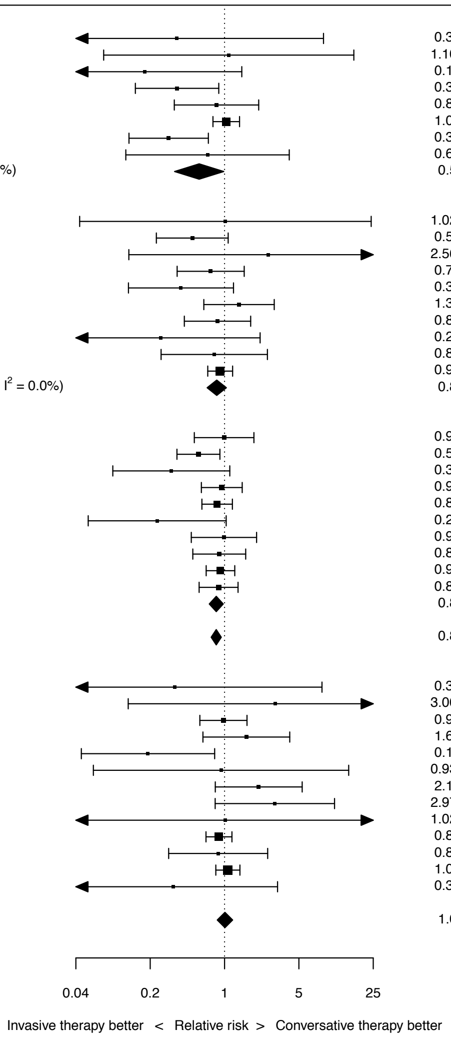

Online Figure 34. Sensitivity analysis for primary outcome of all-cause mortality excluding the FAME 2 trial

Study and Year	Active Events	Active N	Control Events	Control N	Relative risk [95% CI]
Unstable CAD – Unrevascularized post–MI					
TOPS, 1992	0	42	1	45	0.36 [0.01, 8.52]
DAKIK, 1998	1	21	1	23	1.10 [0.07, 16.43]
Horie, 1998	1	44	5	39	0.18 [0.02, 1.45]
ALKK, 2003	6	149	17	151	0.36 [0.15, 0.88]
DECOPI, 2004	8	109	9	103	0.84 [0.34, 2.09]
OAT, 2006	87	1082	84	1084	1.04 [0.78, 1.38]
SWISSI 2, 2007	6	96	22	105	0.30 [0.13, 0.70]
VIAMI, 2012	2	106	3	110	0.69 [0.12, 4.06]
Unrevascularized post–MI studies (**p = 0.04**, Q = 13.52, df = 7, p for heterogeneity = 0.06; I^2 = 48.3%)					0.58 [0.34, 0.98]
Unstable CAD – Multivessel disease following STEMI					
Help–AMI, 2009	1	52	0	17	1.02 [0.04, 23.91]
Politi, 2010	10	130	13	84	0.50 [0.23, 1.08]
Dambrink, 2012	2	79	0	40	2.56 [0.13, 52.14]
PRAMI, 2013	12	234	16	231	0.74 [0.36, 1.53]
CvLPRIT, 2015	4	150	10	146	0.39 [0.12, 1.21]
DANAMI 3, 2015	15	313	11	314	1.37 [0.64, 2.93]
Zhang, 2015	13	215	15	213	0.86 [0.42, 1.76]
Hamza, 2016	1	50	4	50	0.25 [0.03, 2.16]
Compare ACUTE, 2017	4	295	10	590	0.80 [0.25, 2.53]
Complete, 2019	96	2016	106	2025	0.91 [0.70, 1.19]
Multivessel disease following STEMI studies (**p = 0.11**, Q = 7.30, df = 9, p for heterogeneity = 0.61; I^2 = 0.0%)					0.84 [0.69, 1.04]
Unstable CAD – NSTEMI					
TIMI IIIB, 1994	18	740	18	733	0.99 [0.52, 1.89]
FRISC II, 2000	27	1222	48	1234	0.57 [0.36, 0.90]
TRUCS, 2000	3	76	9	72	0.32 [0.09, 1.12]
TACTICS 18, 2001	37	1114	39	1106	0.94 [0.61, 1.47]
RITA 3, 2002	60	895	72	915	0.85 [0.61, 1.19]
VINO, 2002	2	64	9	67	0.23 [0.05, 1.04]
ICTUS, 2005	15	604	15	596	0.99 [0.49, 2.00]
Savonitto, 2012	19	154	22	159	0.89 [0.50, 1.58]
After 80, 2016	57	229	62	228	0.92 [0.67, 1.25]
Sanchis, 2016	22	52	26	54	0.88 [0.58, 1.34]
NSTEMI studies (**p = 0.02**, Q = 8.95, df = 9.00, p for heterogeneity = 0.44; I^2 = 0.0%)					0.84 [0.72, 0.97]
**Unstable CAD studies (p = 0.001, Q = 29.79, df = 27.00, p for heterogeneity = 0.32; I^2 = 0.0%)**					0.84 [0.75, 0.93]
Stable CAD					
ACME 1, 1992	0	105	1	107	0.34 [0.01, 8.24]
MASS 1, 1995	1	72	0	72	3.00 [0.12, 72.44]
ACME 2, 1997	25	166	25	162	0.98 [0.59, 1.63]
RITA 2, 1997	11	504	7	514	1.60 [0.63, 4.10]
ACIP, 1997	2	192	20	366	0.19 [0.05, 0.81]
AVERT, 1999	1	177	1	164	0.93 [0.06, 14.69]
DEFER, 2001	2	90	4	91	0.51 [0.09, 2.69]
TIME, 2001	13	153	6	148	2.10 [0.82, 5.37]
MASS 2, 2004	9	205	3	203	2.97 [0.82, 10.82]
Hambrecht, 2004	0	50	0	51	1.02 [0.02, 50.41]
COURAGE, 2007	85	1149	95	1138	0.89 [0.67, 1.17]
JSAP, 2008	6	188	7	191	0.87 [0.30, 2.54]
BARI 2D, 2009	102	798	96	807	1.07 [0.83, 1.39]
**Stable CAD studies (p = 0.93, Q = 13.75, df = 12.00, p for heterogeneity = 0.32; I^2 = 0.0%)**					1.01 [0.85, 1.19]

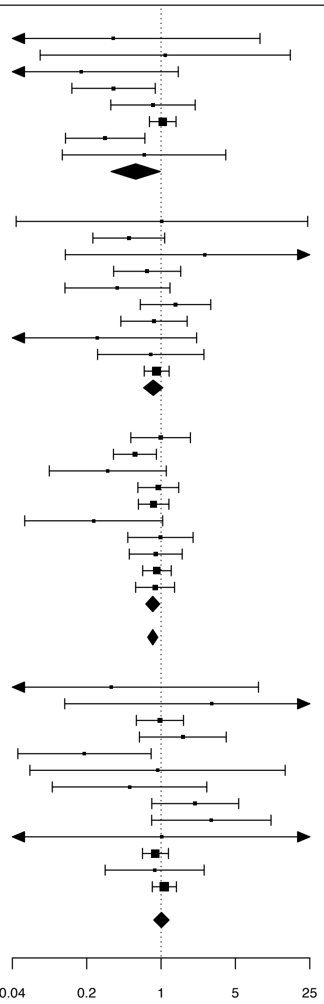

Invasive therapy better < Relative risk > Conversative therapy better

Online Figure 35. Sensitivity analysis for primary outcome of all-cause mortality excluding the FRISC II trial

Study and Year	Active Events	Active N	Control Events	Control N	Relative risk [95% CI]
Unstable CAD – Unrevascularized post–MI					
TOPS, 1992	0	42	1	45	0.36 [0.01, 8.52]
DAKIK, 1998	1	21	1	23	1.10 [0.07, 16.43]
Horie, 1998	1	44	5	39	0.18 [0.02, 1.45]
ALKK, 2003	6	149	17	151	0.36 [0.15, 0.88]
DECOPI, 2004	8	109	9	103	0.84 [0.34, 2.09]
OAT, 2006	87	1082	84	1084	1.04 [0.78, 1.38]
SWISSI 2, 2007	6	96	22	105	0.30 [0.13, 0.70]
VIAMI, 2012	2	106	3	110	0.69 [0.12, 4.06]
Unrevascularized post–MI studies (**p = 0.04**, Q = 13.52, df = 7, p for heterogeneity = 0.06; I^2 = 48.3%)					0.58 [0.34, 0.98]
Unstable CAD – Multivessel disease following STEMI					
Help–AMI, 2009	1	52	0	17	1.02 [0.04, 23.91]
Politi, 2010	10	130	13	84	0.50 [0.23, 1.08]
Dambrink, 2012	2	79	0	40	2.56 [0.13, 52.14]
PRAMI, 2013	12	234	16	231	0.74 [0.36, 1.53]
CvLPRIT, 2015	4	150	10	146	0.39 [0.12, 1.21]
DANAMI 3, 2015	15	313	11	314	1.37 [0.64, 2.93]
Zhang, 2015	13	215	15	213	0.86 [0.42, 1.76]
Hamza, 2016	1	50	4	50	0.25 [0.03, 2.16]
Compare ACUTE, 2017	4	295	10	590	0.80 [0.25, 2.53]
Complete, 2019	96	2016	106	2025	0.91 [0.70, 1.19]
Multivessel disease following STEMI studies (**p = 0.11**, Q = 7.30, df = 9, p for heterogeneity = 0.61; I^2 = 0.0%)					0.84 [0.69, 1.04]
Unstable CAD – NSTEMI					
TIMI IIIB, 1994	18	740	18	733	0.99 [0.52, 1.89]
TRUCS, 2000	3	76	9	72	0.32 [0.09, 1.12]
TACTICS 18, 2001	37	1114	39	1106	0.94 [0.61, 1.47]
RITA 3, 2002	60	895	72	915	0.85 [0.61, 1.19]
VINO, 2002	2	64	9	67	0.23 [0.05, 1.04]
ICTUS, 2005	15	604	15	596	0.99 [0.49, 2.00]
Savonitto, 2012	19	154	22	159	0.89 [0.50, 1.58]
After 80, 2016	57	229	62	228	0.92 [0.67, 1.25]
Sanchis, 2016	22	52	26	54	0.88 [0.58, 1.34]
NSTEMI studies (**p = 0.10**, Q = 5.98, df = 8.00, p for heterogeneity = 0.65; I^2 = 0.0%)					0.87 [0.75, 1.03]
**Unstable CAD studies (p = 0.006, Q = 26.99, df = 26.00, p for heterogeneity = 0.41; I^2 = 0.0%)**					0.85 [0.76, 0.96]
Stable CAD					
ACME 1, 1992	0	105	1	107	0.34 [0.01, 8.24]
MASS 1, 1995	1	72	0	72	3.00 [0.12, 72.44]
ACME 2, 1997	25	166	25	162	0.98 [0.59, 1.63]
RITA 2, 1997	11	504	7	514	1.60 [0.63, 4.10]
ACIP, 1997	2	192	20	366	0.19 [0.05, 0.81]
AVERT, 1999	1	177	1	164	0.93 [0.06, 14.69]
DEFER, 2001	2	90	4	91	0.51 [0.09, 2.69]
TIME, 2001	13	153	6	148	2.10 [0.82, 5.37]
MASS 2, 2004	9	205	3	203	2.97 [0.82, 10.82]
Hambrecht, 2004	0	50	0	51	1.02 [0.02, 50.41]
COURAGE, 2007	85	1149	95	1138	0.89 [0.67, 1.17]
JSAP, 2008	6	188	7	191	0.87 [0.30, 2.54]
BARI 2D, 2009	102	798	96	807	1.07 [0.83, 1.39]
FAME 2, 2012	1	447	3	441	0.33 [0.03, 3.15]
**Stable CAD studies (p = 0.98, Q = 14.69, df = 13.00, p for heterogeneity = 0.33; I^2 = 0.0%)**					1.00 [0.85, 1.18]

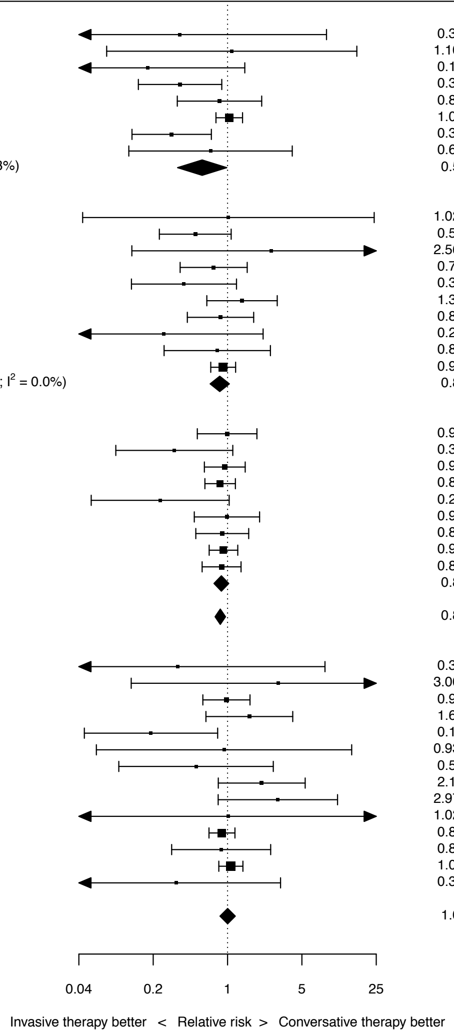

Online Figure 36. Sensitivity analysis for primary outcome of all-cause mortality excluding the Hambrecht trial

Study and Year	Active Events	N	Control Events	N	Relative risk [95% CI]
Unstable CAD – Unrevascularized post–MI					
TOPS, 1992	0	42	1	45	0.36 [0.01, 8.52]
DAKIK, 1998	1	21	1	23	1.10 [0.07, 16.43]
Horie, 1998	1	44	5	39	0.18 [0.02, 1.45]
ALKK, 2003	6	149	17	151	0.36 [0.15, 0.88]
DECOPI, 2004	8	109	9	103	0.84 [0.34, 2.09]
OAT, 2006	87	1082	84	1084	1.04 [0.78, 1.38]
SWISSI 2, 2007	6	96	22	105	0.30 [0.13, 0.70]
VIAMI, 2012	2	106	3	110	0.69 [0.12, 4.06]
Unrevascularized post–MI studies (**p = 0.04**, Q = 13.52, df = 7, p for heterogeneity = 0.06; I^2 = 48.3%)					0.58 [0.34, 0.98]
Unstable CAD – Multivessel disease following STEMI					
Help–AMI, 2009	1	52	0	17	1.02 [0.04, 23.91]
Politi, 2010	10	130	13	84	0.50 [0.23, 1.08]
Dambrink, 2012	2	79	0	40	2.56 [0.13, 52.14]
PRAMI, 2013	12	234	16	231	0.74 [0.36, 1.53]
CvLPRIT, 2015	4	150	10	146	0.39 [0.12, 1.21]
DANAMI 3, 2015	15	313	11	314	1.37 [0.64, 2.93]
Zhang, 2015	13	215	15	213	0.86 [0.42, 1.76]
Hamza, 2016	1	50	4	50	0.25 [0.03, 2.16]
Compare ACUTE, 2017	4	295	10	590	0.80 [0.25, 2.53]
Complete, 2019	96	2016	106	2025	0.91 [0.70, 1.19]
Multivessel disease following STEMI studies (**p = 0.11**, Q = 7.30, df = 9, p for heterogeneity = 0.61; I^2 = 0.0%)					0.84 [0.69, 1.04]
Unstable CAD – NSTEMI					
TIMI IIIB, 1994	18	740	18	733	0.99 [0.52, 1.89]
FRISC II, 2000	27	1222	48	1234	0.57 [0.36, 0.90]
TRUCS, 2000	3	76	9	72	0.32 [0.09, 1.12]
TACTICS 18, 2001	37	1114	39	1106	0.94 [0.61, 1.47]
RITA 3, 2002	60	895	72	915	0.85 [0.61, 1.19]
VINO, 2002	2	64	9	67	0.23 [0.05, 1.04]
ICTUS, 2005	15	604	15	596	0.99 [0.49, 2.00]
Savonitto, 2012	19	154	22	159	0.89 [0.50, 1.58]
After 80, 2016	57	229	62	228	0.92 [0.67, 1.25]
Sanchis, 2016	22	52	26	54	0.88 [0.58, 1.34]
NSTEMI studies (**p = 0.02**, Q = 8.95, df = 9.00, p for heterogeneity = 0.44; I^2 = 0.0%)					0.84 [0.72, 0.97]
**Unstable CAD studies (p = 0.001, Q = 29.79, df = 27.00, p for heterogeneity = 0.32; I^2 = 0.0%)**					0.84 [0.75, 0.93]
Stable CAD					
ACME 1, 1992	0	105	1	107	0.34 [0.01, 8.24]
MASS 1, 1995	1	72	0	72	3.00 [0.12, 72.44]
ACME 2, 1997	25	166	25	162	0.98 [0.59, 1.63]
RITA 2, 1997	11	504	7	514	1.60 [0.63, 4.10]
ACIP, 1997	2	192	20	366	0.19 [0.05, 0.81]
AVERT, 1999	1	177	1	164	0.93 [0.06, 14.69]
DEFER, 2001	2	90	4	91	0.51 [0.09, 2.69]
TIME, 2001	13	153	6	148	2.10 [0.82, 5.37]
MASS 2, 2004	9	205	3	203	2.97 [0.82, 10.82]
COURAGE, 2007	85	1149	95	1138	0.89 [0.67, 1.17]
JSAP, 2008	6	188	7	191	0.87 [0.30, 2.54]
BARI 2D, 2009	102	798	96	807	1.07 [0.83, 1.39]
FAME 2, 2012	1	447	3	441	0.33 [0.03, 3.15]
**Stable CAD studies (p = 0.98, Q = 14.69, df = 12.00, p for heterogeneity = 0.26; I^2 = 0.0%)**					1.00 [0.85, 1.18]

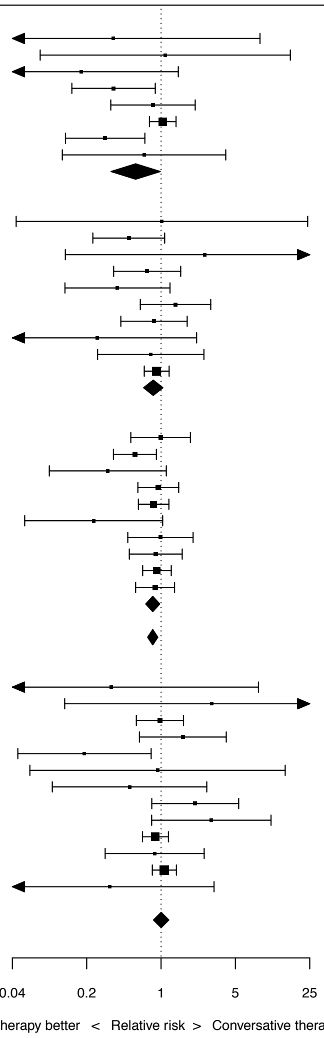

Online Figure 37. Sensitivity analysis for primary outcome of all-cause mortality excluding the Hamza trial

Study and Year	Active Events	N	Control Events	N	Relative risk [95% CI]
Unstable CAD – Unrevascularized post–MI					
TOPS, 1992	0	42	1	45	0.36 [0.01, 8.52]
DAKIK, 1998	1	21	1	23	1.10 [0.07, 16.43]
Horie, 1998	1	44	5	39	0.18 [0.02, 1.45]
ALKK, 2003	6	149	17	151	0.36 [0.15, 0.88]
DECOPI, 2004	8	109	9	103	0.84 [0.34, 2.09]
OAT, 2006	87	1082	84	1084	1.04 [0.78, 1.38]
SWISSI 2, 2007	6	96	22	105	0.30 [0.13, 0.70]
VIAMI, 2012	2	106	3	110	0.69 [0.12, 4.06]
Unrevascularized post–MI studies (**p = 0.04**, Q = 13.52, df = 7, p for heterogeneity = 0.06; I^2 = 48.3%)					0.58 [0.34, 0.98]
Unstable CAD – Multivessel disease following STEMI					
Help–AMI, 2009	1	52	0	17	1.02 [0.04, 23.91]
Politi, 2010	10	130	13	84	0.50 [0.23, 1.08]
Dambrink, 2012	2	79	0	40	2.56 [0.13, 52.14]
PRAMI, 2013	12	234	16	231	0.74 [0.36, 1.53]
CvLPRIT, 2015	4	150	10	146	0.39 [0.12, 1.21]
DANAMI 3, 2015	15	313	11	314	1.37 [0.64, 2.93]
Zhang, 2015	13	215	15	213	0.86 [0.42, 1.76]
Compare ACUTE, 2017	4	295	10	590	0.80 [0.25, 2.53]
Complete, 2019	96	2016	106	2025	0.91 [0.70, 1.19]
Multivessel disease following STEMI studies (**p = 0.14**, Q = 6.06, df = 8, p for heterogeneity = 0.64; I^2 = 0.0%)					0.85 [0.69, 1.05]
Unstable CAD – NSTEMI					
TIMI IIIB, 1994	18	740	18	733	0.99 [0.52, 1.89]
FRISC II, 2000	27	1222	48	1234	0.57 [0.36, 0.90]
TRUCS, 2000	3	76	9	72	0.32 [0.09, 1.12]
TACTICS 18, 2001	37	1114	39	1106	0.94 [0.61, 1.47]
RITA 3, 2002	60	895	72	915	0.85 [0.61, 1.19]
VINO, 2002	2	64	9	67	0.23 [0.05, 1.04]
ICTUS, 2005	15	604	15	596	0.99 [0.49, 2.00]
Savonitto, 2012	19	154	22	159	0.89 [0.50, 1.58]
After 80, 2016	57	229	62	228	0.92 [0.67, 1.25]
Sanchis, 2016	22	52	26	54	0.88 [0.58, 1.34]
NSTEMI studies (**p = 0.02**, Q = 8.95, df = 9.00, p for heterogeneity = 0.44; I^2 = 0.0%)					0.84 [0.72, 0.97]
**Unstable CAD studies (p = 0.002, Q = 28.58, df = 26.00, p for heterogeneity = 0.33; I^2 = 0.0%)**					0.84 [0.75, 0.94]
Stable CAD					
ACME 1, 1992	0	105	1	107	0.34 [0.01, 8.24]
MASS 1, 1995	1	72	0	72	3.00 [0.12, 72.44]
ACME 2, 1997	25	166	25	162	0.98 [0.59, 1.63]
RITA 2, 1997	11	504	7	514	1.60 [0.63, 4.10]
ACIP, 1997	2	192	20	366	0.19 [0.05, 0.81]
AVERT, 1999	1	177	1	164	0.93 [0.06, 14.69]
DEFER, 2001	2	90	4	91	0.51 [0.09, 2.69]
TIME, 2001	13	153	6	148	2.10 [0.82, 5.37]
MASS 2, 2004	9	205	3	203	2.97 [0.82, 10.82]
Hambrecht, 2004	0	50	0	51	1.02 [0.02, 50.41]
COURAGE, 2007	85	1149	95	1138	0.89 [0.67, 1.17]
JSAP, 2008	6	188	7	191	0.87 [0.30, 2.54]
BARI 2D, 2009	102	798	96	807	1.07 [0.83, 1.39]
FAME 2, 2012	1	447	3	441	0.33 [0.03, 3.15]
**Stable CAD studies (p = 0.98, Q = 14.69, df = 13.00, p for heterogeneity = 0.33; I^2 = 0.0%)**					1.00 [0.85, 1.18]

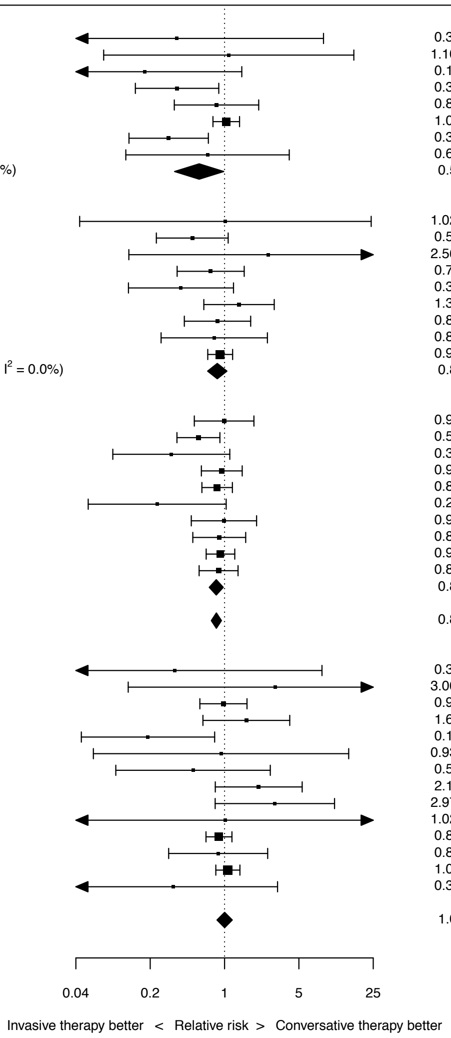

Online Figure 38. Sensitivity analysis for primary outcome of all-cause mortality excluding the HELP-AMI trial

Study and Year	Active Events	Active N	Control Events	Control N	Relative risk [95% CI]
Unstable CAD – Unrevascularized post–MI					
TOPS, 1992	0	42	1	45	0.36 [0.01, 8.52]
DAKIK, 1998	1	21	1	23	1.10 [0.07, 16.43]
Horie, 1998	1	44	5	39	0.18 [0.02, 1.45]
ALKK, 2003	6	149	17	151	0.36 [0.15, 0.88]
DECOPI, 2004	8	109	9	103	0.84 [0.34, 2.09]
OAT, 2006	87	1082	84	1084	1.04 [0.78, 1.38]
SWISSI 2, 2007	6	96	22	105	0.30 [0.13, 0.70]
VIAMI, 2012	2	106	3	110	0.69 [0.12, 4.06]
Unrevascularized post–MI studies (**p = 0.04**, Q = 13.52, df = 7, p for heterogeneity = 0.06; I^2 = 48.3%)					0.58 [0.34, 0.98]
Unstable CAD – Multivessel disease following STEMI					
Politi, 2010	10	130	13	84	0.50 [0.23, 1.08]
Dambrink, 2012	2	79	0	40	2.56 [0.13, 52.14]
PRAMI, 2013	12	234	16	231	0.74 [0.36, 1.53]
CvLPRIT, 2015	4	150	10	146	0.39 [0.12, 1.21]
DANAMI 3, 2015	15	313	11	314	1.37 [0.64, 2.93]
Zhang, 2015	13	215	15	213	0.86 [0.42, 1.76]
Hamza, 2016	1	50	4	50	0.25 [0.03, 2.16]
Compare ACUTE, 2017	4	295	10	590	0.80 [0.25, 2.53]
Complete, 2019	96	2016	106	2025	0.91 [0.70, 1.19]
Multivessel disease following STEMI studies (**p = 0.11**, Q = 7.28, df = 8, p for heterogeneity = 0.51; I^2 = 0.0%)					0.84 [0.68, 1.04]
Unstable CAD – NSTEMI					
TIMI IIIB, 1994	18	740	18	733	0.99 [0.52, 1.89]
FRISC II, 2000	27	1222	48	1234	0.57 [0.36, 0.90]
TRUCS, 2000	3	76	9	72	0.32 [0.09, 1.12]
TACTICS 18, 2001	37	1114	39	1106	0.94 [0.61, 1.47]
RITA 3, 2002	60	895	72	915	0.85 [0.61, 1.19]
VINO, 2002	2	64	9	67	0.23 [0.05, 1.04]
ICTUS, 2005	15	604	15	596	0.99 [0.49, 2.00]
Savonitto, 2012	19	154	22	159	0.89 [0.50, 1.58]
After 80, 2016	57	229	62	228	0.92 [0.67, 1.25]
Sanchis, 2016	22	52	26	54	0.88 [0.58, 1.34]
NSTEMI studies (**p = 0.02**, Q = 8.95, df = 9.00, p for heterogeneity = 0.44; I^2 = 0.0%)					0.84 [0.72, 0.97]
**Unstable CAD studies (p = 0.001, Q = 29.77, df = 26.00, p for heterogeneity = 0.28; I^2 = 0.0%)**					0.84 [0.75, 0.93]
Stable CAD					
ACME 1, 1992	0	105	1	107	0.34 [0.01, 8.24]
MASS 1, 1995	1	72	0	72	3.00 [0.12, 72.44]
ACME 2, 1997	25	166	25	162	0.98 [0.59, 1.63]
RITA 2, 1997	11	504	7	514	1.60 [0.63, 4.10]
ACIP, 1997	2	192	20	366	0.19 [0.05, 0.81]
AVERT, 1999	1	177	1	164	0.93 [0.06, 14.69]
DEFER, 2001	2	90	4	91	0.51 [0.09, 2.69]
TIME, 2001	13	153	6	148	2.10 [0.82, 5.37]
MASS 2, 2004	9	205	3	203	2.97 [0.82, 10.82]
Hambrecht, 2004	0	50	0	51	1.02 [0.02, 50.41]
COURAGE, 2007	85	1149	95	1138	0.89 [0.67, 1.17]
JSAP, 2008	6	188	7	191	0.87 [0.30, 2.54]
BARI 2D, 2009	102	798	96	807	1.07 [0.83, 1.39]
FAME 2, 2012	1	447	3	441	0.33 [0.03, 3.15]
**Stable CAD studies (p = 0.98, Q = 14.69, df = 13.00, p for heterogeneity = 0.33; I^2 = 0.0%)**					1.00 [0.85, 1.18]

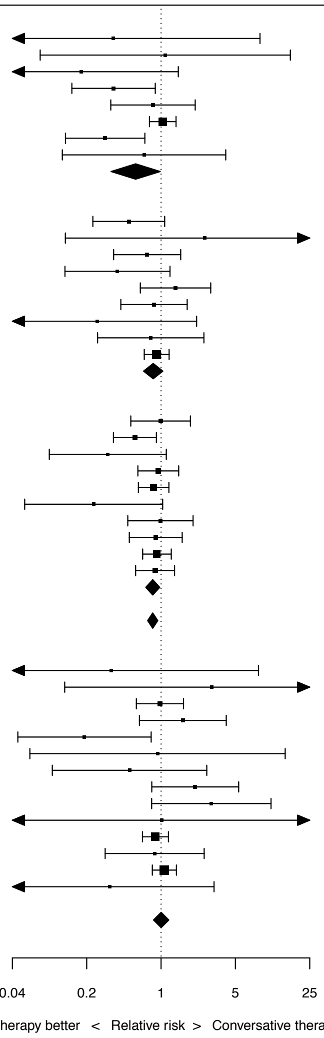

Online Figure 39. Sensitivity analysis for primary outcome of all-cause mortality excluding the Horie trial

Study and Year	Active Events	Active N	Control Events	Control N	Relative risk [95% CI]
Unstable CAD – Unrevascularized post–MI					
TOPS, 1992	0	42	1	45	0.36 [0.01, 8.52]
DAKIK, 1998	1	21	1	23	1.10 [0.07, 16.43]
ALKK, 2003	6	149	17	151	0.36 [0.15, 0.88]
DECOPI, 2004	8	109	9	103	0.84 [0.34, 2.09]
OAT, 2006	87	1082	84	1084	1.04 [0.78, 1.38]
SWISSI 2, 2007	6	96	22	105	0.30 [0.13, 0.70]
VIAMI, 2012	2	106	3	110	0.69 [0.12, 4.06]
Unrevascularized post–MI studies (**p = 0.08**, Q = 11.45, df = 6, p for heterogeneity = 0.08; I^2 = 49.5%)					0.62 [0.36, 1.06]
Unstable CAD – Multivessel disease following STEMI					
Help–AMI, 2009	1	52	0	17	1.02 [0.04, 23.91]
Politi, 2010	10	130	13	84	0.50 [0.23, 1.08]
Dambrink, 2012	2	79	0	40	2.56 [0.13, 52.14]
PRAMI, 2013	12	234	16	231	0.74 [0.36, 1.53]
CvLPRIT, 2015	4	150	10	146	0.39 [0.12, 1.21]
DANAMI 3, 2015	15	313	11	314	1.37 [0.64, 2.93]
Zhang, 2015	13	215	15	213	0.86 [0.42, 1.76]
Hamza, 2016	1	50	4	50	0.25 [0.03, 2.16]
Compare ACUTE, 2017	4	295	10	590	0.80 [0.25, 2.53]
Complete, 2019	96	2016	106	2025	0.91 [0.70, 1.19]
Multivessel disease following STEMI studies (**p = 0.11**, Q = 7.30, df = 9, p for heterogeneity = 0.61; I^2 = 0.0%)					0.84 [0.69, 1.04]
Unstable CAD – NSTEMI					
TIMI IIIB, 1994	18	740	18	733	0.99 [0.52, 1.89]
FRISC II, 2000	27	1222	48	1234	0.57 [0.36, 0.90]
TRUCS, 2000	3	76	9	72	0.32 [0.09, 1.12]
TACTICS 18, 2001	37	1114	39	1106	0.94 [0.61, 1.47]
RITA 3, 2002	60	895	72	915	0.85 [0.61, 1.19]
VINO, 2002	2	64	9	67	0.23 [0.05, 1.04]
ICTUS, 2005	15	604	15	596	0.99 [0.49, 2.00]
Savonitto, 2012	19	154	22	159	0.89 [0.50, 1.58]
After 80, 2016	57	229	62	228	0.92 [0.67, 1.25]
Sanchis, 2016	22	52	26	54	0.88 [0.58, 1.34]
NSTEMI studies (**p = 0.02**, Q = 8.95, df = 9.00, p for heterogeneity = 0.44; I^2 = 0.0%)					0.84 [0.72, 0.97]
**Unstable CAD studies (p = 0.002, Q = 27.70, df = 26.00, p for heterogeneity = 0.37; I^2 = 0.0%)**					0.84 [0.75, 0.94]
Stable CAD					
ACME 1, 1992	0	105	1	107	0.34 [0.01, 8.24]
MASS 1, 1995	1	72	0	72	3.00 [0.12, 72.44]
ACME 2, 1997	25	166	25	162	0.98 [0.59, 1.63]
RITA 2, 1997	11	504	7	514	1.60 [0.63, 4.10]
ACIP, 1997	2	192	20	366	0.19 [0.05, 0.81]
AVERT, 1999	1	177	1	164	0.93 [0.06, 14.69]
DEFER, 2001	2	90	4	91	0.51 [0.09, 2.69]
TIME, 2001	13	153	6	148	2.10 [0.82, 5.37]
MASS 2, 2004	9	205	3	203	2.97 [0.82, 10.82]
Hambrecht, 2004	0	50	0	51	1.02 [0.02, 50.41]
COURAGE, 2007	85	1149	95	1138	0.89 [0.67, 1.17]
JSAP, 2008	6	188	7	191	0.87 [0.30, 2.54]
BARI 2D, 2009	102	798	96	807	1.07 [0.83, 1.39]
FAME 2, 2012	1	447	3	441	0.33 [0.03, 3.15]
**Stable CAD studies (p = 0.98, Q = 14.69, df = 13.00, p for heterogeneity = 0.33; I^2 = 0.0%)**					1.00 [0.85, 1.18]

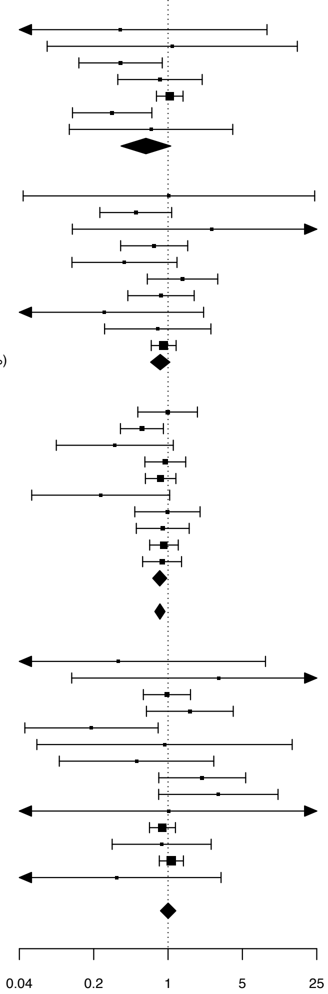

Invasive therapy better < Relative risk > Conversative therapy better

Online Figure 40. Sensitivity analysis for primary outcome of all-cause mortality excluding the ICTUS trial

Study and Year	Active Events	N	Control Events	N	Relative risk [95% CI]
Unstable CAD – Unrevascularized post–MI					
TOPS, 1992	0	42	1	45	0.36 [0.01, 8.52]
DAKIK, 1998	1	21	1	23	1.10 [0.07, 16.43]
Horie, 1998	1	44	5	39	0.18 [0.02, 1.45]
ALKK, 2003	6	149	17	151	0.36 [0.15, 0.88]
DECOPI, 2004	8	109	9	103	0.84 [0.34, 2.09]
OAT, 2006	87	1082	84	1084	1.04 [0.78, 1.38]
SWISSI 2, 2007	6	96	22	105	0.30 [0.13, 0.70]
VIAMI, 2012	2	106	3	110	0.69 [0.12, 4.06]
Unrevascularized post–MI studies (**p = 0.04**, Q = 13.52, df = 7, p for heterogeneity = 0.06; I^2 = 48.3%)					0.58 [0.34, 0.98]
Unstable CAD – Multivessel disease following STEMI					
Help–AMI, 2009	1	52	0	17	1.02 [0.04, 23.91]
Politi, 2010	10	130	13	84	0.50 [0.23, 1.08]
Dambrink, 2012	2	79	0	40	2.56 [0.13, 52.14]
PRAMI, 2013	12	234	16	231	0.74 [0.36, 1.53]
CvLPRIT, 2015	4	150	10	146	0.39 [0.12, 1.21]
DANAMI 3, 2015	15	313	11	314	1.37 [0.64, 2.93]
Zhang, 2015	13	215	15	213	0.86 [0.42, 1.76]
Hamza, 2016	1	50	4	50	0.25 [0.03, 2.16]
Compare ACUTE, 2017	4	295	10	590	0.80 [0.25, 2.53]
Complete, 2019	96	2016	106	2025	0.91 [0.70, 1.19]
Multivessel disease following STEMI studies (**p = 0.11**, Q = 7.30, df = 9, p for heterogeneity = 0.61; I^2 = 0.0%)					0.84 [0.69, 1.04]
Unstable CAD – NSTEMI					
TIMI IIIB, 1994	18	740	18	733	0.99 [0.52, 1.89]
FRISC II, 2000	27	1222	48	1234	0.57 [0.36, 0.90]
TRUCS, 2000	3	76	9	72	0.32 [0.09, 1.12]
TACTICS 18, 2001	37	1114	39	1106	0.94 [0.61, 1.47]
RITA 3, 2002	60	895	72	915	0.85 [0.61, 1.19]
VINO, 2002	2	64	9	67	0.23 [0.05, 1.04]
Savonitto, 2012	19	154	22	159	0.89 [0.50, 1.58]
After 80, 2016	57	229	62	228	0.92 [0.67, 1.25]
Sanchis, 2016	22	52	26	54	0.88 [0.58, 1.34]
NSTEMI studies (**p = 0.02**, Q = 8.72, df = 8.00, p for heterogeneity = 0.37; I^2 = 0.0%)					0.83 [0.71, 0.97]
**Unstable CAD studies (p = 0.001, Q = 29.57, df = 26.00, p for heterogeneity = 0.29; I^2 = 1.0%)**					0.83 [0.74, 0.93]
Stable CAD					
ACME 1, 1992	0	105	1	107	0.34 [0.01, 8.24]
MASS 1, 1995	1	72	0	72	3.00 [0.12, 72.44]
ACME 2, 1997	25	166	25	162	0.98 [0.59, 1.63]
RITA 2, 1997	11	504	7	514	1.60 [0.63, 4.10]
ACIP, 1997	2	192	20	366	0.19 [0.05, 0.81]
AVERT, 1999	1	177	1	164	0.93 [0.06, 14.69]
DEFER, 2001	2	90	4	91	0.51 [0.09, 2.69]
TIME, 2001	13	153	6	148	2.10 [0.82, 5.37]
MASS 2, 2004	9	205	3	203	2.97 [0.82, 10.82]
Hambrecht, 2004	0	50	0	51	1.02 [0.02, 50.41]
COURAGE, 2007	85	1149	95	1138	0.89 [0.67, 1.17]
JSAP, 2008	6	188	7	191	0.87 [0.30, 2.54]
BARI 2D, 2009	102	798	96	807	1.07 [0.83, 1.39]
FAME 2, 2012	1	447	3	441	0.33 [0.03, 3.15]
**Stable CAD studies (p = 0.98, Q = 14.69, df = 13.00, p for heterogeneity = 0.33; I^2 = 0.0%)**					1.00 [0.85, 1.18]

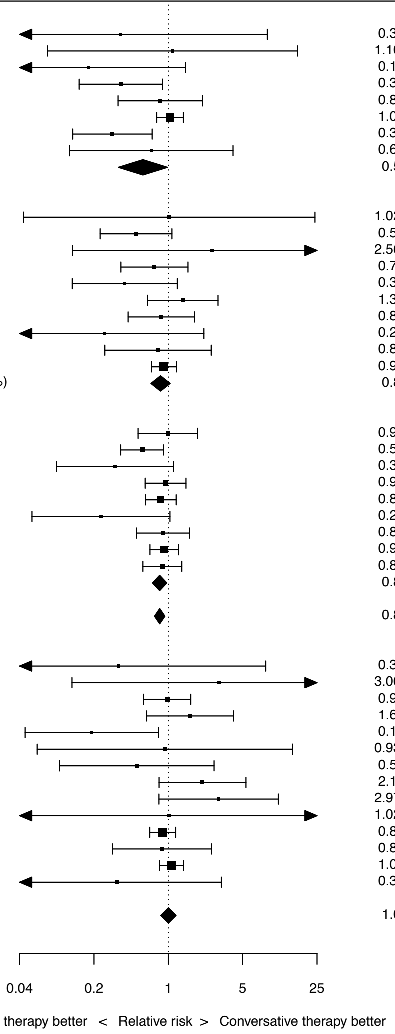

Online Figure 41. Sensitivity analysis for primary outcome of all-cause mortality excluding the JSAP trial

Study and Year	Active Events	N	Control Events	N	Relative risk [95% CI]
Unstable CAD – Unrevascularized post–MI					
TOPS, 1992	0	42	1	45	0.36 [0.01, 8.52]
DAKIK, 1998	1	21	1	23	1.10 [0.07, 16.43]
Horie, 1998	1	44	5	39	0.18 [0.02, 1.45]
ALKK, 2003	6	149	17	151	0.36 [0.15, 0.88]
DECOPI, 2004	8	109	9	103	0.84 [0.34, 2.09]
OAT, 2006	87	1082	84	1084	1.04 [0.78, 1.38]
SWISSI 2, 2007	6	96	22	105	0.30 [0.13, 0.70]
VIAMI, 2012	2	106	3	110	0.69 [0.12, 4.06]
Unrevascularized post–MI studies (**p = 0.04**, Q = 13.52, df = 7, p for heterogeneity = 0.06; I^2 = 48.3%)					0.58 [0.34, 0.98]
Unstable CAD – Multivessel disease following STEMI					
Help–AMI, 2009	1	52	0	17	1.02 [0.04, 23.91]
Politi, 2010	10	130	13	84	0.50 [0.23, 1.08]
Dambrink, 2012	2	79	0	40	2.56 [0.13, 52.14]
PRAMI, 2013	12	234	16	231	0.74 [0.36, 1.53]
CvLPRIT, 2015	4	150	10	146	0.39 [0.12, 1.21]
DANAMI 3, 2015	15	313	11	314	1.37 [0.64, 2.93]
Zhang, 2015	13	215	15	213	0.86 [0.42, 1.76]
Hamza, 2016	1	50	4	50	0.25 [0.03, 2.16]
Compare ACUTE, 2017	4	295	10	590	0.80 [0.25, 2.53]
Complete, 2019	96	2016	106	2025	0.91 [0.70, 1.19]
Multivessel disease following STEMI studies (**p = 0.11**, Q = 7.30, df = 9, p for heterogeneity = 0.61; I^2 = 0.0%)					0.84 [0.69, 1.04]
Unstable CAD – NSTEMI					
TIMI IIIB, 1994	18	740	18	733	0.99 [0.52, 1.89]
FRISC II, 2000	27	1222	48	1234	0.57 [0.36, 0.90]
TRUCS, 2000	3	76	9	72	0.32 [0.09, 1.12]
TACTICS 18, 2001	37	1114	39	1106	0.94 [0.61, 1.47]
RITA 3, 2002	60	895	72	915	0.85 [0.61, 1.19]
VINO, 2002	2	64	9	67	0.23 [0.05, 1.04]
ICTUS, 2005	15	604	15	596	0.99 [0.49, 2.00]
Savonitto, 2012	19	154	22	159	0.89 [0.50, 1.58]
After 80, 2016	57	229	62	228	0.92 [0.67, 1.25]
Sanchis, 2016	22	52	26	54	0.88 [0.58, 1.34]
NSTEMI studies (**p = 0.02**, Q = 8.95, df = 9.00, p for heterogeneity = 0.44; I^2 = 0.0%)					0.84 [0.72, 0.97]
**Unstable CAD studies (p = 0.001, Q = 29.79, df = 27.00, p for heterogeneity = 0.32; I^2 = 0.0%)**					0.84 [0.75, 0.93]
Stable CAD					
ACME 1, 1992	0	105	1	107	0.34 [0.01, 8.24]
MASS 1, 1995	1	72	0	72	3.00 [0.12, 72.44]
ACME 2, 1997	25	166	25	162	0.98 [0.59, 1.63]
RITA 2, 1997	11	504	7	514	1.60 [0.63, 4.10]
ACIP, 1997	2	192	20	366	0.19 [0.05, 0.81]
AVERT, 1999	1	177	1	164	0.93 [0.06, 14.69]
DEFER, 2001	2	90	4	91	0.51 [0.09, 2.69]
TIME, 2001	13	153	6	148	2.10 [0.82, 5.37]
MASS 2, 2004	9	205	3	203	2.97 [0.82, 10.82]
Hambrecht, 2004	0	50	0	51	1.02 [0.02, 50.41]
COURAGE, 2007	85	1149	95	1138	0.89 [0.67, 1.17]
BARI 2D, 2009	102	798	96	807	1.07 [0.83, 1.39]
FAME 2, 2012	1	447	3	441	0.33 [0.03, 3.15]
**Stable CAD studies (p = 0.95, Q = 14.62, df = 12.00, p for heterogeneity = 0.26; I^2 = 0.0%)**					1.01 [0.85, 1.19]

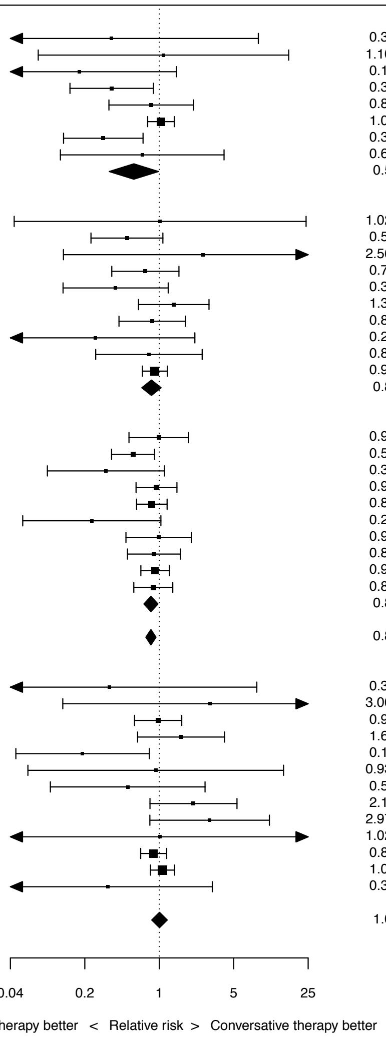

Online Figure 42. Sensitivity analysis for primary outcome of all-cause mortality excluding the MASS 1 trial

Study and Year	Active Events	Active N	Control Events	Control N	Relative risk [95% CI]
Unstable CAD – Unrevascularized post–MI					
TOPS, 1992	0	42	1	45	0.36 [0.01, 8.52]
DAKIK, 1998	1	21	1	23	1.10 [0.07, 16.43]
Horie, 1998	1	44	5	39	0.18 [0.02, 1.45]
ALKK, 2003	6	149	17	151	0.36 [0.15, 0.88]
DECOPI, 2004	8	109	9	103	0.84 [0.34, 2.09]
OAT, 2006	87	1082	84	1084	1.04 [0.78, 1.38]
SWISSI 2, 2007	6	96	22	105	0.30 [0.13, 0.70]
VIAMI, 2012	2	106	3	110	0.69 [0.12, 4.06]
Unrevascularized post–MI studies (**p = 0.04**, Q = 13.52, df = 7, p for heterogeneity = 0.06; I^2 = 48.3%)					0.58 [0.34, 0.98]
Unstable CAD – Multivessel disease following STEMI					
Help–AMI, 2009	1	52	0	17	1.02 [0.04, 23.91]
Politi, 2010	10	130	13	84	0.50 [0.23, 1.08]
Dambrink, 2012	2	79	0	40	2.56 [0.13, 52.14]
PRAMI, 2013	12	234	16	231	0.74 [0.36, 1.53]
CvLPRIT, 2015	4	150	10	146	0.39 [0.12, 1.21]
DANAMI 3, 2015	15	313	11	314	1.37 [0.64, 2.93]
Zhang, 2015	13	215	15	213	0.86 [0.42, 1.76]
Hamza, 2016	1	50	4	50	0.25 [0.03, 2.16]
Compare ACUTE, 2017	4	295	10	590	0.80 [0.25, 2.53]
Complete, 2019	96	2016	106	2025	0.91 [0.70, 1.19]
Multivessel disease following STEMI studies (**p = 0.11**, Q = 7.30, df = 9, p for heterogeneity = 0.61; I^2 = 0.0%)					0.84 [0.69, 1.04]
Unstable CAD – NSTEMI					
TIMI IIIB, 1994	18	740	18	733	0.99 [0.52, 1.89]
FRISC II, 2000	27	1222	48	1234	0.57 [0.36, 0.90]
TRUCS, 2000	3	76	9	72	0.32 [0.09, 1.12]
TACTICS 18, 2001	37	1114	39	1106	0.94 [0.61, 1.47]
RITA 3, 2002	60	895	72	915	0.85 [0.61, 1.19]
VINO, 2002	2	64	9	67	0.23 [0.05, 1.04]
ICTUS, 2005	15	604	15	596	0.99 [0.49, 2.00]
Savonitto, 2012	19	154	22	159	0.89 [0.50, 1.58]
After 80, 2016	57	229	62	228	0.92 [0.67, 1.25]
Sanchis, 2016	22	52	26	54	0.88 [0.58, 1.34]
NSTEMI studies (**p = 0.02**, Q = 8.95, df = 9.00, p for heterogeneity = 0.44; I^2 = 0.0%)					0.84 [0.72, 0.97]
**Unstable CAD studies (p = 0.001, Q = 29.79, df = 27.00, p for heterogeneity = 0.32; I^2 = 0.0%)**					0.84 [0.75, 0.93]
Stable CAD					
ACME 1, 1992	0	105	1	107	0.34 [0.01, 8.24]
ACME 2, 1997	25	166	25	162	0.98 [0.59, 1.63]
RITA 2, 1997	11	504	7	514	1.60 [0.63, 4.10]
ACIP, 1997	2	192	20	366	0.19 [0.05, 0.81]
AVERT, 1999	1	177	1	164	0.93 [0.06, 14.69]
DEFER, 2001	2	90	4	91	0.51 [0.09, 2.69]
TIME, 2001	13	153	6	148	2.10 [0.82, 5.37]
MASS 2, 2004	9	205	3	203	2.97 [0.82, 10.82]
Hambrecht, 2004	0	50	0	51	1.02 [0.02, 50.41]
COURAGE, 2007	85	1149	95	1138	0.89 [0.67, 1.17]
JSAP, 2008	6	188	7	191	0.87 [0.30, 2.54]
BARI 2D, 2009	102	798	96	807	1.07 [0.83, 1.39]
FAME 2, 2012	1	447	3	441	0.33 [0.03, 3.15]
**Stable CAD studies (p = 0.99, Q = 14.23, df = 12.00, p for heterogeneity = 0.29; I^2 = 0.0%)**					1.00 [0.85, 1.18]

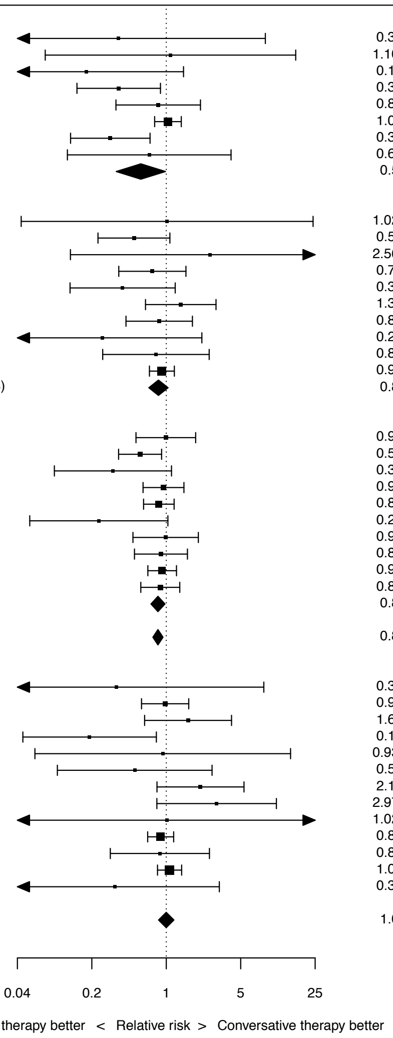

Online Figure 43. Sensitivity analysis for primary outcome of all-cause mortality excluding the MASS 2 trial

Study and Year	Active Events	N	Control Events	N	Relative risk [95% CI]
Unstable CAD – Unrevascularized post–MI					
TOPS, 1992	0	42	1	45	0.36 [0.01, 8.52]
DAKIK, 1998	1	21	1	23	1.10 [0.07, 16.43]
Horie, 1998	1	44	5	39	0.18 [0.02, 1.45]
ALKK, 2003	6	149	17	151	0.36 [0.15, 0.88]
DECOPI, 2004	8	109	9	103	0.84 [0.34, 2.09]
OAT, 2006	87	1082	84	1084	1.04 [0.78, 1.38]
SWISSI 2, 2007	6	96	22	105	0.30 [0.13, 0.70]
VIAMI, 2012	2	106	3	110	0.69 [0.12, 4.06]
Unrevascularized post–MI studies (**p = 0.04**, Q = 13.52, df = 7, p for heterogeneity = 0.06; I^2 = 48.3%)					0.58 [0.34, 0.98]
Unstable CAD – Multivessel disease following STEMI					
Help–AMI, 2009	1	52	0	17	1.02 [0.04, 23.91]
Politi, 2010	10	130	13	84	0.50 [0.23, 1.08]
Dambrink, 2012	2	79	0	40	2.56 [0.13, 52.14]
PRAMI, 2013	12	234	16	231	0.74 [0.36, 1.53]
CvLPRIT, 2015	4	150	10	146	0.39 [0.12, 1.21]
DANAMI 3, 2015	15	313	11	314	1.37 [0.64, 2.93]
Zhang, 2015	13	215	15	213	0.86 [0.42, 1.76]
Hamza, 2016	1	50	4	50	0.25 [0.03, 2.16]
Compare ACUTE, 2017	4	295	10	590	0.80 [0.25, 2.53]
Complete, 2019	96	2016	106	2025	0.91 [0.70, 1.19]
Multivessel disease following STEMI studies (**p = 0.11**, Q = 7.30, df = 9, p for heterogeneity = 0.61; I^2 = 0.0%)					0.84 [0.69, 1.04]
Unstable CAD – NSTEMI					
TIMI IIIB, 1994	18	740	18	733	0.99 [0.52, 1.89]
FRISC II, 2000	27	1222	48	1234	0.57 [0.36, 0.90]
TRUCS, 2000	3	76	9	72	0.32 [0.09, 1.12]
TACTICS 18, 2001	37	1114	39	1106	0.94 [0.61, 1.47]
RITA 3, 2002	60	895	72	915	0.85 [0.61, 1.19]
VINO, 2002	2	64	9	67	0.23 [0.05, 1.04]
ICTUS, 2005	15	604	15	596	0.99 [0.49, 2.00]
Savonitto, 2012	19	154	22	159	0.89 [0.50, 1.58]
After 80, 2016	57	229	62	228	0.92 [0.67, 1.25]
Sanchis, 2016	22	52	26	54	0.88 [0.58, 1.34]
NSTEMI studies (**p = 0.02**, Q = 8.95, df = 9.00, p for heterogeneity = 0.44; I^2 = 0.0%)					0.84 [0.72, 0.97]
**Unstable CAD studies (p = 0.001, Q = 29.79, df = 27.00, p for heterogeneity = 0.32; I^2 = 0.0%)**					0.84 [0.75, 0.93]
Stable CAD					
ACME 1, 1992	0	105	1	107	0.34 [0.01, 8.24]
MASS 1, 1995	1	72	0	72	3.00 [0.12, 72.44]
ACME 2, 1997	25	166	25	162	0.98 [0.59, 1.63]
RITA 2, 1997	11	504	7	514	1.60 [0.63, 4.10]
ACIP, 1997	2	192	20	366	0.19 [0.05, 0.81]
AVERT, 1999	1	177	1	164	0.93 [0.06, 14.69]
DEFER, 2001	2	90	4	91	0.51 [0.09, 2.69]
TIME, 2001	13	153	6	148	2.10 [0.82, 5.37]
Hambrecht, 2004	0	50	0	51	1.02 [0.02, 50.41]
COURAGE, 2007	85	1149	95	1138	0.89 [0.67, 1.17]
JSAP, 2008	6	188	7	191	0.87 [0.30, 2.54]
BARI 2D, 2009	102	798	96	807	1.07 [0.83, 1.39]
FAME 2, 2012	1	447	3	441	0.33 [0.03, 3.15]
**Stable CAD studies (p = 0.85, Q = 11.92, df = 12.00, p for heterogeneity = 0.45; I^2 = 0.0%)**					0.98 [0.83, 1.16]

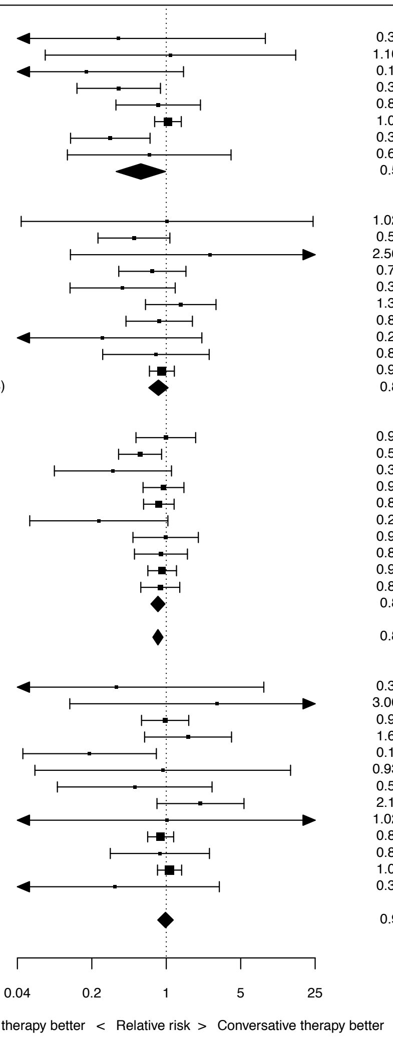

Online Figure 44. Sensitivity analysis for primary outcome of all-cause mortality excluding the OAT trial

Study and Year	Active Events	Active N	Control Events	Control N	Relative risk [95% CI]
Unstable CAD – Unrevascularized post–MI					
TOPS, 1992	0	42	1	45	0.36 [0.01, 8.52]
DAKIK, 1998	1	21	1	23	1.10 [0.07, 16.43]
Horie, 1998	1	44	5	39	0.18 [0.02, 1.45]
ALKK, 2003	6	149	17	151	0.36 [0.15, 0.88]
DECOPI, 2004	8	109	9	103	0.84 [0.34, 2.09]
SWISSI 2, 2007	6	96	22	105	0.30 [0.13, 0.70]
VIAMI, 2012	2	106	3	110	0.69 [0.12, 4.06]
Unrevascularized post–MI studies (**p = 0.00**, Q = 4.33, df = 6, p for heterogeneity = 0.63; I^2 = 5.4%)					0.44 [0.27, 0.73]
Unstable CAD – Multivessel disease following STEMI					
Help–AMI, 2009	1	52	0	17	1.02 [0.04, 23.91]
Politi, 2010	10	130	13	84	0.50 [0.23, 1.08]
Dambrink, 2012	2	79	0	40	2.56 [0.13, 52.14]
PRAMI, 2013	12	234	16	231	0.74 [0.36, 1.53]
CvLPRIT, 2015	4	150	10	146	0.39 [0.12, 1.21]
DANAMI 3, 2015	15	313	11	314	1.37 [0.64, 2.93]
Zhang, 2015	13	215	15	213	0.86 [0.42, 1.76]
Hamza, 2016	1	50	4	50	0.25 [0.03, 2.16]
Compare ACUTE, 2017	4	295	10	590	0.80 [0.25, 2.53]
Complete, 2019	96	2016	106	2025	0.91 [0.70, 1.19]
Multivessel disease following STEMI studies (**p = 0.11**, Q = 7.30, df = 9, p for heterogeneity = 0.61; I^2 = 0.0%)					0.84 [0.69, 1.04]
Unstable CAD – NSTEMI					
TIMI IIIB, 1994	18	740	18	733	0.99 [0.52, 1.89]
FRISC II, 2000	27	1222	48	1234	0.57 [0.36, 0.90]
TRUCS, 2000	3	76	9	72	0.32 [0.09, 1.12]
TACTICS 18, 2001	37	1114	39	1106	0.94 [0.61, 1.47]
RITA 3, 2002	60	895	72	915	0.85 [0.61, 1.19]
VINO, 2002	2	64	9	67	0.23 [0.05, 1.04]
ICTUS, 2005	15	604	15	596	0.99 [0.49, 2.00]
Savonitto, 2012	19	154	22	159	0.89 [0.50, 1.58]
After 80, 2016	57	229	62	228	0.92 [0.67, 1.25]
Sanchis, 2016	22	52	26	54	0.88 [0.58, 1.34]
NSTEMI studies (**p = 0.02**, Q = 8.95, df = 9.00, p for heterogeneity = 0.44; I^2 = 0.0%)					0.84 [0.72, 0.97]
**Unstable CAD studies (p = 0.000, Q = 27.24, df = 26.00, p for heterogeneity = 0.40; I^2 = 0.0%)**					0.81 [0.72, 0.91]
Stable CAD					
ACME 1, 1992	0	105	1	107	0.34 [0.01, 8.24]
MASS 1, 1995	1	72	0	72	3.00 [0.12, 72.44]
ACME 2, 1997	25	166	25	162	0.98 [0.59, 1.63]
RITA 2, 1997	11	504	7	514	1.60 [0.63, 4.10]
ACIP, 1997	2	192	20	366	0.19 [0.05, 0.81]
AVERT, 1999	1	177	1	164	0.93 [0.06, 14.69]
DEFER, 2001	2	90	4	91	0.51 [0.09, 2.69]
TIME, 2001	13	153	6	148	2.10 [0.82, 5.37]
MASS 2, 2004	9	205	3	203	2.97 [0.82, 10.82]
Hambrecht, 2004	0	50	0	51	1.02 [0.02, 50.41]
COURAGE, 2007	85	1149	95	1138	0.89 [0.67, 1.17]
JSAP, 2008	6	188	7	191	0.87 [0.30, 2.54]
BARI 2D, 2009	102	798	96	807	1.07 [0.83, 1.39]
FAME 2, 2012	1	447	3	441	0.33 [0.03, 3.15]
**Stable CAD studies (p = 0.98, Q = 14.69, df = 13.00, p for heterogeneity = 0.33; I^2 = 0.0%)**					1.00 [0.85, 1.18]

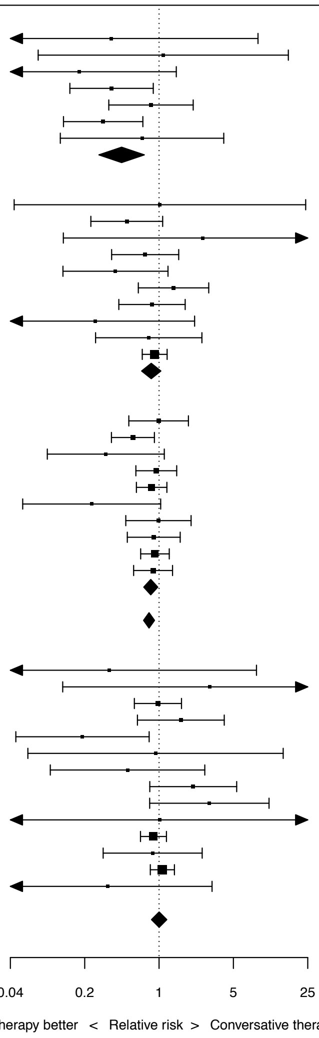

Online Figure 45. Sensitivity analysis for primary outcome of all-cause mortality excluding the Politi trial

Study and Year	Active Events	N	Control Events	N	Relative risk [95% CI]
Unstable CAD – Unrevascularized post–MI					
TOPS, 1992	0	42	1	45	0.36 [0.01, 8.52]
DAKIK, 1998	1	21	1	23	1.10 [0.07, 16.43]
Horie, 1998	1	44	5	39	0.18 [0.02, 1.45]
ALKK, 2003	6	149	17	151	0.36 [0.15, 0.88]
DECOPI, 2004	8	109	9	103	0.84 [0.34, 2.09]
OAT, 2006	87	1082	84	1084	1.04 [0.78, 1.38]
SWISSI 2, 2007	6	96	22	105	0.30 [0.13, 0.70]
VIAMI, 2012	2	106	3	110	0.69 [0.12, 4.06]
Unrevascularized post–MI studies (**p = 0.04**, Q = 13.52, df = 7, p for heterogeneity = 0.06; I^2 = 48.3%)					0.58 [0.34, 0.98]
Unstable CAD – Multivessel disease following STEMI					
Help–AMI, 2009	1	52	0	17	1.02 [0.04, 23.91]
Dambrink, 2012	2	79	0	40	2.56 [0.13, 52.14]
PRAMI, 2013	12	234	16	231	0.74 [0.36, 1.53]
CvLPRIT, 2015	4	150	10	146	0.39 [0.12, 1.21]
DANAMI 3, 2015	15	313	11	314	1.37 [0.64, 2.93]
Zhang, 2015	13	215	15	213	0.86 [0.42, 1.76]
Hamza, 2016	1	50	4	50	0.25 [0.03, 2.16]
Compare ACUTE, 2017	4	295	10	590	0.80 [0.25, 2.53]
Complete, 2019	96	2016	106	2025	0.91 [0.70, 1.19]
Multivessel disease following STEMI studies (**p = 0.25**, Q = 5.37, df = 8, p for heterogeneity = 0.72; I^2 = 0.0%)					0.88 [0.71, 1.09]
Unstable CAD – NSTEMI					
TIMI IIIB, 1994	18	740	18	733	0.99 [0.52, 1.89]
FRISC II, 2000	27	1222	48	1234	0.57 [0.36, 0.90]
TRUCS, 2000	3	76	9	72	0.32 [0.09, 1.12]
TACTICS 18, 2001	37	1114	39	1106	0.94 [0.61, 1.47]
RITA 3, 2002	60	895	72	915	0.85 [0.61, 1.19]
VINO, 2002	2	64	9	67	0.23 [0.05, 1.04]
ICTUS, 2005	15	604	15	596	0.99 [0.49, 2.00]
Savonitto, 2012	19	154	22	159	0.89 [0.50, 1.58]
After 80, 2016	57	229	62	228	0.92 [0.67, 1.25]
Sanchis, 2016	22	52	26	54	0.88 [0.58, 1.34]
NSTEMI studies (**p = 0.02**, Q = 8.95, df = 9.00, p for heterogeneity = 0.44; I^2 = 0.0%)					0.84 [0.72, 0.97]
**Unstable CAD studies (p = 0.003, Q = 28.04, df = 26.00, p for heterogeneity = 0.36; I^2 = 0.0%)**					0.84 [0.76, 0.94]
Stable CAD					
ACME 1, 1992	0	105	1	107	0.34 [0.01, 8.24]
MASS 1, 1995	1	72	0	72	3.00 [0.12, 72.44]
ACME 2, 1997	25	166	25	162	0.98 [0.59, 1.63]
RITA 2, 1997	11	504	7	514	1.60 [0.63, 4.10]
ACIP, 1997	2	192	20	366	0.19 [0.05, 0.81]
AVERT, 1999	1	177	1	164	0.93 [0.06, 14.69]
DEFER, 2001	2	90	4	91	0.51 [0.09, 2.69]
TIME, 2001	13	153	6	148	2.10 [0.82, 5.37]
MASS 2, 2004	9	205	3	203	2.97 [0.82, 10.82]
Hambrecht, 2004	0	50	0	51	1.02 [0.02, 50.41]
COURAGE, 2007	85	1149	95	1138	0.89 [0.67, 1.17]
JSAP, 2008	6	188	7	191	0.87 [0.30, 2.54]
BARI 2D, 2009	102	798	96	807	1.07 [0.83, 1.39]
FAME 2, 2012	1	447	3	441	0.33 [0.03, 3.15]
**Stable CAD studies (p = 0.98, Q = 14.69, df = 13.00, p for heterogeneity = 0.33; I^2 = 0.0%)**					1.00 [0.85, 1.18]

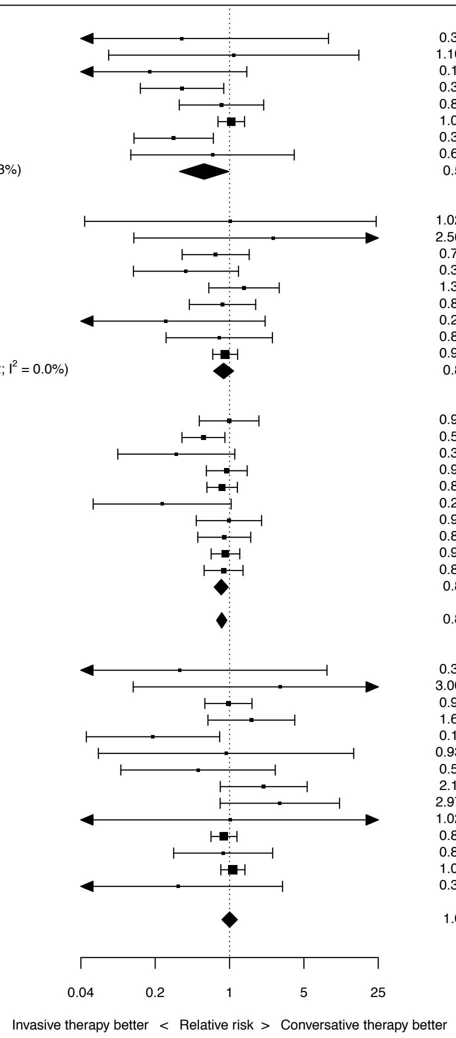

Online Figure 46. Sensitivity analysis for primary outcome of all-cause mortality excluding the PRAMI trial

Study and Year	Active Events	Active N	Control Events	Control N	Relative risk [95% CI]
Unstable CAD – Unrevascularized post–MI					
TOPS, 1992	0	42	1	45	0.36 [0.01, 8.52]
DAKIK, 1998	1	21	1	23	1.10 [0.07, 16.43]
Horie, 1998	1	44	5	39	0.18 [0.02, 1.45]
ALKK, 2003	6	149	17	151	0.36 [0.15, 0.88]
DECOPI, 2004	8	109	9	103	0.84 [0.34, 2.09]
OAT, 2006	87	1082	84	1084	1.04 [0.78, 1.38]
SWISSI 2, 2007	6	96	22	105	0.30 [0.13, 0.70]
VIAMI, 2012	2	106	3	110	0.69 [0.12, 4.06]
Unrevascularized post–MI studies (**p = 0.04**, Q = 13.52, df = 7, p for heterogeneity = 0.06; I^2 = 48.3%)					0.58 [0.34, 0.98]
Unstable CAD – Multivessel disease following STEMI					
Help–AMI, 2009	1	52	0	17	1.02 [0.04, 23.91]
Politi, 2010	10	130	13	84	0.50 [0.23, 1.08]
Dambrink, 2012	2	79	0	40	2.56 [0.13, 52.14]
CvLPRIT, 2015	4	150	10	146	0.39 [0.12, 1.21]
DANAMI 3, 2015	15	313	11	314	1.37 [0.64, 2.93]
Zhang, 2015	13	215	15	213	0.86 [0.42, 1.76]
Hamza, 2016	1	50	4	50	0.25 [0.03, 2.16]
Compare ACUTE, 2017	4	295	10	590	0.80 [0.25, 2.53]
Complete, 2019	96	2016	106	2025	0.91 [0.70, 1.19]
Multivessel disease following STEMI studies (**p = 0.16**, Q = 7.16, df = 8, p for heterogeneity = 0.52; I^2 = 0.0%)					0.85 [0.69, 1.06]
Unstable CAD – NSTEMI					
TIMI IIIB, 1994	18	740	18	733	0.99 [0.52, 1.89]
FRISC II, 2000	27	1222	48	1234	0.57 [0.36, 0.90]
TRUCS, 2000	3	76	9	72	0.32 [0.09, 1.12]
TACTICS 18, 2001	37	1114	39	1106	0.94 [0.61, 1.47]
RITA 3, 2002	60	895	72	915	0.85 [0.61, 1.19]
VINO, 2002	2	64	9	67	0.23 [0.05, 1.04]
ICTUS, 2005	15	604	15	596	0.99 [0.49, 2.00]
Savonitto, 2012	19	154	22	159	0.89 [0.50, 1.58]
After 80, 2016	57	229	62	228	0.92 [0.67, 1.25]
Sanchis, 2016	22	52	26	54	0.88 [0.58, 1.34]
NSTEMI studies (**p = 0.02**, Q = 8.95, df = 9.00, p for heterogeneity = 0.44; I^2 = 0.0%)					0.84 [0.72, 0.97]
**Unstable CAD studies (p = 0.002, Q = 29.68, df = 26.00, p for heterogeneity = 0.28; I^2 = 0.0%)**					0.84 [0.75, 0.94]
Stable CAD					
ACME 1, 1992	0	105	1	107	0.34 [0.01, 8.24]
MASS 1, 1995	1	72	0	72	3.00 [0.12, 72.44]
ACME 2, 1997	25	166	25	162	0.98 [0.59, 1.63]
RITA 2, 1997	11	504	7	514	1.60 [0.63, 4.10]
ACIP, 1997	2	192	20	366	0.19 [0.05, 0.81]
AVERT, 1999	1	177	1	164	0.93 [0.06, 14.69]
DEFER, 2001	2	90	4	91	0.51 [0.09, 2.69]
TIME, 2001	13	153	6	148	2.10 [0.82, 5.37]
MASS 2, 2004	9	205	3	203	2.97 [0.82, 10.82]
Hambrecht, 2004	0	50	0	51	1.02 [0.02, 50.41]
COURAGE, 2007	85	1149	95	1138	0.89 [0.67, 1.17]
JSAP, 2008	6	188	7	191	0.87 [0.30, 2.54]
BARI 2D, 2009	102	798	96	807	1.07 [0.83, 1.39]
FAME 2, 2012	1	447	3	441	0.33 [0.03, 3.15]
**Stable CAD studies (p = 0.98, Q = 14.69, df = 13.00, p for heterogeneity = 0.33; I^2 = 0.0%)**					1.00 [0.85, 1.18]

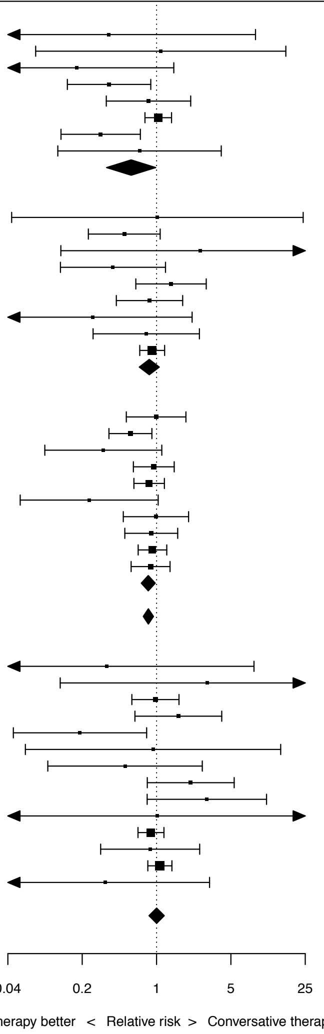

Online Figure 47. Sensitivity analysis for primary outcome of all-cause mortality excluding the RITA 2 trial

Study and Year	Active Events	Active N	Control Events	Control N	Relative risk [95% CI]
Unstable CAD – Unrevascularized post–MI					
TOPS, 1992	0	42	1	45	0.36 [0.01, 8.52]
DAKIK, 1998	1	21	1	23	1.10 [0.07, 16.43]
Horie, 1998	1	44	5	39	0.18 [0.02, 1.45]
ALKK, 2003	6	149	17	151	0.36 [0.15, 0.88]
DECOPI, 2004	8	109	9	103	0.84 [0.34, 2.09]
OAT, 2006	87	1082	84	1084	1.04 [0.78, 1.38]
SWISSI 2, 2007	6	96	22	105	0.30 [0.13, 0.70]
VIAMI, 2012	2	106	3	110	0.69 [0.12, 4.06]
Unrevascularized post–MI studies (**p = 0.04**, Q = 13.52, df = 7, p for heterogeneity = 0.06; I^2 = 48.3%)					0.58 [0.34, 0.98]
Unstable CAD – Multivessel disease following STEMI					
Help–AMI, 2009	1	52	0	17	1.02 [0.04, 23.91]
Politi, 2010	10	130	13	84	0.50 [0.23, 1.08]
Dambrink, 2012	2	79	0	40	2.56 [0.13, 52.14]
PRAMI, 2013	12	234	16	231	0.74 [0.36, 1.53]
CvLPRIT, 2015	4	150	10	146	0.39 [0.12, 1.21]
DANAMI 3, 2015	15	313	11	314	1.37 [0.64, 2.93]
Zhang, 2015	13	215	15	213	0.86 [0.42, 1.76]
Hamza, 2016	1	50	4	50	0.25 [0.03, 2.16]
Compare ACUTE, 2017	4	295	10	590	0.80 [0.25, 2.53]
Complete, 2019	96	2016	106	2025	0.91 [0.70, 1.19]
Multivessel disease following STEMI studies (**p = 0.11**, Q = 7.30, df = 9, p for heterogeneity = 0.61; I^2 = 0.0%)					0.84 [0.69, 1.04]
Unstable CAD – NSTEMI					
TIMI IIIB, 1994	18	740	18	733	0.99 [0.52, 1.89]
FRISC II, 2000	27	1222	48	1234	0.57 [0.36, 0.90]
TRUCS, 2000	3	76	9	72	0.32 [0.09, 1.12]
TACTICS 18, 2001	37	1114	39	1106	0.94 [0.61, 1.47]
RITA 3, 2002	60	895	72	915	0.85 [0.61, 1.19]
VINO, 2002	2	64	9	67	0.23 [0.05, 1.04]
ICTUS, 2005	15	604	15	596	0.99 [0.49, 2.00]
Savonitto, 2012	19	154	22	159	0.89 [0.50, 1.58]
After 80, 2016	57	229	62	228	0.92 [0.67, 1.25]
Sanchis, 2016	22	52	26	54	0.88 [0.58, 1.34]
NSTEMI studies (**p = 0.02**, Q = 8.95, df = 9.00, p for heterogeneity = 0.44; I^2 = 0.0%)					0.84 [0.72, 0.97]
**Unstable CAD studies (p = 0.001, Q = 29.79, df = 27.00, p for heterogeneity = 0.32; I^2 = 0.0%)**					0.84 [0.75, 0.93]
Stable CAD					
ACME 1, 1992	0	105	1	107	0.34 [0.01, 8.24]
MASS 1, 1995	1	72	0	72	3.00 [0.12, 72.44]
ACME 2, 1997	25	166	25	162	0.98 [0.59, 1.63]
ACIP, 1997	2	192	20	366	0.19 [0.05, 0.81]
AVERT, 1999	1	177	1	164	0.93 [0.06, 14.69]
DEFER, 2001	2	90	4	91	0.51 [0.09, 2.69]
TIME, 2001	13	153	6	148	2.10 [0.82, 5.37]
MASS 2, 2004	9	205	3	203	2.97 [0.82, 10.82]
Hambrecht, 2004	0	50	0	51	1.02 [0.02, 50.41]
COURAGE, 2007	85	1149	95	1138	0.89 [0.67, 1.17]
JSAP, 2008	6	188	7	191	0.87 [0.30, 2.54]
BARI 2D, 2009	102	798	96	807	1.07 [0.83, 1.39]
FAME 2, 2012	1	447	3	441	0.33 [0.03, 3.15]
**Stable CAD studies (p = 0.88, Q = 13.69, df = 12.00, p for heterogeneity = 0.32; I^2 = 0.0%)**					0.99 [0.83, 1.17]

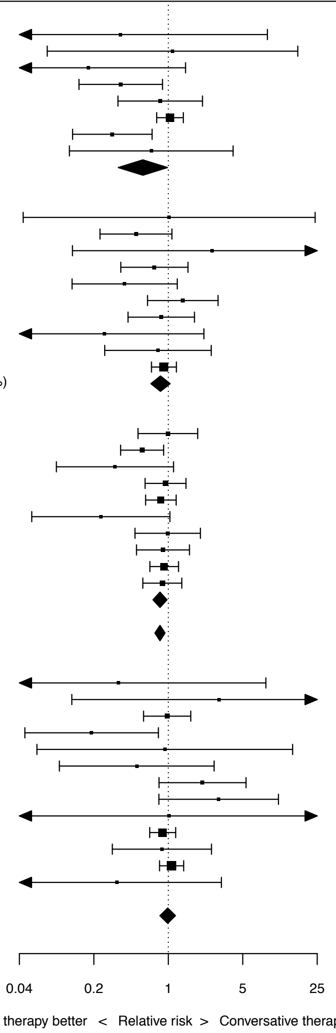

Online Figure 48. Sensitivity analysis for primary outcome of all-cause mortality excluding the RITA 3 trial

Study and Year	Active Events	Active N	Control Events	Control N	Relative risk [95% CI]
Unstable CAD – Unrevascularized post–MI					
TOPS, 1992	0	42	1	45	0.36 [0.01, 8.52]
DAKIK, 1998	1	21	1	23	1.10 [0.07, 16.43]
Horie, 1998	1	44	5	39	0.18 [0.02, 1.45]
ALKK, 2003	6	149	17	151	0.36 [0.15, 0.88]
DECOPI, 2004	8	109	9	103	0.84 [0.34, 2.09]
OAT, 2006	87	1082	84	1084	1.04 [0.78, 1.38]
SWISSI 2, 2007	6	96	22	105	0.30 [0.13, 0.70]
VIAMI, 2012	2	106	3	110	0.69 [0.12, 4.06]
Unrevascularized post–MI studies (**p = 0.04**, Q = 13.52, df = 7, p for heterogeneity = 0.06; I^2 = 48.3%)					0.58 [0.34, 0.98]
Unstable CAD – Multivessel disease following STEMI					
Help–AMI, 2009	1	52	0	17	1.02 [0.04, 23.91]
Politi, 2010	10	130	13	84	0.50 [0.23, 1.08]
Dambrink, 2012	2	79	0	40	2.56 [0.13, 52.14]
PRAMI, 2013	12	234	16	231	0.74 [0.36, 1.53]
CvLPRIT, 2015	4	150	10	146	0.39 [0.12, 1.21]
DANAMI 3, 2015	15	313	11	314	1.37 [0.64, 2.93]
Zhang, 2015	13	215	15	213	0.86 [0.42, 1.76]
Hamza, 2016	1	50	4	50	0.25 [0.03, 2.16]
Compare ACUTE, 2017	4	295	10	590	0.80 [0.25, 2.53]
Complete, 2019	96	2016	106	2025	0.91 [0.70, 1.19]
Multivessel disease following STEMI studies (**p = 0.11**, Q = 7.30, df = 9, p for heterogeneity = 0.61; I^2 = 0.0%)					0.84 [0.69, 1.04]
Unstable CAD – NSTEMI					
TIMI IIIB, 1994	18	740	18	733	0.99 [0.52, 1.89]
FRISC II, 2000	27	1222	48	1234	0.57 [0.36, 0.90]
TRUCS, 2000	3	76	9	72	0.32 [0.09, 1.12]
TACTICS 18, 2001	37	1114	39	1106	0.94 [0.61, 1.47]
VINO, 2002	2	64	9	67	0.23 [0.05, 1.04]
ICTUS, 2005	15	604	15	596	0.99 [0.49, 2.00]
Savonitto, 2012	19	154	22	159	0.89 [0.50, 1.58]
After 80, 2016	57	229	62	228	0.92 [0.67, 1.25]
Sanchis, 2016	22	52	26	54	0.88 [0.58, 1.34]
NSTEMI studies (**p = 0.03**, Q = 8.93, df = 8.00, p for heterogeneity = 0.35; I^2 = 0.0%)					0.83 [0.70, 0.99]
**Unstable CAD studies (p = 0.002, Q = 29.77, df = 26.00, p for heterogeneity = 0.28; I^2 = 7.9%)**					0.82 [0.72, 0.93]
Stable CAD					
ACME 1, 1992	0	105	1	107	0.34 [0.01, 8.24]
MASS 1, 1995	1	72	0	72	3.00 [0.12, 72.44]
ACME 2, 1997	25	166	25	162	0.98 [0.59, 1.63]
RITA 2, 1997	11	504	7	514	1.60 [0.63, 4.10]
ACIP, 1997	2	192	20	366	0.19 [0.05, 0.81]
AVERT, 1999	1	177	1	164	0.93 [0.06, 14.69]
DEFER, 2001	2	90	4	91	0.51 [0.09, 2.69]
TIME, 2001	13	153	6	148	2.10 [0.82, 5.37]
MASS 2, 2004	9	205	3	203	2.97 [0.82, 10.82]
Hambrecht, 2004	0	50	0	51	1.02 [0.02, 50.41]
COURAGE, 2007	85	1149	95	1138	0.89 [0.67, 1.17]
JSAP, 2008	6	188	7	191	0.87 [0.30, 2.54]
BARI 2D, 2009	102	798	96	807	1.07 [0.83, 1.39]
FAME 2, 2012	1	447	3	441	0.33 [0.03, 3.15]
**Stable CAD studies (p = 0.98, Q = 14.69, df = 13.00, p for heterogeneity = 0.33; I^2 = 0.0%)**					1.00 [0.85, 1.18]

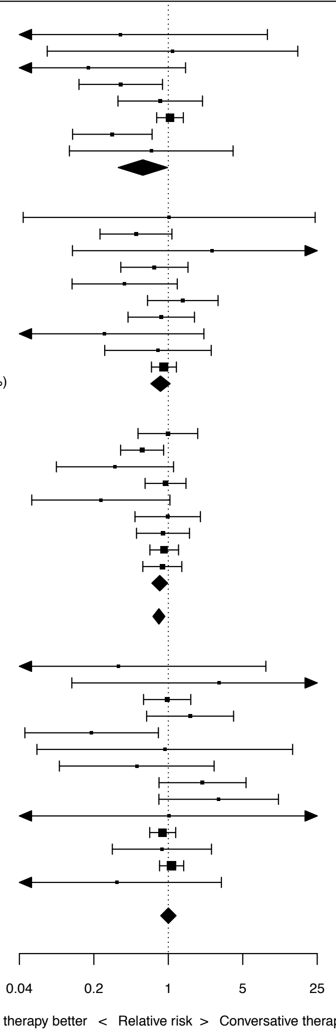

Online Figure 49. Sensitivity analysis for primary outcome of all-cause mortality excluding the Sanchis trial

Study and Year	Active Events	Active N	Control Events	Control N	Relative risk [95% CI]
Unstable CAD – Unrevascularized post–MI					
TOPS, 1992	0	42	1	45	0.36 [0.01, 8.52]
DAKIK, 1998	1	21	1	23	1.10 [0.07, 16.43]
Horie, 1998	1	44	5	39	0.18 [0.02, 1.45]
ALKK, 2003	6	149	17	151	0.36 [0.15, 0.88]
DECOPI, 2004	8	109	9	103	0.84 [0.34, 2.09]
OAT, 2006	87	1082	84	1084	1.04 [0.78, 1.38]
SWISSI 2, 2007	6	96	22	105	0.30 [0.13, 0.70]
VIAMI, 2012	2	106	3	110	0.69 [0.12, 4.06]
Unrevascularized post–MI studies (**p = 0.04**, Q = 13.52, df = 7, p for heterogeneity = 0.06; I^2 = 48.3%)					0.58 [0.34, 0.98]
Unstable CAD – Multivessel disease following STEMI					
Help–AMI, 2009	1	52	0	17	1.02 [0.04, 23.91]
Politi, 2010	10	130	13	84	0.50 [0.23, 1.08]
Dambrink, 2012	2	79	0	40	2.56 [0.13, 52.14]
PRAMI, 2013	12	234	16	231	0.74 [0.36, 1.53]
CvLPRIT, 2015	4	150	10	146	0.39 [0.12, 1.21]
DANAMI 3, 2015	15	313	11	314	1.37 [0.64, 2.93]
Zhang, 2015	13	215	15	213	0.86 [0.42, 1.76]
Hamza, 2016	1	50	4	50	0.25 [0.03, 2.16]
Compare ACUTE, 2017	4	295	10	590	0.80 [0.25, 2.53]
Complete, 2019	96	2016	106	2025	0.91 [0.70, 1.19]
Multivessel disease following STEMI studies (**p = 0.11**, Q = 7.30, df = 9, p for heterogeneity = 0.61; I^2 = 0.0%)					0.84 [0.69, 1.04]
Unstable CAD – NSTEMI					
TIMI IIIB, 1994	18	740	18	733	0.99 [0.52, 1.89]
FRISC II, 2000	27	1222	48	1234	0.57 [0.36, 0.90]
TRUCS, 2000	3	76	9	72	0.32 [0.09, 1.12]
TACTICS 18, 2001	37	1114	39	1106	0.94 [0.61, 1.47]
RITA 3, 2002	60	895	72	915	0.85 [0.61, 1.19]
VINO, 2002	2	64	9	67	0.23 [0.05, 1.04]
ICTUS, 2005	15	604	15	596	0.99 [0.49, 2.00]
Savonitto, 2012	19	154	22	159	0.89 [0.50, 1.58]
After 80, 2016	57	229	62	228	0.92 [0.67, 1.25]
NSTEMI studies (**p = 0.02**, Q = 8.88, df = 8.00, p for heterogeneity = 0.35; I^2 = 0.0%)					0.83 [0.71, 0.98]
**Unstable CAD studies (p = 0.002, Q = 29.73, df = 26.00, p for heterogeneity = 0.28; I^2 = 5.1%)**					0.82 [0.73, 0.93]
Stable CAD					
ACME 1, 1992	0	105	1	107	0.34 [0.01, 8.24]
MASS 1, 1995	1	72	0	72	3.00 [0.12, 72.44]
ACME 2, 1997	25	166	25	162	0.98 [0.59, 1.63]
RITA 2, 1997	11	504	7	514	1.60 [0.63, 4.10]
ACIP, 1997	2	192	20	366	0.19 [0.05, 0.81]
AVERT, 1999	1	177	1	164	0.93 [0.06, 14.69]
DEFER, 2001	2	90	4	91	0.51 [0.09, 2.69]
TIME, 2001	13	153	6	148	2.10 [0.82, 5.37]
MASS 2, 2004	9	205	3	203	2.97 [0.82, 10.82]
Hambrecht, 2004	0	50	0	51	1.02 [0.02, 50.41]
COURAGE, 2007	85	1149	95	1138	0.89 [0.67, 1.17]
JSAP, 2008	6	188	7	191	0.87 [0.30, 2.54]
BARI 2D, 2009	102	798	96	807	1.07 [0.83, 1.39]
FAME 2, 2012	1	447	3	441	0.33 [0.03, 3.15]
**Stable CAD studies (p = 0.98, Q = 14.69, df = 13.00, p for heterogeneity = 0.33; I^2 = 0.0%)**					1.00 [0.85, 1.18]

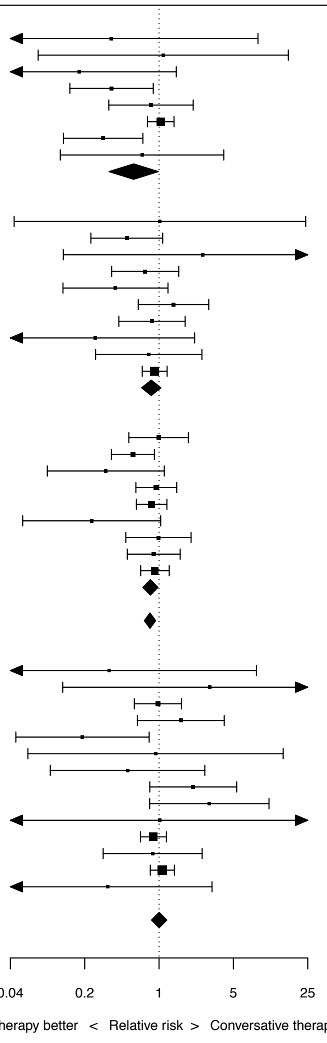

Online Figure 50. Sensitivity analysis for primary outcome of all-cause mortality excluding the Savonitto trial

Study and Year	Active Events	N	Control Events	N	Relative risk [95% CI]
Unstable CAD – Unrevascularized post–MI					
TOPS, 1992	0	42	1	45	0.36 [0.01, 8.52]
DAKIK, 1998	1	21	1	23	1.10 [0.07, 16.43]
Horie, 1998	1	44	5	39	0.18 [0.02, 1.45]
ALKK, 2003	6	149	17	151	0.36 [0.15, 0.88]
DECOPI, 2004	8	109	9	103	0.84 [0.34, 2.09]
OAT, 2006	87	1082	84	1084	1.04 [0.78, 1.38]
SWISSI 2, 2007	6	96	22	105	0.30 [0.13, 0.70]
VIAMI, 2012	2	106	3	110	0.69 [0.12, 4.06]
Unrevascularized post–MI studies (**p = 0.04**, Q = 13.52, df = 7, p for heterogeneity = 0.06; I^2 = 48.3%)					0.58 [0.34, 0.98]
Unstable CAD – Multivessel disease following STEMI					
Help–AMI, 2009	1	52	0	17	1.02 [0.04, 23.91]
Politi, 2010	10	130	13	84	0.50 [0.23, 1.08]
Dambrink, 2012	2	79	0	40	2.56 [0.13, 52.14]
PRAMI, 2013	12	234	16	231	0.74 [0.36, 1.53]
CvLPRIT, 2015	4	150	10	146	0.39 [0.12, 1.21]
DANAMI 3, 2015	15	313	11	314	1.37 [0.64, 2.93]
Zhang, 2015	13	215	15	213	0.86 [0.42, 1.76]
Hamza, 2016	1	50	4	50	0.25 [0.03, 2.16]
Compare ACUTE, 2017	4	295	10	590	0.80 [0.25, 2.53]
Complete, 2019	96	2016	106	2025	0.91 [0.70, 1.19]
Multivessel disease following STEMI studies (**p = 0.11**, Q = 7.30, df = 9, p for heterogeneity = 0.61; I^2 = 0.0%)					0.84 [0.69, 1.04]
Unstable CAD – NSTEMI					
TIMI IIIB, 1994	18	740	18	733	0.99 [0.52, 1.89]
FRISC II, 2000	27	1222	48	1234	0.57 [0.36, 0.90]
TRUCS, 2000	3	76	9	72	0.32 [0.09, 1.12]
TACTICS 18, 2001	37	1114	39	1106	0.94 [0.61, 1.47]
RITA 3, 2002	60	895	72	915	0.85 [0.61, 1.19]
VINO, 2002	2	64	9	67	0.23 [0.05, 1.04]
ICTUS, 2005	15	604	15	596	0.99 [0.49, 2.00]
After 80, 2016	57	229	62	228	0.92 [0.67, 1.25]
Sanchis, 2016	22	52	26	54	0.88 [0.58, 1.34]
NSTEMI studies (**p = 0.02**, Q = 8.89, df = 8.00, p for heterogeneity = 0.35; I^2 = 0.0%)					0.83 [0.71, 0.97]
**Unstable CAD studies (p = 0.001, Q = 29.74, df = 26.00, p for heterogeneity = 0.28; I^2 = 1.9%)**					0.83 [0.74, 0.93]
Stable CAD					
ACME 1, 1992	0	105	1	107	0.34 [0.01, 8.24]
MASS 1, 1995	1	72	0	72	3.00 [0.12, 72.44]
ACME 2, 1997	25	166	25	162	0.98 [0.59, 1.63]
RITA 2, 1997	11	504	7	514	1.60 [0.63, 4.10]
ACIP, 1997	2	192	20	366	0.19 [0.05, 0.81]
AVERT, 1999	1	177	1	164	0.93 [0.06, 14.69]
DEFER, 2001	2	90	4	91	0.51 [0.09, 2.69]
TIME, 2001	13	153	6	148	2.10 [0.82, 5.37]
MASS 2, 2004	9	205	3	203	2.97 [0.82, 10.82]
Hambrecht, 2004	0	50	0	51	1.02 [0.02, 50.41]
COURAGE, 2007	85	1149	95	1138	0.89 [0.67, 1.17]
JSAP, 2008	6	188	7	191	0.87 [0.30, 2.54]
BARI 2D, 2009	102	798	96	807	1.07 [0.83, 1.39]
FAME 2, 2012	1	447	3	441	0.33 [0.03, 3.15]
**Stable CAD studies (p = 0.98, Q = 14.69, df = 13.00, p for heterogeneity = 0.33; I^2 = 0.0%)**					1.00 [0.85, 1.18]

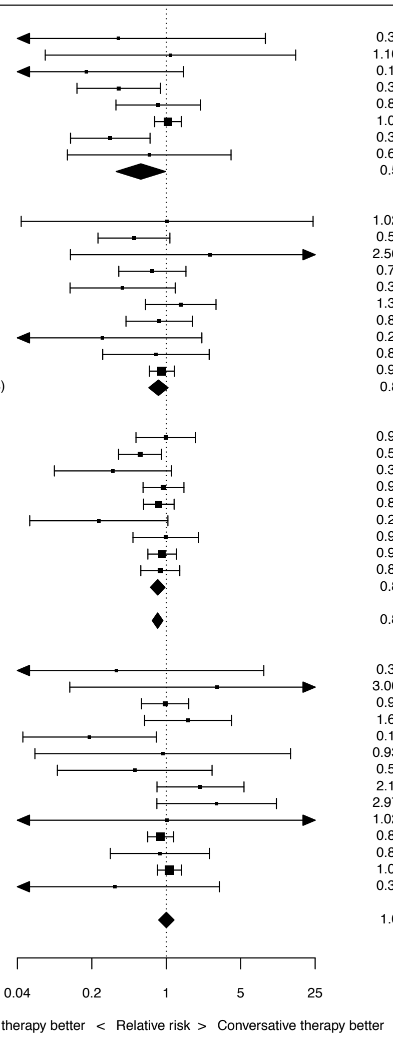

Online Figure 51. Sensitivity analysis for primary outcome of all-cause mortality excluding the SWISSI 2 trial

Study and Year	Active Events	Active N	Control Events	Control N	Relative risk [95% CI]
Unstable CAD – Unrevascularized post–MI					
TOPS, 1992	0	42	1	45	0.36 [0.01, 8.52]
DAKIK, 1998	1	21	1	23	1.10 [0.07, 16.43]
Horie, 1998	1	44	5	39	0.18 [0.02, 1.45]
ALKK, 2003	6	149	17	151	0.36 [0.15, 0.88]
DECOPI, 2004	8	109	9	103	0.84 [0.34, 2.09]
OAT, 2006	87	1082	84	1084	1.04 [0.78, 1.38]
VIAMI, 2012	2	106	3	110	0.69 [0.12, 4.06]
Unrevascularized post–MI studies (**p = 0.17**, Q = 7.70, df = 6, p for heterogeneity = 0.26; I^2 = 34.9%)					0.69 [0.41, 1.17]
Unstable CAD – Multivessel disease following STEMI					
Help–AMI, 2009	1	52	0	17	1.02 [0.04, 23.91]
Politi, 2010	10	130	13	84	0.50 [0.23, 1.08]
Dambrink, 2012	2	79	0	40	2.56 [0.13, 52.14]
PRAMI, 2013	12	234	16	231	0.74 [0.36, 1.53]
CvLPRIT, 2015	4	150	10	146	0.39 [0.12, 1.21]
DANAMI 3, 2015	15	313	11	314	1.37 [0.64, 2.93]
Zhang, 2015	13	215	15	213	0.86 [0.42, 1.76]
Hamza, 2016	1	50	4	50	0.25 [0.03, 2.16]
Compare ACUTE, 2017	4	295	10	590	0.80 [0.25, 2.53]
Complete, 2019	96	2016	106	2025	0.91 [0.70, 1.19]
Multivessel disease following STEMI studies (**p = 0.11**, Q = 7.30, df = 9, p for heterogeneity = 0.61; I^2 = 0.0%)					0.84 [0.69, 1.04]
Unstable CAD – NSTEMI					
TIMI IIIB, 1994	18	740	18	733	0.99 [0.52, 1.89]
FRISC II, 2000	27	1222	48	1234	0.57 [0.36, 0.90]
TRUCS, 2000	3	76	9	72	0.32 [0.09, 1.12]
TACTICS 18, 2001	37	1114	39	1106	0.94 [0.61, 1.47]
RITA 3, 2002	60	895	72	915	0.85 [0.61, 1.19]
VINO, 2002	2	64	9	67	0.23 [0.05, 1.04]
ICTUS, 2005	15	604	15	596	0.99 [0.49, 2.00]
Savonitto, 2012	19	154	22	159	0.89 [0.50, 1.58]
After 80, 2016	57	229	62	228	0.92 [0.67, 1.25]
Sanchis, 2016	22	52	26	54	0.88 [0.58, 1.34]
NSTEMI studies (**p = 0.02**, Q = 8.95, df = 9.00, p for heterogeneity = 0.44; I^2 = 0.0%)					0.84 [0.72, 0.97]
**Unstable CAD studies (p = 0.004, Q = 24.18, df = 26.00, p for heterogeneity = 0.57; I^2 = 0.0%)**					0.85 [0.76, 0.95]
Stable CAD					
ACME 1, 1992	0	105	1	107	0.34 [0.01, 8.24]
MASS 1, 1995	1	72	0	72	3.00 [0.12, 72.44]
ACME 2, 1997	25	166	25	162	0.98 [0.59, 1.63]
RITA 2, 1997	11	504	7	514	1.60 [0.63, 4.10]
ACIP, 1997	2	192	20	366	0.19 [0.05, 0.81]
AVERT, 1999	1	177	1	164	0.93 [0.06, 14.69]
DEFER, 2001	2	90	4	91	0.51 [0.09, 2.69]
TIME, 2001	13	153	6	148	2.10 [0.82, 5.37]
MASS 2, 2004	9	205	3	203	2.97 [0.82, 10.82]
Hambrecht, 2004	0	50	0	51	1.02 [0.02, 50.41]
COURAGE, 2007	85	1149	95	1138	0.89 [0.67, 1.17]
JSAP, 2008	6	188	7	191	0.87 [0.30, 2.54]
BARI 2D, 2009	102	798	96	807	1.07 [0.83, 1.39]
FAME 2, 2012	1	447	3	441	0.33 [0.03, 3.15]
**Stable CAD studies (p = 0.98, Q = 14.69, df = 13.00, p for heterogeneity = 0.33; I^2 = 0.0%)**					1.00 [0.85, 1.18]

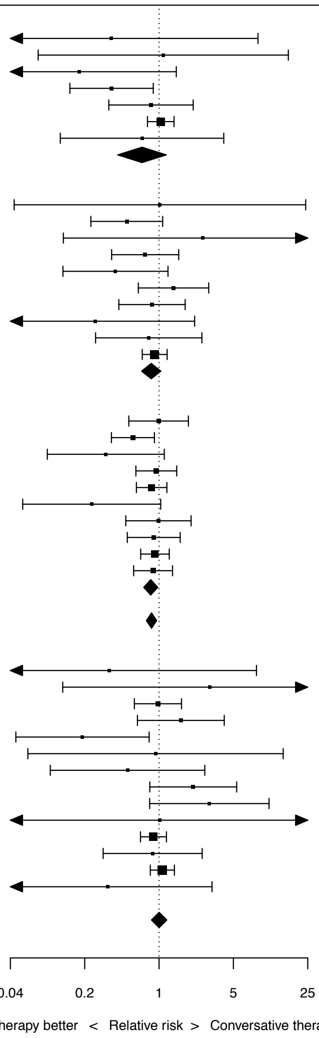

Online Figure 52. Sensitivity analysis for primary outcome of all-cause mortality excluding the TACTICS 18 trial

Study and Year	Active Events	Active N	Control Events	Control N	Relative risk [95% CI]
Unstable CAD – Unrevascularized post–MI					
TOPS, 1992	0	42	1	45	0.36 [0.01, 8.52]
DAKIK, 1998	1	21	1	23	1.10 [0.07, 16.43]
Horie, 1998	1	44	5	39	0.18 [0.02, 1.45]
ALKK, 2003	6	149	17	151	0.36 [0.15, 0.88]
DECOPI, 2004	8	109	9	103	0.84 [0.34, 2.09]
OAT, 2006	87	1082	84	1084	1.04 [0.78, 1.38]
SWISSI 2, 2007	6	96	22	105	0.30 [0.13, 0.70]
VIAMI, 2012	2	106	3	110	0.69 [0.12, 4.06]
Unrevascularized post–MI studies (**p = 0.04**, Q = 13.52, df = 7, p for heterogeneity = 0.06; I^2 = 48.3%)					0.58 [0.34, 0.98]
Unstable CAD – Multivessel disease following STEMI					
Help–AMI, 2009	1	52	0	17	1.02 [0.04, 23.91]
Politi, 2010	10	130	13	84	0.50 [0.23, 1.08]
Dambrink, 2012	2	79	0	40	2.56 [0.13, 52.14]
PRAMI, 2013	12	234	16	231	0.74 [0.36, 1.53]
CvLPRIT, 2015	4	150	10	146	0.39 [0.12, 1.21]
DANAMI 3, 2015	15	313	11	314	1.37 [0.64, 2.93]
Zhang, 2015	13	215	15	213	0.86 [0.42, 1.76]
Hamza, 2016	1	50	4	50	0.25 [0.03, 2.16]
Compare ACUTE, 2017	4	295	10	590	0.80 [0.25, 2.53]
Complete, 2019	96	2016	106	2025	0.91 [0.70, 1.19]
Multivessel disease following STEMI studies (**p = 0.11**, Q = 7.30, df = 9, p for heterogeneity = 0.61; I^2 = 0.0%)					0.84 [0.69, 1.04]
Unstable CAD – NSTEMI					
TIMI IIIB, 1994	18	740	18	733	0.99 [0.52, 1.89]
FRISC II, 2000	27	1222	48	1234	0.57 [0.36, 0.90]
TRUCS, 2000	3	76	9	72	0.32 [0.09, 1.12]
RITA 3, 2002	60	895	72	915	0.85 [0.61, 1.19]
VINO, 2002	2	64	9	67	0.23 [0.05, 1.04]
ICTUS, 2005	15	604	15	596	0.99 [0.49, 2.00]
Savonitto, 2012	19	154	22	159	0.89 [0.50, 1.58]
After 80, 2016	57	229	62	228	0.92 [0.67, 1.25]
Sanchis, 2016	22	52	26	54	0.88 [0.58, 1.34]
NSTEMI studies (**p = 0.02**, Q = 8.63, df = 8.00, p for heterogeneity = 0.37; I^2 = 0.0%)					0.82 [0.70, 0.97]
**Unstable CAD studies (p = 0.001, Q = 29.49, df = 26.00, p for heterogeneity = 0.29; I^2 = 4.8%)**					0.82 [0.73, 0.92]
Stable CAD					
ACME 1, 1992	0	105	1	107	0.34 [0.01, 8.24]
MASS 1, 1995	1	72	0	72	3.00 [0.12, 72.44]
ACME 2, 1997	25	166	25	162	0.98 [0.59, 1.63]
RITA 2, 1997	11	504	7	514	1.60 [0.63, 4.10]
ACIP, 1997	2	192	20	366	0.19 [0.05, 0.81]
AVERT, 1999	1	177	1	164	0.93 [0.06, 14.69]
DEFER, 2001	2	90	4	91	0.51 [0.09, 2.69]
TIME, 2001	13	153	6	148	2.10 [0.82, 5.37]
MASS 2, 2004	9	205	3	203	2.97 [0.82, 10.82]
Hambrecht, 2004	0	50	0	51	1.02 [0.02, 50.41]
COURAGE, 2007	85	1149	95	1138	0.89 [0.67, 1.17]
JSAP, 2008	6	188	7	191	0.87 [0.30, 2.54]
BARI 2D, 2009	102	798	96	807	1.07 [0.83, 1.39]
FAME 2, 2012	1	447	3	441	0.33 [0.03, 3.15]
**Stable CAD studies (p = 0.98, Q = 14.69, df = 13.00, p for heterogeneity = 0.33; I^2 = 0.0%)**					1.00 [0.85, 1.18]

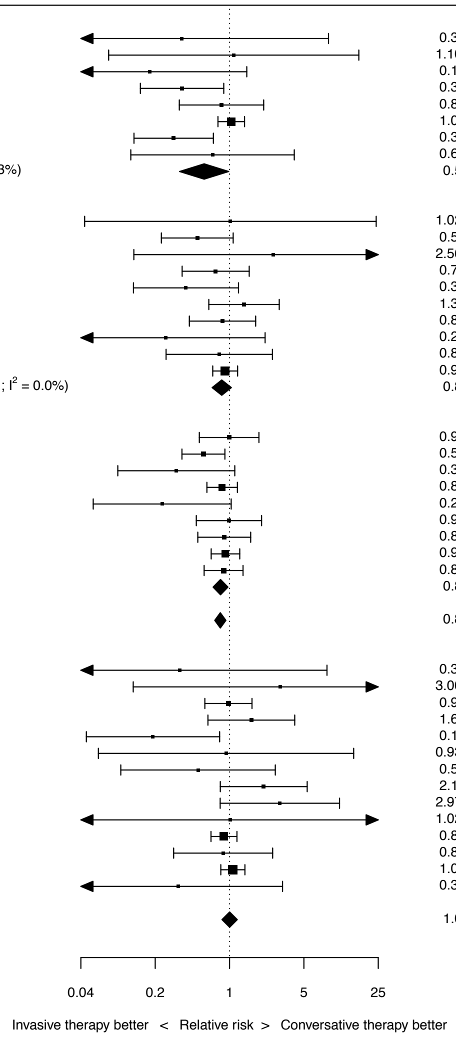

Online Figure 53. Sensitivity analysis for primary outcome of all-cause mortality excluding the TIME trial

Study and Year	Active Events	Active N	Control Events	Control N	Relative risk [95% CI]
Unstable CAD – Unrevascularized post–MI					
TOPS, 1992	0	42	1	45	0.36 [0.01, 8.52]
DAKIK, 1998	1	21	1	23	1.10 [0.07, 16.43]
Horie, 1998	1	44	5	39	0.18 [0.02, 1.45]
ALKK, 2003	6	149	17	151	0.36 [0.15, 0.88]
DECOPI, 2004	8	109	9	103	0.84 [0.34, 2.09]
OAT, 2006	87	1082	84	1084	1.04 [0.78, 1.38]
SWISSI 2, 2007	6	96	22	105	0.30 [0.13, 0.70]
VIAMI, 2012	2	106	3	110	0.69 [0.12, 4.06]
Unrevascularized post–MI studies (**p = 0.04**, Q = 13.52, df = 7, p for heterogeneity = 0.06; I^2 = 48.3%)					0.58 [0.34, 0.98]
Unstable CAD – Multivessel disease following STEMI					
Help–AMI, 2009	1	52	0	17	1.02 [0.04, 23.91]
Politi, 2010	10	130	13	84	0.50 [0.23, 1.08]
Dambrink, 2012	2	79	0	40	2.56 [0.13, 52.14]
PRAMI, 2013	12	234	16	231	0.74 [0.36, 1.53]
CvLPRIT, 2015	4	150	10	146	0.39 [0.12, 1.21]
DANAMI 3, 2015	15	313	11	314	1.37 [0.64, 2.93]
Zhang, 2015	13	215	15	213	0.86 [0.42, 1.76]
Hamza, 2016	1	50	4	50	0.25 [0.03, 2.16]
Compare ACUTE, 2017	4	295	10	590	0.80 [0.25, 2.53]
Complete, 2019	96	2016	106	2025	0.91 [0.70, 1.19]
Multivessel disease following STEMI studies (**p = 0.11**, Q = 7.30, df = 9, p for heterogeneity = 0.61; I^2 = 0.0%)					0.84 [0.69, 1.04]
Unstable CAD – NSTEMI					
TIMI IIIB, 1994	18	740	18	733	0.99 [0.52, 1.89]
FRISC II, 2000	27	1222	48	1234	0.57 [0.36, 0.90]
TRUCS, 2000	3	76	9	72	0.32 [0.09, 1.12]
TACTICS 18, 2001	37	1114	39	1106	0.94 [0.61, 1.47]
RITA 3, 2002	60	895	72	915	0.85 [0.61, 1.19]
VINO, 2002	2	64	9	67	0.23 [0.05, 1.04]
ICTUS, 2005	15	604	15	596	0.99 [0.49, 2.00]
Savonitto, 2012	19	154	22	159	0.89 [0.50, 1.58]
After 80, 2016	57	229	62	228	0.92 [0.67, 1.25]
Sanchis, 2016	22	52	26	54	0.88 [0.58, 1.34]
NSTEMI studies (**p = 0.02**, Q = 8.95, df = 9.00, p for heterogeneity = 0.44; I^2 = 0.0%)					0.84 [0.72, 0.97]
**Unstable CAD studies (p = 0.001, Q = 29.79, df = 27.00, p for heterogeneity = 0.32; I^2 = 0.0%)**					0.84 [0.75, 0.93]
Stable CAD					
ACME 1, 1992	0	105	1	107	0.34 [0.01, 8.24]
MASS 1, 1995	1	72	0	72	3.00 [0.12, 72.44]
ACME 2, 1997	25	166	25	162	0.98 [0.59, 1.63]
RITA 2, 1997	11	504	7	514	1.60 [0.63, 4.10]
ACIP, 1997	2	192	20	366	0.19 [0.05, 0.81]
AVERT, 1999	1	177	1	164	0.93 [0.06, 14.69]
DEFER, 2001	2	90	4	91	0.51 [0.09, 2.69]
MASS 2, 2004	9	205	3	203	2.97 [0.82, 10.82]
Hambrecht, 2004	0	50	0	51	1.02 [0.02, 50.41]
COURAGE, 2007	85	1149	95	1138	0.89 [0.67, 1.17]
JSAP, 2008	6	188	7	191	0.87 [0.30, 2.54]
BARI 2D, 2009	102	798	96	807	1.07 [0.83, 1.39]
FAME 2, 2012	1	447	3	441	0.33 [0.03, 3.15]
**Stable CAD studies (p = 0.80, Q = 12.24, df = 12.00, p for heterogeneity = 0.43; I^2 = 0.0%)**					0.98 [0.83, 1.16]

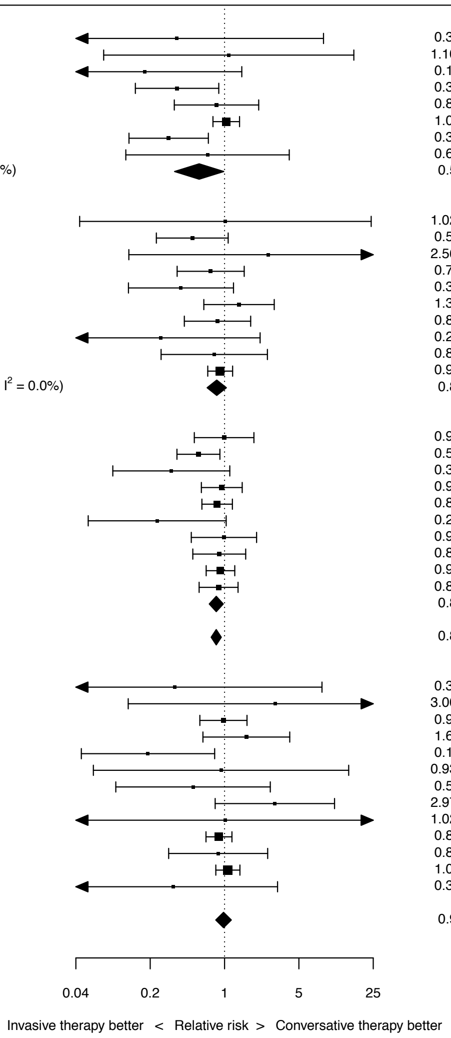

Online Figure 54. Sensitivity analysis for primary outcome of all-cause mortality excluding the TIMI IIIB trial

Study and Year	Active Events	Active N	Control Events	Control N	Relative risk [95% CI]
Unstable CAD – Unrevascularized post–MI					
TOPS, 1992	0	42	1	45	0.36 [0.01, 8.52]
DAKIK, 1998	1	21	1	23	1.10 [0.07, 16.43]
Horie, 1998	1	44	5	39	0.18 [0.02, 1.45]
ALKK, 2003	6	149	17	151	0.36 [0.15, 0.88]
DECOPI, 2004	8	109	9	103	0.84 [0.34, 2.09]
OAT, 2006	87	1082	84	1084	1.04 [0.78, 1.38]
SWISSI 2, 2007	6	96	22	105	0.30 [0.13, 0.70]
VIAMI, 2012	2	106	3	110	0.69 [0.12, 4.06]
Unrevascularized post–MI studies (**p = 0.04**, Q = 13.52, df = 7, p for heterogeneity = 0.06; I^2 = 48.3%)					0.58 [0.34, 0.98]
Unstable CAD – Multivessel disease following STEMI					
Help–AMI, 2009	1	52	0	17	1.02 [0.04, 23.91]
Politi, 2010	10	130	13	84	0.50 [0.23, 1.08]
Dambrink, 2012	2	79	0	40	2.56 [0.13, 52.14]
PRAMI, 2013	12	234	16	231	0.74 [0.36, 1.53]
CvLPRIT, 2015	4	150	10	146	0.39 [0.12, 1.21]
DANAMI 3, 2015	15	313	11	314	1.37 [0.64, 2.93]
Zhang, 2015	13	215	15	213	0.86 [0.42, 1.76]
Hamza, 2016	1	50	4	50	0.25 [0.03, 2.16]
Compare ACUTE, 2017	4	295	10	590	0.80 [0.25, 2.53]
Complete, 2019	96	2016	106	2025	0.91 [0.70, 1.19]
Multivessel disease following STEMI studies (**p = 0.11**, Q = 7.30, df = 9, p for heterogeneity = 0.61; I^2 = 0.0%)					0.84 [0.69, 1.04]
Unstable CAD – NSTEMI					
FRISC II, 2000	27	1222	48	1234	0.57 [0.36, 0.90]
TRUCS, 2000	3	76	9	72	0.32 [0.09, 1.12]
TACTICS 18, 2001	37	1114	39	1106	0.94 [0.61, 1.47]
RITA 3, 2002	60	895	72	915	0.85 [0.61, 1.19]
VINO, 2002	2	64	9	67	0.23 [0.05, 1.04]
ICTUS, 2005	15	604	15	596	0.99 [0.49, 2.00]
Savonitto, 2012	19	154	22	159	0.89 [0.50, 1.58]
After 80, 2016	57	229	62	228	0.92 [0.67, 1.25]
Sanchis, 2016	22	52	26	54	0.88 [0.58, 1.34]
NSTEMI studies (**p = 0.02**, Q = 8.66, df = 8.00, p for heterogeneity = 0.37; I^2 = 0.0%)					0.83 [0.71, 0.97]
**Unstable CAD studies (p = 0.001, Q = 29.51, df = 26.00, p for heterogeneity = 0.29; I^2 = 1.7%)**					0.83 [0.74, 0.93]
Stable CAD					
ACME 1, 1992	0	105	1	107	0.34 [0.01, 8.24]
MASS 1, 1995	1	72	0	72	3.00 [0.12, 72.44]
ACME 2, 1997	25	166	25	162	0.98 [0.59, 1.63]
RITA 2, 1997	11	504	7	514	1.60 [0.63, 4.10]
ACIP, 1997	2	192	20	366	0.19 [0.05, 0.81]
AVERT, 1999	1	177	1	164	0.93 [0.06, 14.69]
DEFER, 2001	2	90	4	91	0.51 [0.09, 2.69]
TIME, 2001	13	153	6	148	2.10 [0.82, 5.37]
MASS 2, 2004	9	205	3	203	2.97 [0.82, 10.82]
Hambrecht, 2004	0	50	0	51	1.02 [0.02, 50.41]
COURAGE, 2007	85	1149	95	1138	0.89 [0.67, 1.17]
JSAP, 2008	6	188	7	191	0.87 [0.30, 2.54]
BARI 2D, 2009	102	798	96	807	1.07 [0.83, 1.39]
FAME 2, 2012	1	447	3	441	0.33 [0.03, 3.15]
**Stable CAD studies (p = 0.98, Q = 14.69, df = 13.00, p for heterogeneity = 0.33; I^2 = 0.0%)**					1.00 [0.85, 1.18]

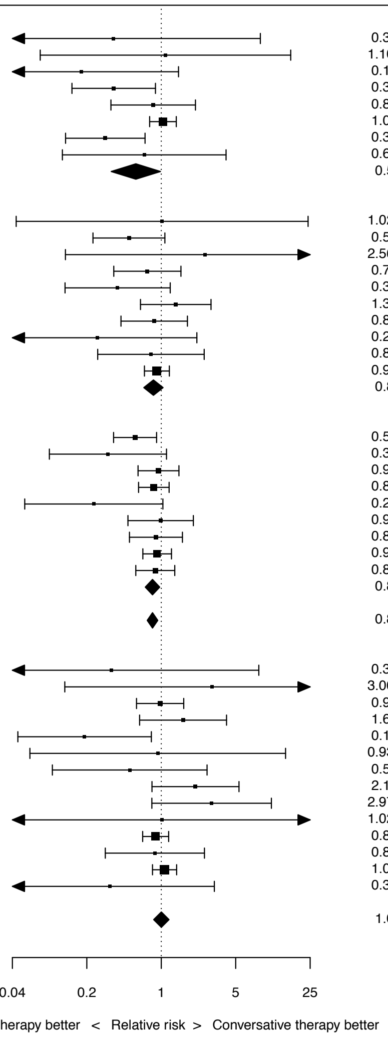

Online Figure 55. Sensitivity analysis for primary outcome of all-cause mortality excluding the TOPS trial

Study and Year	Active Events	Active N	Control Events	Control N	Relative risk [95% CI]
Unstable CAD – Unrevascularized post–MI					
DAKIK, 1998	1	21	1	23	1.10 [0.07, 16.43]
Horie, 1998	1	44	5	39	0.18 [0.02, 1.45]
ALKK, 2003	6	149	17	151	0.36 [0.15, 0.88]
DECOPI, 2004	8	109	9	103	0.84 [0.34, 2.09]
OAT, 2006	87	1082	84	1084	1.04 [0.78, 1.38]
SWISSI 2, 2007	6	96	22	105	0.30 [0.13, 0.70]
VIAMI, 2012	2	106	3	110	0.69 [0.12, 4.06]
Unrevascularized post–MI studies (**p = 0.05**, Q = 13.25, df = 6, p for heterogeneity = 0.04; I^2 = 52.4%)					0.58 [0.34, 1.01]
Unstable CAD – Multivessel disease following STEMI					
Help–AMI, 2009	1	52	0	17	1.02 [0.04, 23.91]
Politi, 2010	10	130	13	84	0.50 [0.23, 1.08]
Dambrink, 2012	2	79	0	40	2.56 [0.13, 52.14]
PRAMI, 2013	12	234	16	231	0.74 [0.36, 1.53]
CvLPRIT, 2015	4	150	10	146	0.39 [0.12, 1.21]
DANAMI 3, 2015	15	313	11	314	1.37 [0.64, 2.93]
Zhang, 2015	13	215	15	213	0.86 [0.42, 1.76]
Hamza, 2016	1	50	4	50	0.25 [0.03, 2.16]
Compare ACUTE, 2017	4	295	10	590	0.80 [0.25, 2.53]
Complete, 2019	96	2016	106	2025	0.91 [0.70, 1.19]
Multivessel disease following STEMI studies (**p = 0.11**, Q = 7.30, df = 9, p for heterogeneity = 0.61; I^2 = 0.0%)					0.84 [0.69, 1.04]
Unstable CAD – NSTEMI					
TIMI IIIB, 1994	18	740	18	733	0.99 [0.52, 1.89]
FRISC II, 2000	27	1222	48	1234	0.57 [0.36, 0.90]
TRUCS, 2000	3	76	9	72	0.32 [0.09, 1.12]
TACTICS 18, 2001	37	1114	39	1106	0.94 [0.61, 1.47]
RITA 3, 2002	60	895	72	915	0.85 [0.61, 1.19]
VINO, 2002	2	64	9	67	0.23 [0.05, 1.04]
ICTUS, 2005	15	604	15	596	0.99 [0.49, 2.00]
Savonitto, 2012	19	154	22	159	0.89 [0.50, 1.58]
After 80, 2016	57	229	62	228	0.92 [0.67, 1.25]
Sanchis, 2016	22	52	26	54	0.88 [0.58, 1.34]
NSTEMI studies (**p = 0.02**, Q = 8.95, df = 9.00, p for heterogeneity = 0.44; I^2 = 0.0%)					0.84 [0.72, 0.97]
**Unstable CAD studies (p = 0.001, Q = 29.51, df = 26.00, p for heterogeneity = 0.29; I^2 = 0.0%)**					0.84 [0.75, 0.93]
Stable CAD					
ACME 1, 1992	0	105	1	107	0.34 [0.01, 8.24]
MASS 1, 1995	1	72	0	72	3.00 [0.12, 72.44]
ACME 2, 1997	25	166	25	162	0.98 [0.59, 1.63]
RITA 2, 1997	11	504	7	514	1.60 [0.63, 4.10]
ACIP, 1997	2	192	20	366	0.19 [0.05, 0.81]
AVERT, 1999	1	177	1	164	0.93 [0.06, 14.69]
DEFER, 2001	2	90	4	91	0.51 [0.09, 2.69]
TIME, 2001	13	153	6	148	2.10 [0.82, 5.37]
MASS 2, 2004	9	205	3	203	2.97 [0.82, 10.82]
Hambrecht, 2004	0	50	0	51	1.02 [0.02, 50.41]
COURAGE, 2007	85	1149	95	1138	0.89 [0.67, 1.17]
JSAP, 2008	6	188	7	191	0.87 [0.30, 2.54]
BARI 2D, 2009	102	798	96	807	1.07 [0.83, 1.39]
FAME 2, 2012	1	447	3	441	0.33 [0.03, 3.15]
**Stable CAD studies (p = 0.98, Q = 14.69, df = 13.00, p for heterogeneity = 0.33; I^2 = 0.0%)**					1.00 [0.85, 1.18]

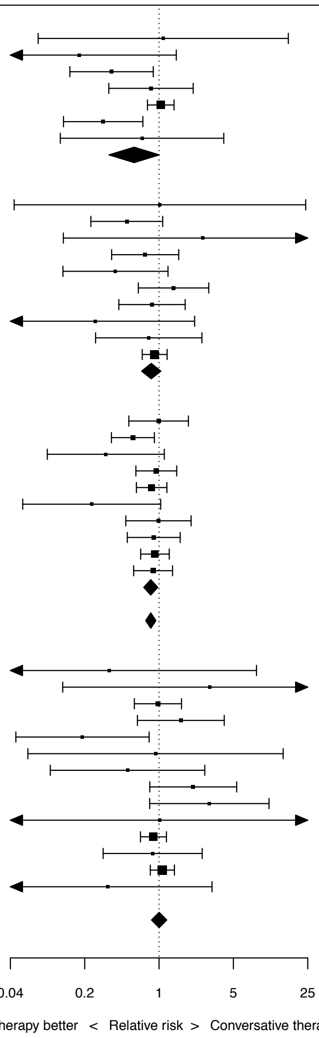

Online Figure 56. Sensitivity analysis for primary outcome of all-cause mortality excluding the TRUCS trial

Study and Year	Active Events	Active N	Control Events	Control N	Relative risk [95% CI]
Unstable CAD – Unrevascularized post–MI					
TOPS, 1992	0	42	1	45	0.36 [0.01, 8.52]
DAKIK, 1998	1	21	1	23	1.10 [0.07, 16.43]
Horie, 1998	1	44	5	39	0.18 [0.02, 1.45]
ALKK, 2003	6	149	17	151	0.36 [0.15, 0.88]
DECOPI, 2004	8	109	9	103	0.84 [0.34, 2.09]
OAT, 2006	87	1082	84	1084	1.04 [0.78, 1.38]
SWISSI 2, 2007	6	96	22	105	0.30 [0.13, 0.70]
VIAMI, 2012	2	106	3	110	0.69 [0.12, 4.06]
Unrevascularized post–MI studies (**p = 0.04**, Q = 13.52, df = 7, p for heterogeneity = 0.06; I^2 = 48.3%)					0.58 [0.34, 0.98]
Unstable CAD – Multivessel disease following STEMI					
Help–AMI, 2009	1	52	0	17	1.02 [0.04, 23.91]
Politi, 2010	10	130	13	84	0.50 [0.23, 1.08]
Dambrink, 2012	2	79	0	40	2.56 [0.13, 52.14]
PRAMI, 2013	12	234	16	231	0.74 [0.36, 1.53]
CvLPRIT, 2015	4	150	10	146	0.39 [0.12, 1.21]
DANAMI 3, 2015	15	313	11	314	1.37 [0.64, 2.93]
Zhang, 2015	13	215	15	213	0.86 [0.42, 1.76]
Hamza, 2016	1	50	4	50	0.25 [0.03, 2.16]
Compare ACUTE, 2017	4	295	10	590	0.80 [0.25, 2.53]
Complete, 2019	96	2016	106	2025	0.91 [0.70, 1.19]
Multivessel disease following STEMI studies (**p = 0.11**, Q = 7.30, df = 9, p for heterogeneity = 0.61; I^2 = 0.0%)					0.84 [0.69, 1.04]
Unstable CAD – NSTEMI					
TIMI IIIB, 1994	18	740	18	733	0.99 [0.52, 1.89]
FRISC II, 2000	27	1222	48	1234	0.57 [0.36, 0.90]
TACTICS 18, 2001	37	1114	39	1106	0.94 [0.61, 1.47]
RITA 3, 2002	60	895	72	915	0.85 [0.61, 1.19]
VINO, 2002	2	64	9	67	0.23 [0.05, 1.04]
ICTUS, 2005	15	604	15	596	0.99 [0.49, 2.00]
Savonitto, 2012	19	154	22	159	0.89 [0.50, 1.58]
After 80, 2016	57	229	62	228	0.92 [0.67, 1.25]
Sanchis, 2016	22	52	26	54	0.88 [0.58, 1.34]
NSTEMI studies (**p = 0.03**, Q = 6.64, df = 8.00, p for heterogeneity = 0.58; I^2 = 0.0%)					0.85 [0.73, 0.99]
**Unstable CAD studies (p = 0.002, Q = 27.50, df = 26.00, p for heterogeneity = 0.38; I^2 = 0.0%)**					0.84 [0.75, 0.94]
Stable CAD					
ACME 1, 1992	0	105	1	107	0.34 [0.01, 8.24]
MASS 1, 1995	1	72	0	72	3.00 [0.12, 72.44]
ACME 2, 1997	25	166	25	162	0.98 [0.59, 1.63]
RITA 2, 1997	11	504	7	514	1.60 [0.63, 4.10]
ACIP, 1997	2	192	20	366	0.19 [0.05, 0.81]
AVERT, 1999	1	177	1	164	0.93 [0.06, 14.69]
DEFER, 2001	2	90	4	91	0.51 [0.09, 2.69]
TIME, 2001	13	153	6	148	2.10 [0.82, 5.37]
MASS 2, 2004	9	205	3	203	2.97 [0.82, 10.82]
Hambrecht, 2004	0	50	0	51	1.02 [0.02, 50.41]
COURAGE, 2007	85	1149	95	1138	0.89 [0.67, 1.17]
JSAP, 2008	6	188	7	191	0.87 [0.30, 2.54]
BARI 2D, 2009	102	798	96	807	1.07 [0.83, 1.39]
FAME 2, 2012	1	447	3	441	0.33 [0.03, 3.15]
**Stable CAD studies (p = 0.98, Q = 14.69, df = 13.00, p for heterogeneity = 0.33; I^2 = 0.0%)**					1.00 [0.85, 1.18]

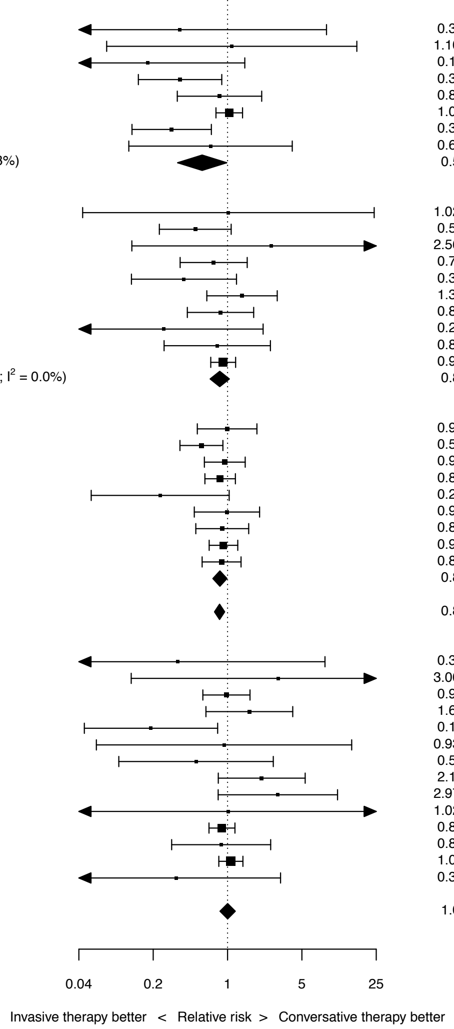

Online Figure 57. Sensitivity analysis for primary outcome of all-cause mortality excluding the VIAMI trial

Study and Year	Active Events	Active N	Control Events	Control N	Relative risk [95% CI]
Unstable CAD – Unrevascularized post–MI					
TOPS, 1992	0	42	1	45	0.36 [0.01, 8.52]
DAKIK, 1998	1	21	1	23	1.10 [0.07, 16.43]
Horie, 1998	1	44	5	39	0.18 [0.02, 1.45]
ALKK, 2003	6	149	17	151	0.36 [0.15, 0.88]
DECOPI, 2004	8	109	9	103	0.84 [0.34, 2.09]
OAT, 2006	87	1082	84	1084	1.04 [0.78, 1.38]
SWISSI 2, 2007	6	96	22	105	0.30 [0.13, 0.70]
Unrevascularized post–MI studies (**p = 0.05**, Q = 13.48, df = 6, p for heterogeneity = 0.04; I^2 = 54.1%)					0.56 [0.32, 1.00]
Unstable CAD – Multivessel disease following STEMI					
Help–AMI, 2009	1	52	0	17	1.02 [0.04, 23.91]
Politi, 2010	10	130	13	84	0.50 [0.23, 1.08]
Dambrink, 2012	2	79	0	40	2.56 [0.13, 52.14]
PRAMI, 2013	12	234	16	231	0.74 [0.36, 1.53]
CvLPRIT, 2015	4	150	10	146	0.39 [0.12, 1.21]
DANAMI 3, 2015	15	313	11	314	1.37 [0.64, 2.93]
Zhang, 2015	13	215	15	213	0.86 [0.42, 1.76]
Hamza, 2016	1	50	4	50	0.25 [0.03, 2.16]
Compare ACUTE, 2017	4	295	10	590	0.80 [0.25, 2.53]
Complete, 2019	96	2016	106	2025	0.91 [0.70, 1.19]
Multivessel disease following STEMI studies (**p = 0.11**, Q = 7.30, df = 9, p for heterogeneity = 0.61; I^2 = 0.0%)					0.84 [0.69, 1.04]
Unstable CAD – NSTEMI					
TIMI IIIB, 1994	18	740	18	733	0.99 [0.52, 1.89]
FRISC II, 2000	27	1222	48	1234	0.57 [0.36, 0.90]
TRUCS, 2000	3	76	9	72	0.32 [0.09, 1.12]
TACTICS 18, 2001	37	1114	39	1106	0.94 [0.61, 1.47]
RITA 3, 2002	60	895	72	915	0.85 [0.61, 1.19]
VINO, 2002	2	64	9	67	0.23 [0.05, 1.04]
ICTUS, 2005	15	604	15	596	0.99 [0.49, 2.00]
Savonitto, 2012	19	154	22	159	0.89 [0.50, 1.58]
After 80, 2016	57	229	62	228	0.92 [0.67, 1.25]
Sanchis, 2016	22	52	26	54	0.88 [0.58, 1.34]
NSTEMI studies (**p = 0.02**, Q = 8.95, df = 9.00, p for heterogeneity = 0.44; I^2 = 0.0%)					0.84 [0.72, 0.97]
**Unstable CAD studies (p = 0.001, Q = 29.75, df = 26.00, p for heterogeneity = 0.28; I^2 = 0.0%)**					0.84 [0.75, 0.93]
Stable CAD					
ACME 1, 1992	0	105	1	107	0.34 [0.01, 8.24]
MASS 1, 1995	1	72	0	72	3.00 [0.12, 72.44]
ACME 2, 1997	25	166	25	162	0.98 [0.59, 1.63]
RITA 2, 1997	11	504	7	514	1.60 [0.63, 4.10]
ACIP, 1997	2	192	20	366	0.19 [0.05, 0.81]
AVERT, 1999	1	177	1	164	0.93 [0.06, 14.69]
DEFER, 2001	2	90	4	91	0.51 [0.09, 2.69]
TIME, 2001	13	153	6	148	2.10 [0.82, 5.37]
MASS 2, 2004	9	205	3	203	2.97 [0.82, 10.82]
Hambrecht, 2004	0	50	0	51	1.02 [0.02, 50.41]
COURAGE, 2007	85	1149	95	1138	0.89 [0.67, 1.17]
JSAP, 2008	6	188	7	191	0.87 [0.30, 2.54]
BARI 2D, 2009	102	798	96	807	1.07 [0.83, 1.39]
FAME 2, 2012	1	447	3	441	0.33 [0.03, 3.15]
**Stable CAD studies (p = 0.98, Q = 14.69, df = 13.00, p for heterogeneity = 0.33; I^2 = 0.0%)**					1.00 [0.85, 1.18]

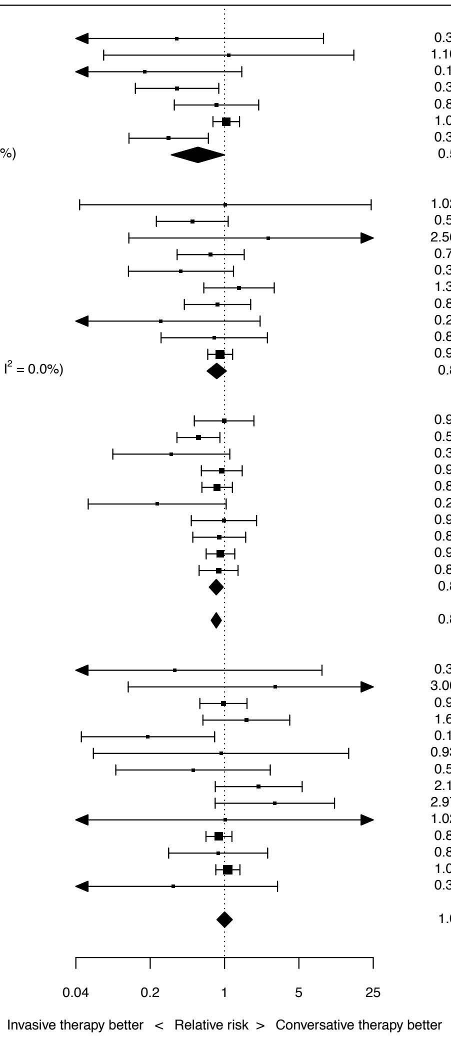

Online Figure 58. Sensitivity analysis for primary outcome of all-cause mortality excluding the VINO trial

Study and Year	Active Events	Active N	Control Events	Control N	Relative risk [95% CI]
Unstable CAD – Unrevascularized post–MI					
TOPS, 1992	0	42	1	45	0.36 [0.01, 8.52]
DAKIK, 1998	1	21	1	23	1.10 [0.07, 16.43]
Horie, 1998	1	44	5	39	0.18 [0.02, 1.45]
ALKK, 2003	6	149	17	151	0.36 [0.15, 0.88]
DECOPI, 2004	8	109	9	103	0.84 [0.34, 2.09]
OAT, 2006	87	1082	84	1084	1.04 [0.78, 1.38]
SWISSI 2, 2007	6	96	22	105	0.30 [0.13, 0.70]
VIAMI, 2012	2	106	3	110	0.69 [0.12, 4.06]
Unrevascularized post–MI studies (**p = 0.04**, Q = 13.52, df = 7, p for heterogeneity = 0.06; I^2 = 48.3%)					0.58 [0.34, 0.98]
Unstable CAD – Multivessel disease following STEMI					
Help–AMI, 2009	1	52	0	17	1.02 [0.04, 23.91]
Politi, 2010	10	130	13	84	0.50 [0.23, 1.08]
Dambrink, 2012	2	79	0	40	2.56 [0.13, 52.14]
PRAMI, 2013	12	234	16	231	0.74 [0.36, 1.53]
CvLPRIT, 2015	4	150	10	146	0.39 [0.12, 1.21]
DANAMI 3, 2015	15	313	11	314	1.37 [0.64, 2.93]
Zhang, 2015	13	215	15	213	0.86 [0.42, 1.76]
Hamza, 2016	1	50	4	50	0.25 [0.03, 2.16]
Compare ACUTE, 2017	4	295	10	590	0.80 [0.25, 2.53]
Complete, 2019	96	2016	106	2025	0.91 [0.70, 1.19]
Multivessel disease following STEMI studies (**p = 0.11**, Q = 7.30, df = 9, p for heterogeneity = 0.61; I^2 = 0.0%)					0.84 [0.69, 1.04]
Unstable CAD – NSTEMI					
TIMI IIIB, 1994	18	740	18	733	0.99 [0.52, 1.89]
FRISC II, 2000	27	1222	48	1234	0.57 [0.36, 0.90]
TRUCS, 2000	3	76	9	72	0.32 [0.09, 1.12]
TACTICS 18, 2001	37	1114	39	1106	0.94 [0.61, 1.47]
RITA 3, 2002	60	895	72	915	0.85 [0.61, 1.19]
ICTUS, 2005	15	604	15	596	0.99 [0.49, 2.00]
Savonitto, 2012	19	154	22	159	0.89 [0.50, 1.58]
After 80, 2016	57	229	62	228	0.92 [0.67, 1.25]
Sanchis, 2016	22	52	26	54	0.88 [0.58, 1.34]
NSTEMI studies (**p = 0.03**, Q = 6.10, df = 8.00, p for heterogeneity = 0.64; I^2 = 0.0%)					0.85 [0.73, 0.99]
**Unstable CAD studies (p = 0.002, Q = 26.96, df = 26.00, p for heterogeneity = 0.41; I^2 = 0.0%)**					0.84 [0.75, 0.94]
Stable CAD					
ACME 1, 1992	0	105	1	107	0.34 [0.01, 8.24]
MASS 1, 1995	1	72	0	72	3.00 [0.12, 72.44]
ACME 2, 1997	25	166	25	162	0.98 [0.59, 1.63]
RITA 2, 1997	11	504	7	514	1.60 [0.63, 4.10]
ACIP, 1997	2	192	20	366	0.19 [0.05, 0.81]
AVERT, 1999	1	177	1	164	0.93 [0.06, 14.69]
DEFER, 2001	2	90	4	91	0.51 [0.09, 2.69]
TIME, 2001	13	153	6	148	2.10 [0.82, 5.37]
MASS 2, 2004	9	205	3	203	2.97 [0.82, 10.82]
Hambrecht, 2004	0	50	0	51	1.02 [0.02, 50.41]
COURAGE, 2007	85	1149	95	1138	0.89 [0.67, 1.17]
JSAP, 2008	6	188	7	191	0.87 [0.30, 2.54]
BARI 2D, 2009	102	798	96	807	1.07 [0.83, 1.39]
FAME 2, 2012	1	447	3	441	0.33 [0.03, 3.15]
**Stable CAD studies (p = 0.98, Q = 14.69, df = 13.00, p for heterogeneity = 0.33; I^2 = 0.0%)**					1.00 [0.85, 1.18]

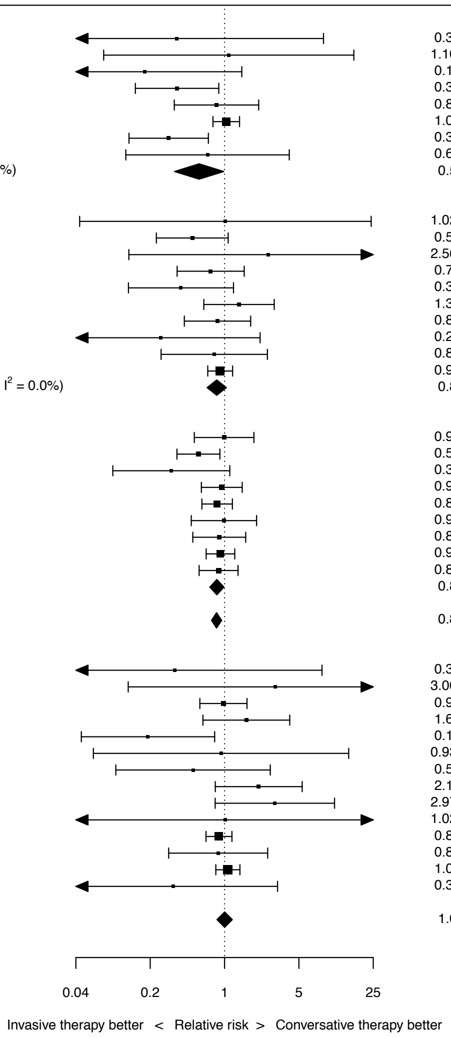

Online Figure 59. Sensitivity analysis for primary outcome of all-cause mortality excluding the Zhang trial

Study and Year	Active Events	N	Control Events	N	Relative risk [95% CI]
Unstable CAD – Unrevascularized post–MI					
TOPS, 1992	0	42	1	45	0.36 [0.01, 8.52]
DAKIK, 1998	1	21	1	23	1.10 [0.07, 16.43]
Horie, 1998	1	44	5	39	0.18 [0.02, 1.45]
ALKK, 2003	6	149	17	151	0.36 [0.15, 0.88]
DECOPI, 2004	8	109	9	103	0.84 [0.34, 2.09]
OAT, 2006	87	1082	84	1084	1.04 [0.78, 1.38]
SWISSI 2, 2007	6	96	22	105	0.30 [0.13, 0.70]
VIAMI, 2012	2	106	3	110	0.69 [0.12, 4.06]
Unrevascularized post–MI studies (**p = 0.04**, Q = 13.52, df = 7, p for heterogeneity = 0.06; I^2 = 48.3%)					0.58 [0.34, 0.98]
Unstable CAD – Multivessel disease following STEMI					
Help–AMI, 2009	1	52	0	17	1.02 [0.04, 23.91]
Politi, 2010	10	130	13	84	0.50 [0.23, 1.08]
Dambrink, 2012	2	79	0	40	2.56 [0.13, 52.14]
PRAMI, 2013	12	234	16	231	0.74 [0.36, 1.53]
CvLPRIT, 2015	4	150	10	146	0.39 [0.12, 1.21]
DANAMI 3, 2015	15	313	11	314	1.37 [0.64, 2.93]
Hamza, 2016	1	50	4	50	0.25 [0.03, 2.16]
Compare ACUTE, 2017	4	295	10	590	0.80 [0.25, 2.53]
Complete, 2019	96	2016	106	2025	0.91 [0.70, 1.19]
Multivessel disease following STEMI studies (**p = 0.13**, Q = 7.29, df = 8, p for heterogeneity = 0.51; I^2 = 1.3%)					0.84 [0.67, 1.05]
Unstable CAD – NSTEMI					
TIMI IIIB, 1994	18	740	18	733	0.99 [0.52, 1.89]
FRISC II, 2000	27	1222	48	1234	0.57 [0.36, 0.90]
TRUCS, 2000	3	76	9	72	0.32 [0.09, 1.12]
TACTICS 18, 2001	37	1114	39	1106	0.94 [0.61, 1.47]
RITA 3, 2002	60	895	72	915	0.85 [0.61, 1.19]
VINO, 2002	2	64	9	67	0.23 [0.05, 1.04]
ICTUS, 2005	15	604	15	596	0.99 [0.49, 2.00]
Savonitto, 2012	19	154	22	159	0.89 [0.50, 1.58]
After 80, 2016	57	229	62	228	0.92 [0.67, 1.25]
Sanchis, 2016	22	52	26	54	0.88 [0.58, 1.34]
NSTEMI studies (**p = 0.02**, Q = 8.95, df = 9.00, p for heterogeneity = 0.44; I^2 = 0.0%)					0.84 [0.72, 0.97]
**Unstable CAD studies (p = 0.001, Q = 29.78, df = 26.00, p for heterogeneity = 0.28; I^2 = 0.0%)**					0.83 [0.75, 0.93]
Stable CAD					
ACME 1, 1992	0	105	1	107	0.34 [0.01, 8.24]
MASS 1, 1995	1	72	0	72	3.00 [0.12, 72.44]
ACME 2, 1997	25	166	25	162	0.98 [0.59, 1.63]
RITA 2, 1997	11	504	7	514	1.60 [0.63, 4.10]
ACIP, 1997	2	192	20	366	0.19 [0.05, 0.81]
AVERT, 1999	1	177	1	164	0.93 [0.06, 14.69]
DEFER, 2001	2	90	4	91	0.51 [0.09, 2.69]
TIME, 2001	13	153	6	148	2.10 [0.82, 5.37]
MASS 2, 2004	9	205	3	203	2.97 [0.82, 10.82]
Hambrecht, 2004	0	50	0	51	1.02 [0.02, 50.41]
COURAGE, 2007	85	1149	95	1138	0.89 [0.67, 1.17]
JSAP, 2008	6	188	7	191	0.87 [0.30, 2.54]
BARI 2D, 2009	102	798	96	807	1.07 [0.83, 1.39]
FAME 2, 2012	1	447	3	441	0.33 [0.03, 3.15]
**Stable CAD studies (p = 0.98, Q = 14.69, df = 13.00, p for heterogeneity = 0.33; I^2 = 0.0%)**					1.00 [0.85, 1.18]

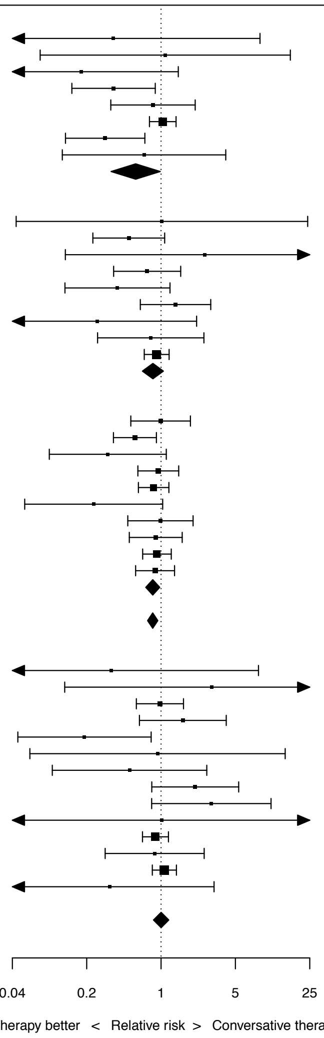

Supplement: Supplementary file 1 [file hcq-13-e006363-s001.pdf]
